# Supplementary material for: Single-Cell Analysis Suggests that Ongoing Affinity Maturation Drives the Emergence of Pemphigus Vulgaris Autoimmune Disease
Source: Cell Rep. Author manuscript; Available in PMC 2019 Aug 6. (PMC6684256; doi:10.1016/j.celrep.2019.06.066)
Supplement: 1 [file NIHMS1535565-supplement-1.pdf]

Cell Reports, Volume *28*

Supplemental Information

Single-Cell Analysis Suggests that Ongoing Affinity Maturation Drives the Emergence of Pemphigus Vulgaris Autoimmune Disease

Alice Cho, Amber L. Caldara, Nina A. Ran, Zach Menne, Robert C. Kauffman, Maurizio Affer, Alexandra Llovet, Carson Norwood, Aaron Scanlan, Grace Mantus, Bridget Bradley, Stephanie Zimmer, Thomas Schmidt, Michael Hertl, Aimee S. Payne, Ron Feldman, Andrew P. Kowalczyk, and Jens Wrammert

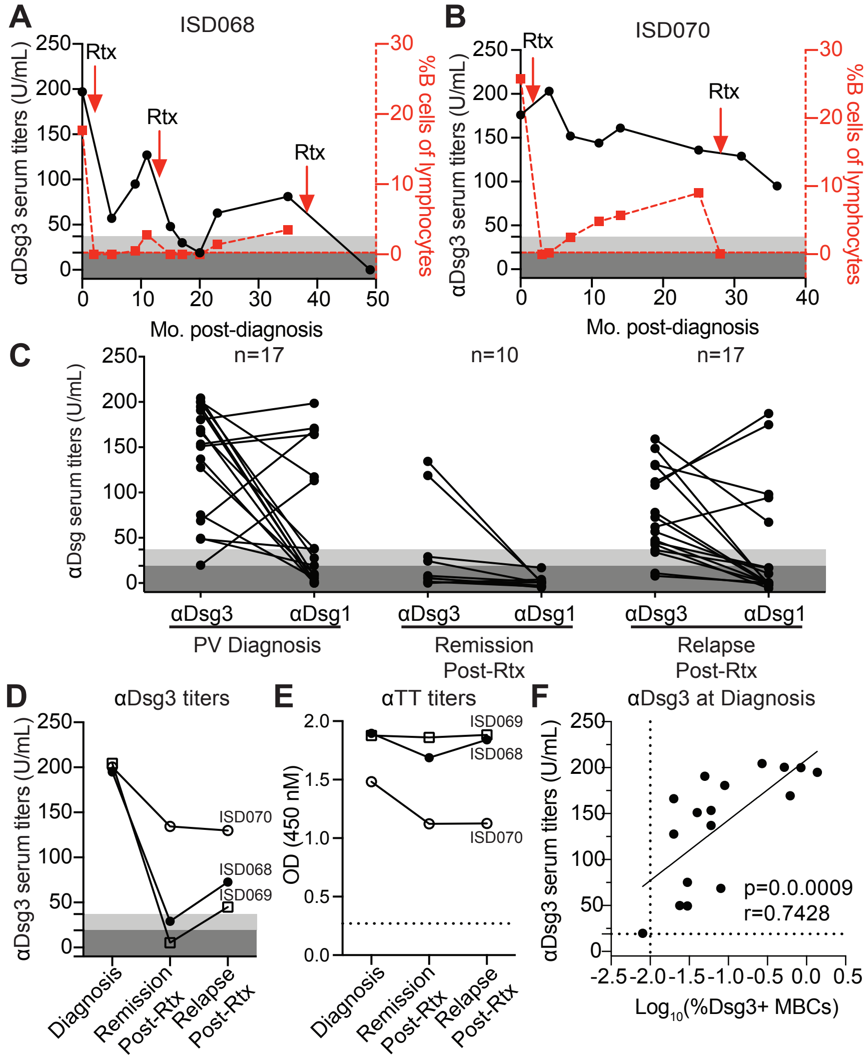

Figure S1. Related to Figure 1. Kinetics of serum antibody titers before and after treatment with B cell depletive therapy. (A) The average kinetics of anti-Dsg3 serum titers (left Y-axis, black line) and peripheral blood CD19+ B cells (right Y-axis, red line) for most PV patients after treatment with Rituximab (Rtx) therapy. Cut-off values for ELISA were determined by manufacturer's suggestion (dark grey = negative, light grey = indeterminate). Red dotted line represents limit of detection for CD19+ B cells in circulation. (B) A smaller number of patients who are in clinical remission do not have a complete decrease of anti-Dsg3 serum titers in response to Rituximab-mediated therapy, despite showing complete depletion of B cells in the periphery. (C) Anti-Dsg1 serum titers were found only in a subset of PV patients, specifically at the time of diagnosis and relapse post-Rtx, but undetectable in patients in remission. When tracking serum titers specifically in PV patients sampled longitudinally at multiple time points (D) anti-Dsg3 serum titers decreased dramatically after Rituximab-treatment while (E) anti-tetanus toxoid (TT) titers did not significantly change over time. Anti-TT titers are shown as the final OD reading of serum diluted at 1:200. Dotted-line represents the cut-off value determined by the background signal in a negative HC serum. Data is representative of two individual experimental repeats. (F) There was a significant positive correlation between frequency of Dsg3-specific MBCs and anti-Dsg3 serum titers. Dotted lines represent cut-off values for either the ELISA or MBC assay. A spearman correlation was used to analyze this data.

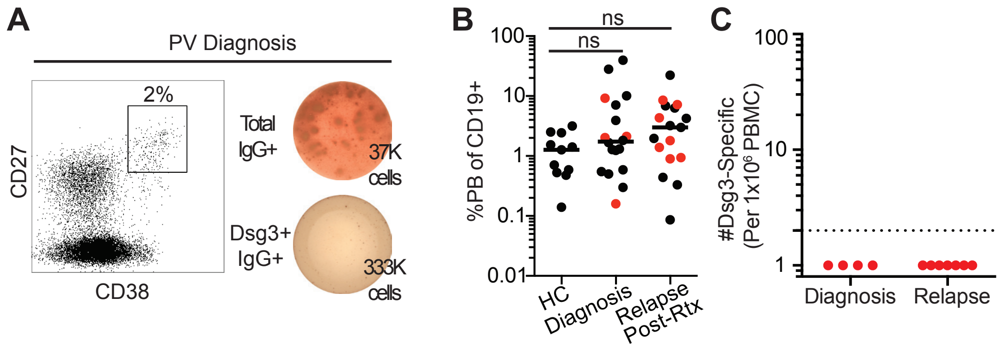

Figure S2. Related to Figure 1. No Dsg3-specific circulating plasmablasts are detected in symptomatic PV patients. (A) A representative flow plot of plasmablasts detected in peripheral blood (gated on CD3-CD19+ lymphocytes) and an ELISPOT assay, showing a single dilution of titrated PBMCs probed for total IgG and Dsg3-specific IgG antibody-secreting cells from a PV patient at the time of diagnosis. (B) Frequency of plasmablasts does not differ between HC and patients at diagnosis or relapse. Red dots represent patients tested by ELISPOT for Dsg3-specific plasmablasts. (C) Absence of detectable Dsg3-specific circulating plasmablasts in patients presenting with active PV disease. Dotted line represents limit of detection of the ELISPOT assay at 3 antibody secreting cells per $1x10^6$ PBMCs. A one-way ANOVA was used to analyze this data.

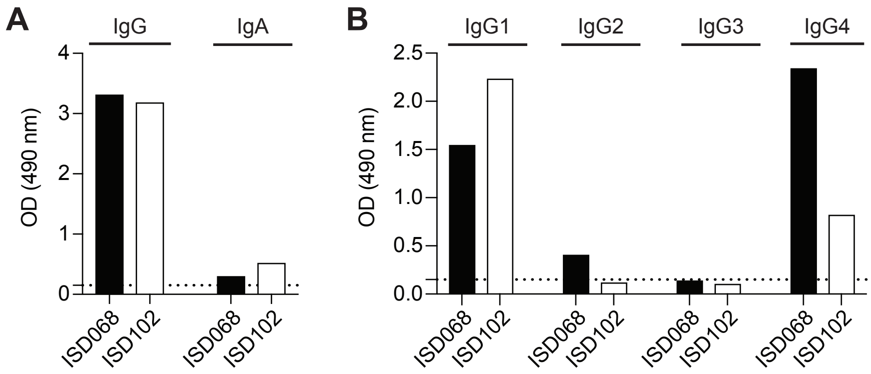

Figure S3. Related to Figure 2. Dsg3-specific serum antibodies were primarily IgG1 and IgG4 isotype. An ELISA was used to determine isotype usage of Dsg3-specific serum antibodies from patients ISD068 and ISD102. Shown is the OD reading of serum diluted at 1:100. (A) Dsg3-specific serum responses are predominantly IgG, although some IgA was detected in both patients. (B) ELISA using IgG subclass reagents show that the Dsg3-specific responses are dominated by IgG1 and IgG4 subclass usage. Little to no IgG2 or IgG3 was detected in either of the patients. Dotted line represents cut-off value determined by the background signal detected in HC serum. Data is representative of two individual experimental repeats.

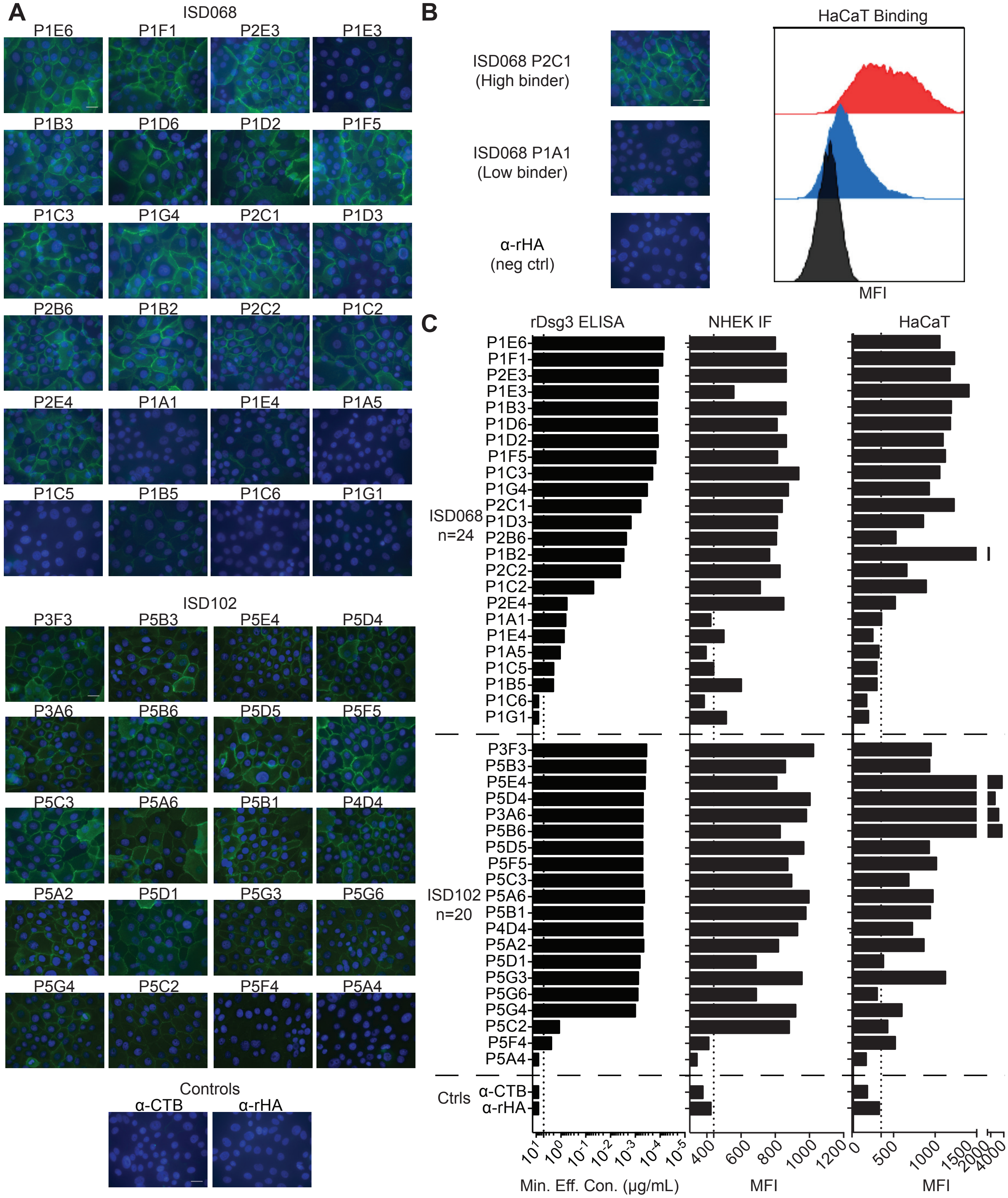

Figure S4. Related to Figure 2. Assessment of mAbs binding to Dsg3 expressed on cell surface. Binding of mAbs to Dsg3 was determined using (A) immunofluorescent staining of HK cells (primary cells line of human keratinocytes) and (B) flow cytometry-based assay of staining HaCaT cells (immortalized cell line of human keratinocytes), shown as histograms. Representative images from panel S4A are used in panel S4B to provide a direct, side-by-side comparison between the two different methods. (C) Summary data comparing binding measured via ELISA, IF, and flow cytometry shows that there is a range of binding activity of mAbs towards Dsg3. Representative data of two individual experimental repeats is shown. White scale bar in A, 25 μm. Dotted line for MFI represents cut-off value for a negative signal.

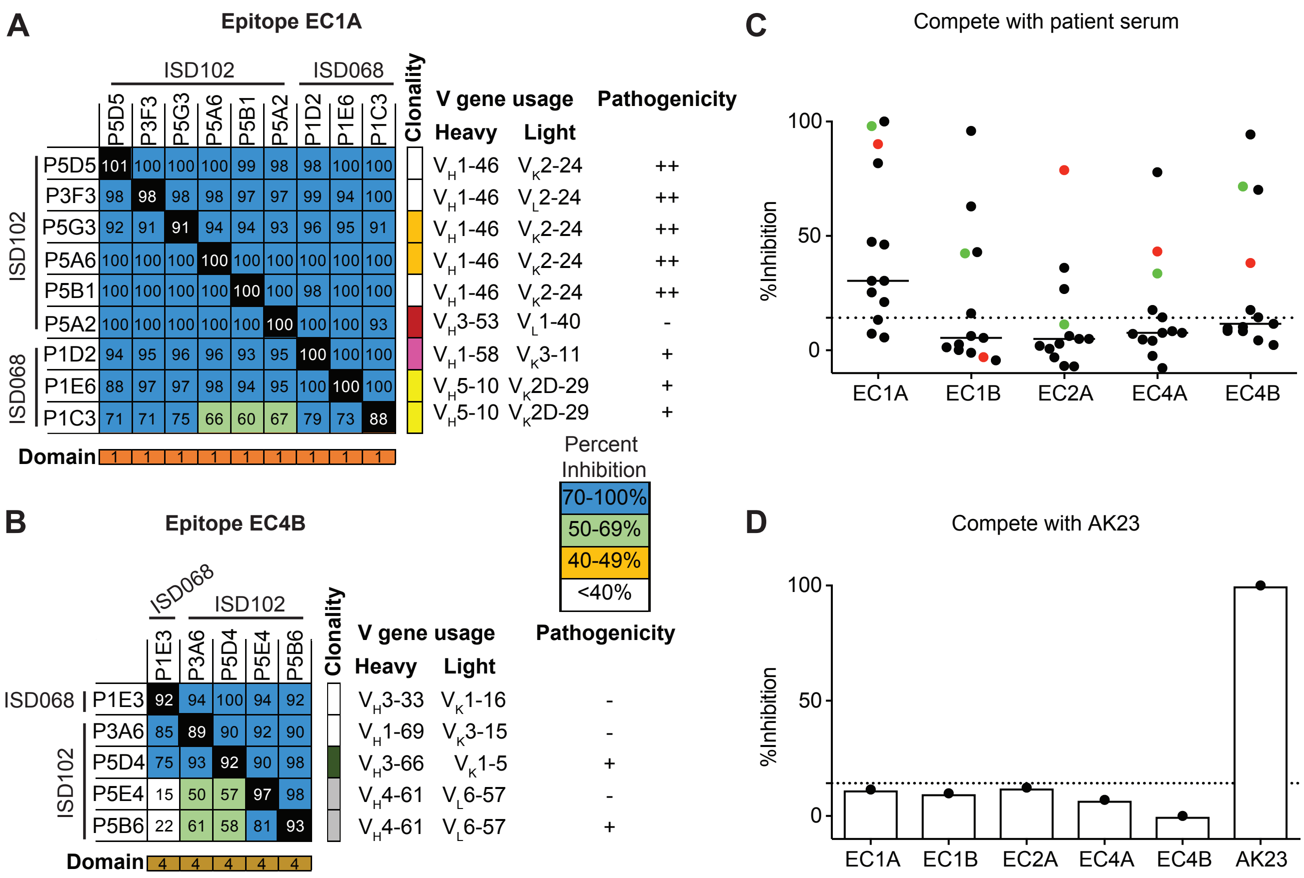

Figure S5. Related to Figure 4. Similar sterically-distinct epitopes detected by flow cytometry-based blocking assay were representative of other PV patients at diagnosis. The flow-based blocking assay was also used to define overlapping epitopes between patients ISD068 and ISD102. Antibodies targeting (A) epitope EC1A and (B) epitope EC4B could be detected in both patients. (C) A blocking ELISA was performed by using 13 PV serum from time of diagnosis to block binding of 5 different biotinylated mAbs to Dsg3, each mAb representative of the 5 detected epitopes described in Figure 4. While EC1A epitope was most commonly detected in all patients, the other 4 epitopes were targeted as well. Red dot: patient ISD068; Green dot: patient ISD102. (D) A blocking ELISA was also used to determine if the 5 detected epitopes bound to the same epitope as AK23, an EC1-specific pathogenic mouse-derived mAb (Tsunoda et al., 2003). Interestingly, none of the 5 described epitopes targeted by the human MBC-derived mAbs bound to the same epitopes as the EC1-specific AK23, suggesting that preferred immunodominant epitopes are different for humans mAbs versus mouse mAbs. Representative data of two individual experimental repeats is shown. Dotted line represents cut-off value for positive inhibition, as determined by the mean of inhibition of 9 HC sera plus 2 SD.

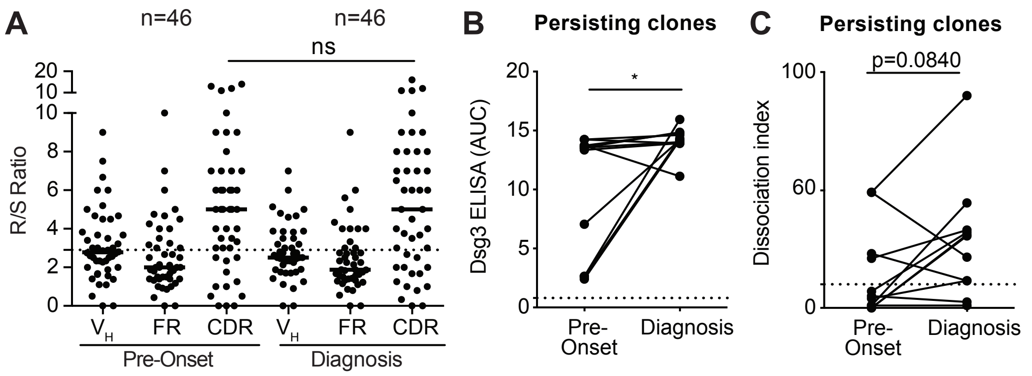

Figure S6. Related to Figure 5. Antigen selection and ongoing affinity maturation of Dsg3-specific memory B cells. (A) R/S ratios were above 2.9 in the CDR at both pre-onset and diagnosis, indicating that antigenic selection is an ongoing process occurring continuously during disease development. When comparing only mAbs derived from persisting clones (MBCs from the same clonal family found at both pre-onset and diagnosis time points), (B) there was a significant increase in relative affinity of mAbs for Dsg3 and (C) a trend towards an increase in pathogenicity from pre-onset to diagnosis. A Mann-Whitney U test or Wilcoxon paired T-test was used where appropriate. * = $P \leq 0.05$

A

ISD068 Pre-onset

		P1C5	P1B2	P2D6	P1F4	P1D5	P1D4	P1D1	P2F2	P1F3	P2G2	P2D3	P2G1	P1G2	P2D2	P1G1	P2D1	P1E3	P2C4	P1B6	P1G3	Clonality	Heavy	Light	Pathogenicity
EC1A	P1C5	90	84	96	94	95	11	84	87	53	0	0	0	0	2	0	0	0	0	5	0		V_H5-10	V_K2D-29	-
EC1A	P1B2	85	92	85	94	88	75	82	58	44	13	0	0	0	8	0	0	0	0	9	0		V_H1-2	V_L1-47	-
EC1A	P2D6	66	90	96	93	91	92	81	83	49	0	6	21	16	60	41	10	29	13	38	18		V_H1-58	V_K3-11	+
EC1A	P1F4	24	62	79	96	96	60	62	53	43	3	6	4	10	29	9	13	12	5	21	0		V_H5-51	V_L3-9	+
EC1A	P1D5	30	86	85	95	96	77	72	78	63	14	19	22	18	47	20	25	31	7	61	6		V_H1-2	V_L1-51	+
EC2A	P1D4	17	93	101	96	97	98	114	93	47	15	21	9	62	94	96	95	92	95	95	13		V_H1-18	V_K1-9	-
EC2A	P1D1	11	54	55	52	87	91	97	96	92	78	65	88	70	86	81	86	84	82	86	0		V_H1-69	V_K3-11	+
EC2A	P2F2	19	22	12	24	39	84	88	96	57	57	26	78	51	95	84	94	77	97	95	4		V_H1-2	V_K3-15	-
EC2A	P1F3	0	0	0	0	54	0	89	94	82	87	84	91	88	89	88	95	85	88	92	0		V_H4-39	V_K1-5	-
EC2A	P2G2	0	0	3	13	50	0	92	90	85	96	92	97	86	97	96	89	93	88	91	5		V_H4-39	V_K1-5	+
EC2A	P2D3	0	0	14	12	56	13	98	95	95	99	97	98	87	87	95	89	97	93	91	13		V_H4-39	V_K1-5	-
EC2A	P2G1	12	30	1	9	30	14	88	96	75	83	48	98	62	90	90	86	86	93	95	8		V_H4-39	V_K3-15	+
EC2A	P1G2	0	0	8	69	88	93	107	96	94	82	90	90	86	85	93	95	85	93	93	3		V_H4-4	V_K3-20	-
EC2A	P2D2	12	52	22	36	62	87	99	94	87	83	83	95	88	91	93	93	91	95	96	10		V_H4-4	V_K3-20	-
EC2A	P1G1	15	17	4	47	75	90	99	95	87	91	74	96	83	95	94	96	96	95	95	0		V_H4-4	V_K3-20	-
EC2A	P2D1	23	21	20	31	68	91	95	94	89	83	82	95	75	93	94	94	86	94	95	18		V_H1-2	V_K3-15	-
EC2A	P1E3	0	5	3	35	67	95	107	95	92	93	78	96	85	95	95	97	95	98	96	12		V_H1-2	V_K3-15	-
EC2A	P2C4	15	23	15	24	49	86	92	93	63	59	10	92	22	96	86	90	79	95	92	4		V_H4-59	V_K3-15	-
EC2A	P1B6	15	20	13	21	49	81	86	96	24	8	6	93	17	89	82	89	74	93	96	2		V_H1-2	V_K3-20	+
Unknown	P1G3	0	11	10	0	0	19	7	4	3	11	9	11	0	0	8	0	0	0	0	100		V_H5-10	V_K1-33	+

B

ISD068 Diagnosis

		P1D2	P1E6	P1C3	P1C2	P1A1	P2E3	P1D6	P1D3	P2C2	P1A2	P1G4	P1B3	P1F5	P1F1	P1B2	P2E4	P2B6	P2C1	P1E3	Clonality	Heavy	Light	Pathogenicity
EC1A	P1D2	100	100	100	91	64	60	57	48	49	61	1	8	2	6	11	25	4	18	2		V_H1-58	V_K3-11	+
EC1A	P1E6	100	100	100	94	11	7	4	1	9	17	0	3	3	1	10	9	9	9	2		V_H5-10	V_K2D-29	+
EC1A	P1C3	79	73	88	69	1	12	7	13	20	7	2	14	3	3	17	19	4	17	8		V_H5-10	V_K2D-29	+
EC2A	P1C2	41	22	46	100	87	100	94	95	79	102	100	97	100	99	96	83	7	20	13		V_H4-39	V_K3-15	+
EC2A	P1A1	64	35	49	97	96	97	98	96	92	98	97	96	98	97	96	94	92	1	6		V_H4-34	V_K3-20	-
EC2A	P2E3	15	10	14	88	87	99	100	86	70	96	86	97	87	95	76	42	9	11	6		V_H1-2	V_K3-11	+
EC2A	P1D6	9	25	27	87	92	99	99	79	70	97	78	98	79	87	62	45	8	16	7		V_H1-2	V_K3-15	-
EC2A	P1D3	47	18	13	92	100	100	96	92	92	84	99	97	97	95	95	87	74	7	4		V_H1-2	V_K3-11	+
EC2A	P2C2	43	33	20	100	87	100	100	95	100	99	100	96	100	98	100	96	100	15	0		V_H1-2	V_K3-11	-
EC2A	P1A2	26	23	28	99	97	98	98	90	63	99	98	98	98	95	85	50	36	13	3		V_H4-59	V_K3-11	+
EC2A	P1G4	1	0	2	84	98	99	93	87	78	87	95	99	100	97	79	55	47	0	0		V_H4-39	V_K1-5	-
EC2A	P1B3	12	16	11	91	94	100	97	92	86	81	99	100	100	93	77	76	62	10	9		V_H4-39	V_K3-15	+
EC2A	P1F5	6	6	0	88	83	100	98	86	77	92	99	100	100	94	72	41	41	0	6		V_H4-39	V_K3-15	+
EC2A	P1F1	12	12	12	86	94	100	100	85	80	96	100	100	100	100	72	65	45	6	15		V_H4-39	V_K1-5	+
EC2A	P1B2	5	1	21	72	82	88	100	100	80	96	91	100	100	94	100	56	75	27	10		V_H4-39	V_K1-5	+
EC2A	P2E4	0	0	1	100	81	100	89	100	95	101	100	100	100	100	98	95	100	0	12		V_H4-39	V_K1-5	-
EC2A	P2B6	0	0	0	96	97	97	96	97	94	97	96	97	91	96	96	96	95	0	0		V_H4-39	V_K1-5	+
EC4A	P2C1	3	10	6	28	5	8	9	6	10	8	12	11	13	14	14	16	12	98	98		V_H4-39	V_K1-5	+
EC4B	P1E3	0	0	0	0	0	12	7	0	0	2	11	9	13	15	39	0	5	20	92		V_H3-33	V_K1-16	-
	Domain	1	1	1	2	2	2	2	2	2	2	2	2	2	2	2	2		4	4				

Percent Inhibition
70-100%
50-69%
40-49%
<40%

Figure S7. Related to Figure 5. Similar sterically-distinct epitopes are detected at pre-onset and diagnosis time points. A flow-based blocking assay was used to detect sterically-distinct epitopes of antibodies from patient ISD068 derived from (A) pre-onset and (B) diagnosis time points. Similar frequencies of EC1A and EC2A epitopes could be detected at pre-diagnosis, as well as diagnosis time point, suggesting that no epitope spreading has occurred between the two time points. Designated name of the epitope bound by antibodies is specified on the left of the chart where appropriate. Domain-specificity of mAbs derived from the diagnosis time point is illustrated at the bottom of the chart (Orange: EC1; Grey: EC2; Gold: EC4; White: Interdomain). mAbs in the same clonal family are represented on the right of the chart. VH and VL usage and pathogenicity are listed on the right of the panel. Pathogenicity was determined by using the following cut-off values of the dissociation index number: - = DI≤10; + = 10<DI≤100.

TABLE S1. Related to Figure 1. Characteristics of subjects at time of enrollment

GROUP	Patient ID	Gender	Age	Disease	% B cells of lymphocytes	Medications (mg/d)	Mo. Post recent Rtx	No. of previous Rtx	PDAI[a]	Dsg3 Titer[c]	Dsg1 titer[d]
PV patients DIAGNOSIS (n=17)	ISD001	M	48	mucocutaneous	10.9	Prednisone (30)			12	20	133
	ISD003	M	26	mucosal	8.2	Prednisone (20)			11	137	neg
	ISD020	M	48	mucocutaneous	6.3	Prednisone (60)			15	69	169
	ISD058	F	43	mucosal	15.8	Prednisone (30)			15	50	neg
	ISD061	F	54	mucosal	8.8	Prednisone (60)			6	191	neg
	ISD066	M	52	mucocutaneous	1.8	Prednisone (30)			36	181	199
	ISD068[f]	***M***	***60***	***mucocutaneous***	***4.3***	***Prednisone (40)***			***8***	***195***	***neg***
	ISD069	M	38	mucocutaneous	13.7	Prednisone (30)			55	204	neg
	ISD070	M	48	mucocutaneous	11.8	None			0	200	117
	ISD082	M	54	mucocutaneous	9.4	Prednisone (80)			5	49	38
	ISD086	F	31	mucocutaneous	10.0	Prednisone (60)			4	128	neg
	ISD091	F	45	mucocutaneous	5.7	Prednisone (60)			64	153	171
	ISD100	M	60	mucocutaneous	10.1	Prednisone (20)			20	169	neg
	ISD102[f]	***F***	***45***	***mucocutaneous***	***6.5***	***Prednisone (10)***			***36***	***200***	***neg***
	ISD106	F	51	mucocutaneous	11.5	Prednisone (20)			4	75	neg
	ISD109	F	48	mucocutaneous	6.8	Prednisone (80)			20	166	38
	ISD112	M	42	mucocutaneous	7.5	Data unavailable			30[b]	151	164
PV patients REMISSION[e] (post-Rtx) (n=10)	R15	F	45	mucosal	10.8	None	20	0	1	neg	neg
	R15-06	M	41	mucosal	8.8	Cellcept (500)	19	2	0	120	neg
	ISD002	F	48	mucosal	2.3	None	6	1	0	neg	neg
	ISD025	M	58	mucosal	7.8	Prednisone (2.5)	22	0	0	neg	neg
	ISD060	F	47	mucosal	13.2	None	17	0	2	24	neg
	ISD068	M	63	mucocutaneous	3.2	None	11	2	0	29	neg
	ISD069	M	39	mucocutaneous	7.6	None	13	0	3	neg	neg
	ISD070	M	51	mucocutaneous	8.2	None	29	0	1	135	neg
	ISD072	F	51	mucocutaneous	8.9	None	12	0	0	neg	neg
	ISD082	M	55	mucocutaneous	5.5	None	13	0	0	neg	neg
PV patients RELAPSE[e]	ISD005	M	64	mucocutaneous	3.2	Prednisone (40)	77	0	12	131	98
	ISD031	M	77	mucocutaneous	5.4	None	27	1	1	neg	neg
	ISD038	M	66	mucocutaneous	5.6	None	13	0	15	112	175

PV patients RELAPSE[e] (cont'd) (post-Rtx) (n=17)	ISD060	F	48	mucosal	11.9	None	22	0	5	43	neg
	ISD061	F	55	mucosal	5.5	None	11	0	0	34	neg
	ISD063	F	61	mucocutaneous	10.6	None	8	0	12	149	neg
	ISD068	M	62	mucocutaneous	3.1	None	13	1	2	73	neg
	ISD069	M	41	mucocutaneous	16	None	20	1	2	45	neg
	ISD070	M	51	mucocutaneous	7.1	None	36	0	7	130	neg
	ISD072	F	52	mucocutaneous	13.1	None	21	1	0	108	187
	ISD074	M	49	mucocutaneous	9.6	None	12	3	15	62	94
	ISD084	F	50	mucocutaneous	4.3	None	9	0	3	neg	neg
	ISD086	F	32	mucocutaneous	9.3	None	17	0	3	159	67
	ISD104	F	50	mucosal	37.2	None	10	0	22	57	neg
	ISD106	F	51	mucocutaneous	7.33	None	8	0	4	47	neg
	ISD110	M	37	mucocutaneous	14.4	None	20	0	2	37	neg
	ISD122	M	63	mucocutaneous	3.7	None	45	4	25	78	neg
Healthy controls (n=11)	HC01	F	48		11.3						
	HC02	F	53		12.3						
	HC08	F	57		8.7						
	HC13	M	40		4.1						
	HC15	F	31		11.5						
	H15-08	F	45		4.9						
	H15-11	F	45		6.6						
	H15-13	F	46		14.9						
	HC421	F	25		5.6						
	HC435	F	27		9.5						
	HC467	M	37		10.0						

[a]Pemphigus Disease Activity Index (According to: Rosenbach M, et al. Reliability and convergent validity of two outcome instruments for pemphigus. *The Journal of investigative dermatology.* 2009;129(10):2404-10.

[b]PDAI score calculated from photos

[c]Dsg3 titers reported in U/mL. Cut-off values determined by manufacturer recommendation: neg (negative) < 19

[d]Dsg1 titers reported in U/mL. Cut-off values determined by manufacturer recommendation: neg (negative) < 18

[e]According to Rosenbach M, et al. Reliability and convergent validity of two outcome instruments for pemphigus. *The Journal of investigative dermatology.* 2009;129(10):2404-10.

[f]Patient ISD068 and ISD102 are highlighted as patients of interest; Dsg3-specific mAbs are derived and characterized from these two patients.

TABLE S2. Related to Figure 2. Repertoire analysis of Dsg3-specific mAbs isolated from two PV patients.

Patient	mAb[a]	Isotype	Heavy Chain		CDR3	# Mutations	Light Chain			CDR3	# Mutations
			V gene	J gene	length (nt)	(R/S ratio)	K/L	V gene	J gene	length (nt)	(R/S ratio)
ISD102 Diagnosis (n=20)	P3F3	IgG1	V_H1-46	J_H3	54	23 (19/4)	Kappa	V_κ2-24	J_κ5	27	8 (7/1)
	P5D5	IgG1	V_H1-46	J_H2	27	25 (20/5)	Kappa	V_κ2-24	J_κ2	27	14 (10/4)
	P5F5[+]	IgA1	V_H3-15	J_H4	54	18 (11/7)	Lambda	V_L6-57	J_L3	30	18 (15/3)
	P5F4[+]	IgA1	V_H3-15	J_H4	54	22 (14/8)	Lambda	V_L6-57	J_L3	30	19 (16/3)
	P5B3[~]	IgG1	V_H3-15	J_H4	54	20 (13/7)	Lambda	V_L3-10	J_L3	33	17 (12/5)
	P5C3[~]	IgG1	V_H3-15	J_H4	54	26 (15/11)	Lambda	V_L3-10	J_L3	33	20 (15/5)
	P5A6[δ]	IgG1	V_H1-46	J_H3	45	14 (9/5)	Kappa	V_κ2-24	J_κ5	27	13 (8/5)
	P5G3[δ]	IgG1	V_H1-46	J_H3	45	18 (13/5)	Kappa	V_κ2-24	J_κ5	27	12 (9/3)
	P5B1	IgG1	V_H1-46	J_H6	54	24 (14/10)	Kappa	V_κ2-24	J_κ1	27	7 (4/3)
	P4D4[β]	IgG1	V_H3-23	J_H1	57	19 (14/5)	Lambda	V_L3-21	J_L3	33	21 (15/7)
	P5G4[β]	IgG1	V_H3-23	J_H1	57	15 (10/5)	Lambda	V_L3-21	J_L3	33	16 (15/1)
	P5D1[β]	IgG1	V_H3-23	J_H1	57	17 (11/6)	Lambda	V_L3-21	J_L3	33	21 (17/4)
	P5A2	IgG1	V_H3-53	J_H4	45	6 (4/2)	Lambda	V_L1-40	J_L3	33	12 (10/2)
	P5G6	IgG1	V_H1-2	J_H4	42	27 (20/7)	Lambda	V_L3-21	J_L2	36	12 (11/1)
	P5E4[Ω]	IgG1	V_H4-61	J_H4	36	19 (11/8)	Lambda	V_L6-57	J_L3	30	14 (11/3)
	P5B6[Ω]	IgG1	V_H4-61	J_H4	36	15 (9/6)	Lambda	V_L6-57	J_L3	30	12 (9/3)
	P5D4	IgG1	V_H3-66	J_H4	33	18 (17/1)	Kappa	V_κ1-5	J_κ2	27	5 (4/1)
	P3A6	IgG1	V_H1-69	J_H3	54	30 (23/7)	Kappa	V_κ3-15	J_κ2	30	11 (9/2)
	P5C2	IgG1	V_H4-4	J_H4	42	17 (11/6)	Kappa	V_κ3-11	J_κ4	27	2 (2/0)
	P5A4	IgM	V_H1-2	J_H4	57	0	Kappa	V_κ3-20	J_κ1	27	0
ISD068 Diagnosis (n=25)	P1E6[*]	IgG4	V_H5-10	J_H4	48	18 (14/4)	Kappa	V_κ2D-29	J_κ4	27	14 (9/5)
	P1C3[*]	IgG4	V_H5-10	J_H4	48	24 (21/3)	Kappa	V_κ2D-29	J_κ4	27	19 (9/10)
	P1D2[±]	IgG1	V_H1-58	J_H3	33	24 (20/4)	Kappa	V_κ3-11	J_κ2	30	12 (9/3)
	P1A1	IgG4	V_H4-34	J_H4	69	27 (25/12)	Kappa	V_κ3-20	J_κ2	27	13 (11/2)
	P1C5	IgG1	V_H4-59	J_H4	60	39 (31/8)	Kappa	V_κ3-15	J_κ1	30	11 (8/3)
	P1C6	IgG1	V_H3-23	J_H3	63	26 (19/7)	Kappa	V_κ4-1	J_κ5	27	16 (10/6)
	P1F1	IgG1	V_H4-39	J_H2	42	29 (21/8)	Kappa	V_κ1-5	J_κ1	27	26 (19/7)
	P1B3[#]	IgG4	V_H4-39	J_H2	84	43 (28/15)	Kappa	V_κ3-15	J_κ2	33	18 (10/8)
	P1F5[#]	IgG1	V_H4-39	J_H2	84	29 (17/12)	Kappa	V_κ3-15	J_κ2	33	20 (16/4)
	P1D6[†]	IgG4	V_H1-2	J_H1	30	22 (14/8)	Kappa	V_κ3-15	J_κ1	33	16 (12/4)
	P2E3	IgG4	V_H1-2	J_H4	30	20 (15/5)	Kappa	V_κ3-11	J_κ2	33	15 (11/4)
	P1G4	IgG1	V_H4-39	J_H2	42	30 (21/9)	Kappa	V_κ1-5	J_κ1	27	20 (14/6)
	P1D3[^]	IgG1	V_H1-2	J_H4	72	29 (24/5)	Kappa	V_κ3-11	J_κ2	33	16 (11/5)
	P2C2[^]	IgG1	V_H1-2	J_H4	72	28 (24/4)	Kappa	V_κ3-11	J_κ2	33	21 (17/4)
	P1B2[%]	IgG1	V_H4-39	J_H2	63	21 (16/5)	Kappa	V_κ1-5	J_κ1	27	20 (14/6)

	P2E4[%]	IgG1	V_H4-39	J_H2	63	21 (15/6)	Kappa	V_K1-5	J_K1	27	20 (12/8)
	P1C2	IgG4	V_H4-39	J_H5	39	30 (19/11)	Kappa	V_K3-15	J_K2	33	15 (13/2)
	P1A2[‡]	IgG1	V_H4-59	J_H2	48	29 (20/9)	Kappa	V_K3-11	J_K2	18	15 (11/4)
	P2C1	IgG1	V_H4-39	J_H4	48	27 (17/10)	Kappa	V_K1-5	J_K1	21	13 (9/4)
	P2B6	IgG1	V_H4-39	J_H4	42	27 (19/8)	Kappa	V_K1-5	J_K1	27	20 (14/6)
	P1E4[&]	IgG1	V_H4-4	J_H5	45	28 (23/5)	Kappa	V_K3-15	J_K3	30	15 (9/6)
	P1B5[&]	IgG1	V_H4-4	J_H5	45	36 (28/8)	Kappa	V_K3-15	J_K3	30	12 (7/5)
	P1A5	IgG1	V_H3-23	J_H4	39	0	Lambda	V_L1-47	J_L2	33	0
	P1G1	IgA1	V_H3-74	J_H3	51	19 (14/5)	Kappa	V_K3-15	J_K3	27	11 (10/1)
	P1E3	IgG4	V_H3-33	J_H3	60	7 (5/2)	Kappa	V_K1-16	J_K4	27	6 (3/3)
ISD068 Pre-Diagnosis (n=25)	P1D4	IgG4	V_H1-18	J_H4	42	16 (11/5)	Kappa	V_K1-9	J_K4	27	8 (7/1)
	P2F2[†]	IgG4	V_H1-2	J_H1	30	25 (18/7)	Kappa	V_K3-15	J_K1	33	17 (13/4)
	P1D5	IgG4	V_H1-2	J_H3	48	23 (14/9)	Lambda	V_L1-51	J_L3	36	8 (5/3)
	P1D6	IgG1	V_H1-2	J_H3	27	31 (18/13)	Kappa	V_K3-11	J_K5	33	13 (8/5)
	P1B2	IgG1	V_H1-2	J_H4	42	36 (25/11)	Lambda	V_L1-47	J_L1	33	21 (10/11)
	P1B6	IgG4	V_H1-2	J_H4	24	16 (10/6)	Kappa	V_K3-20	J_K4	27	12 (10/2)
	P2D1[^]	IgG1	V_H1-2	J_H4	39	22 (17/5)	Kappa	V_K3-15	J_K4	33	15 (12/3)
	P1E3[^]	IgG4	V_H1-2	J_H4	39	17 (15/2)	Kappa	V_K3-15	J_K4	33	12 (10/2)
	P1G5[^]	IgG4	V_H1-2	J_H4	39	24 (14/10)	Kappa	V_K3-15	J_K4	33	12 (10/2)
	P2D6[±]	IgG4	V_H1-58	J_H3	33	10 (9/1)	Kappa	V_K3-11	J_K2	30	8 (6/2)
	P1D1	IgG1	V_H1-69	J_H5	54	28 (22/6)	Kappa	V_K3-11	J_K4	30	5 (4/1)
	P1C4	IgM	V_H3-23	J_H1	54	3 (1/2)	Lambda	V_L3-25	J_L2	33	10 (7/3)
	P2F4	IgG4	V_H3-53	J_H6	42	31 (26/5)	Kappa	V_K1-5	J_K1	27	15 (10/5)
	P1G1[§]	IgG1	V_H4-4	J_H2	72	35 (26/9)	Kappa	V_K3-20	J_K4	30	18 (14/4)
	P1G2[§]	IgG1	V_H4-4	J_H2	72	36 (27/9)	Kappa	V_K3-20	J_K4	30	14 (12/2)
	P2D2[§]	IgG1	V_H4-4	J_H2	72	38 (28/10)	Kappa	V_K3-20	J_K4	30	16 (13/3)
	P2D3[%]	IgA1	V_H4-39	J_H2	63	15 (11/4)	Kappa	V_K1-5	J_K1	27	11 (10/1)
	P2G2[α]	IgG1	V_H4-39	J_H2	42	21 (11/10)	Kappa	V_K1-5	J_K1	27	17 (11/6)
	P1F3[α]	IgG1	V_H4-39	J_H2	42	20 (14/6)	Kappa	V_K1-5	J_K1	27	15 (10/5)
	P2G1[#]	IgG1	V_H4-39	J_H2	84	23 (15/8)	Kappa	V_K3-15	J_K2	33	14 (12/2)
	P2C4	IgG1	V_H4-59	J_H4	60	34 (28/6)	Kappa	V_K3-15	J_K1	30	9 (6/3)
	P1D2[‡]	IgG1	V_H4-59	J_H2	48	27 (20/7)	Kappa	V_K3-11	J_K2	18	16 (11/5)
	P1C5*	IgG4	V_H5-10	J_H4	48	14 (10/4)	Kappa	V_K2D-29	J_K4	27	11 (5/6)
	P1G3	IgG1	V_H5-10	J_H3	51	21 (18/3)	Kappa	V_K1-33	J_K2	27	19 (15/4)
	P1F4	IgG4	V_H5-51	J_H5	75	23 (20/3)	Lambda	V_L3-9	J_L3	30	18 (9/9)

[a] Matching symbols indicate mAbs that are part of the same clonal expansion.

TABLE S3. Related to Figure 6. Characteristics of antibodies selected for germline reversion.

Patient	mAb	Isotype	Heavy Chain		CDR3 length (nt)	R/S Ratio			# unique clones	Epitope	Pathogenicity[a]
			V gene	J gene		VH	FR	CDR			
ISD068 (n=10)	P1C3	IgG4	VH5-10	JH4	48	7	4	9	2	EC1A	+
		Kappa	VK2D-29	JK4	27						
	P1D2	IgG1	VH1-58	JH3	33	5	4.33	6	2	EC1A	+
		Kappa	VK3-11	JK2	30						
	P1A1	IgG4	VH4-34	JH4	69	1.25	1.56	1.67	1	EC2A	-
		Kappa	VK3-20	JK2	27						
	P1B2	IgG1	VH4-39	JH2	42	3.2	3	5	3	EC2A	+
		Kappa	VK1-5	JK1	27						
	P1B3	IgG4	VH4-39	JH2	84	1.87	1.1	6.5	2	EC2A	+
		Kappa	VK3-14	JK2	33						
	P1D6	IgG4	VH1-2	JH1	30	1.75	1.67	2	1	EC2A	-
		Kappa	VK3-15	JK1	33						
	P1F1	IgG1	VH4-39	JH2	42	2.63	2.71	4	3	EC2A	+
		Kappa	VK1-5	JK1	27						
	P2E3	IgG4	VH1-2	JH4	30	3	2.25	6	1	EC2A	+
		Kappa	VK3-11	JK2	33						
	P2C1	IgG1	VH4-39	JH4	48	1.7	0.55	9	1	EC4A	+
		Kappa	VK1-5	JK1	21						
	P1E3	IgG4	VH3-33	JH3	60	2.5	4	1	1	EC4B	-
		Kappa	VK1-16	JK4	27						
ISD102 (n=10)	P3F3	IgG1	VH1-46	JH3	54	4.75	2.75	7	1	EC1A	++
		Kappa	VK2-24	JK5	27						
	P5A2	IgG1	VH3-53	JH4	45	2	2	2	2	EC1A	-
		Lambda	VL1-40	JL3	33						
	P5B1	IgG1	VH1-46	JH6	54	1.86	1.57	1	1	EC1A	++
		Kappa	VK2-24	JK4	27						
	P5G3	IgG1	VH1-46	JH3	45	2.60	2.75	6	2	EC1A	++
		Kappa	VK2-24	JK5	27						
	P5G6	IgG1	VH1-2	JH4	42	2.86	1.25	8	1	EC1 domain	-
		Lambda	VL3-21	JL2	36						
	P5B3	IgG1	VH3-15	JH4	54	1.86	0.71	6	3	EC1B	+
		Lambda	VL3-10	JL3	33						
	P5F5	IgA1	VH3-15	JH4	54	1.57	1.17	4	3	EC1B	+
		Lambda	VL6-57	JL3	30						
	P3A6	IgG1	VH1-69	JH3	54	3.29	2.67	7	1	EC4B	-
		Kappa	VK3-15	JK2	30						
	P5D4	IgG1	VH3-66	JH4	33	17	12	5	2	EC4B	+
		Kappa	VK1-5	JK2	27						
	P5E4	IgG1	VH4-61	JH4	36	1.38	1.14	1.5	2	EC4B	-
		Lambda	VL6-57	JL3	30						

[a] Pathogenicity was determined using the following cut-off values for the dissociation index number: - = DI≤10; + = DI>10; ++ = DI>100.
