# Supplementary material for: Single-Cell Analysis Suggests that Ongoing Affinity Maturation Drives the Emergence of Pemphigus Vulgaris Autoimmune Disease
Source: Cell Rep. Author manuscript; Available in PMC 2019 Aug 6. (PMC6684256; doi:10.1016/j.celrep.2019.06.066)
Supplement: 2 [file NIHMS1535565-supplement-2.pdf]

Article

Cell Reports

Single-Cell Analysis Suggests that Ongoing Affinity Maturation Drives the Emergence of Pemphigus Vulgaris Autoimmune Disease

Graphical Abstract

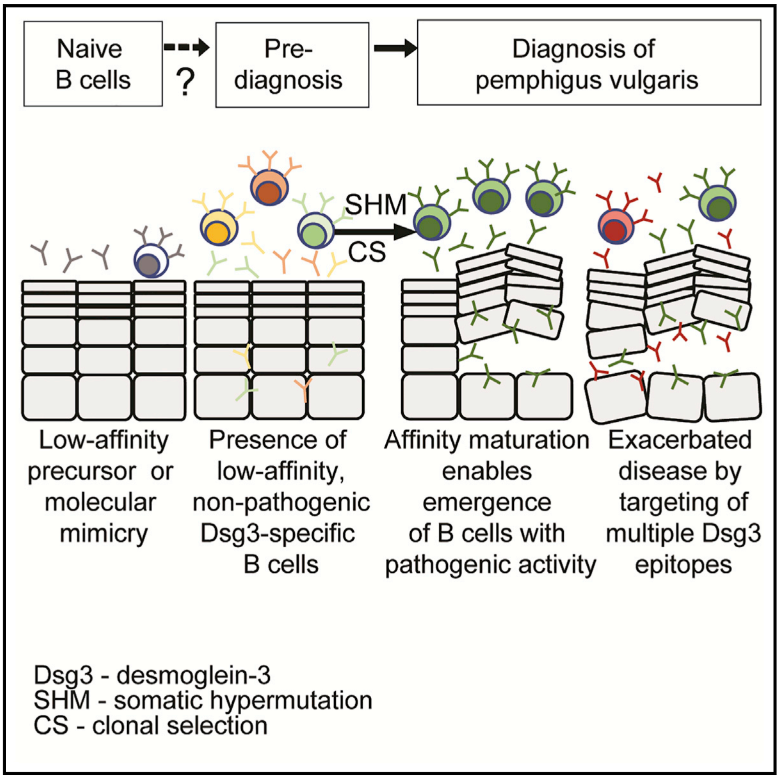

Highlights

- Single-cell analysis of autoantigen-specific memory B cells in pemphigus vulgaris
- Memory B cells undergo extensive affinity maturation and acquire pathogenic activity
- Memory B cell-derived autoantibodies are limited in epitope specificity and repertoire
- Multiple antibodies can work synergistically to enhance pathogenic activity

Authors

Alice Cho, Amber L. Caldara,
Nina A. Ran, ..., Ron Feldman,
Andrew P. Kowalczyk, Jens Wrammert

Correspondence

jwramme@emory.edu

In Brief

Cho et al. use single-cell sorting methods to detect and characterize autoantigen-specific memory B cells before and during the development of pemphigus vulgaris disease. They find that memory B cells undergo ongoing affinity maturation to generate a limited repertoire or pathogenic antibodies, which work synergistically to enhance overall pathogenic activity.

Cho et al., 2019, Cell Reports *28*, 909–922
July 23, 2019 © 2019 The Authors.
https://doi.org/10.1016/j.celrep.2019.06.066

Cell Reports
Article

OPEN ACCESS
CellPress

Single-Cell Analysis Suggests that Ongoing Affinity Maturation Drives the Emergence of Pemphigus Vulgaris Autoimmune Disease

Alice Cho,[1,2] Amber L. Caldara,[3,4] Nina A. Ran,[5] Zach Menne,[1,2] Robert C. Kauffman,[1,2] Maurizio Affer,[1,2] Alexandra Llovet,[1,2] Carson Norwood,[1,2] Aaron Scanlan,[1,2] Grace Mantus,[1,2] Bridget Bradley,[4] Stephanie Zimmer,[3,4] Thomas Schmidt,[6] Michael Hertl,[6] Aimee S. Payne,[5] Ron Feldman,[4] Andrew P. Kowalczyk,[3,4] and Jens Wrammert[1,2,7,*]

[1]Department of Pediatrics, Division of Infectious Disease, Emory University School of Medicine, Atlanta, GA, USA
[2]Emory Vaccine Center, Emory University School of Medicine, Atlanta, GA, USA
[3]Department of Cell Biology, Emory University, Atlanta, GA, USA
[4]Department of Dermatology, Emory University School of Medicine, Atlanta, GA, USA
[5]Department of Dermatology, Perelman School of Medicine, University of Pennsylvania, Philadelphia, PA, USA
[6]Department of Dermatology and Allergology, Philipps-University, Marburg, Germany
[7]Lead Contact
*Correspondence: jwramme@emory.edu
https://doi.org/10.1016/j.celrep.2019.06.066

SUMMARY

Pemphigus vulgaris (PV) is an autoimmune disease characterized by blistering sores on skin and mucosal membranes, caused by autoantibodies primarily targeting the cellular adhesion protein, desmoglein-3 (Dsg3). To better understand how Dsg3-specific autoantibodies develop and cause disease in humans, we performed a cross-sectional study of PV patients before and after treatment to track relevant cellular responses underlying disease pathogenesis, and we provide an in-depth analysis of two patients by generating a panel of mAbs from single Dsg3-specific memory B cells (MBCs). Additionally, we analyzed a paired sample from one patient collected 15-months prior to disease diagnosis. We find that Dsg3-specific MBCs have an activated phenotype and show signs of ongoing affinity maturation and clonal selection. Monoclonal antibodies (mAbs) with pathogenic activity primarily target epitopes in the extracellular domains EC1 and EC2 of Dsg3, though they can also bind to the EC4 domain. Combining antibodies targeting different epitopes synergistically enhances *in vitro* pathogenicity.

INTRODUCTION

Pemphigus vulgaris (PV) is a human autoantibody-mediated disease (Anhalt et al., 1982; Beutner and Jordon, 1964; Mascaró et al., 1997) in which patients experience painful blistering sores on skin and mucosal membranes (Lever, 1953). The primary autoantigenic target of PV is the protein desmoglein-3 (Dsg3) (Amagai et al., 1994, 1992, 1991, 1996). Dsg3 is a transmembrane glycoprotein that mediates *trans* and *cis* adhesive interactions necessary for assembly of the desmosome (Amagai et al., 1991; Boggon et al., 2002; Harrison et al., 2016; Wu et al., 2010), which is a cell-to-cell adhesive structure found in epidermal keratinocytes (Delva et al., 2009; Kowalczyk and Green, 2013). Targeting Dsg3 has been shown to be both necessary and sufficient to cause disease using *in vitro* and murine *in vivo* models of PV (Amagai et al., 1994, 1996; Ishii et al., 2005; Koch et al., 1997). While PV patients with disease limited to mucosal tissues have autoantibodies directed solely toward Dsg3, patients with mucocutaneous PV can also have autoantibodies targeting the homologous protein Dsg1 (Ding et al., 1997).

Unlike many autoimmune diseases that have poorly characterized or multiple autoantigenic targets (Robert-Pachot et al., 2007; Sherer et al., 2004), the identification of a single, well-defined autoantigen makes PV a unique human disease to study B cell-mediated autoimmunity at an antigen-specific level. While B cell-derived autoantibodies clearly drive PV pathogenesis (Amagai et al., 1994, 1992, 1991, 2000; Anhalt et al., 1982), it remains unclear which subset of B cells contributes to serum autoantibody responses or where these cells may reside (Colliou et al., 2013; Nishifuji et al., 2000; Yuan et al., 2017; Chen et al., 2017). Studies of other organ-specific autoimmune diseases suggest that memory B cells (MBCs) found in circulation play a crucial role in autoimmunity (Maurer et al., 2012; Muto et al., 2017).

Dsg3-specific monoclonal antibodies (mAbs) from PV patients have been studied using antibody phage display (APD) (Payne et al., 2005) and generation of hybridomas from MBCs (Di Zenzo et al., 2012; Qian et al., 2007; Yeh et al., 2006). These studies have been informative for our understanding of the functional qualities of Dsg3-specific mAbs. However, while APD is a powerful tool to screen large numbers of cells, it artificially pairs heavy and light chains that may not represent the cognate pairings of the repertoire present *in vivo* (Hammers and Stanley, 2014). In contrast, hybridomas generated from MBCs do retain the natural heavy and light chain pairings, but they are labor intensive and often result in smaller numbers of antigen-specific mAbs to be analyzed (Corti and Lanzavecchia, 2014). Overall, these studies have isolated Dsg3-specific mAbs with varying levels of repertoire diversity and were found to have a mix of "pathogenic"

This is an open access article under the CC BY-NC-ND license (http://creativecommons.org/licenses/by-nc-nd/4.0/).

and "non-pathogenic" activity (Di Zenzo et al., 2012; Payne et al., 2005), leading to ongoing questions about how antibodies cause pathology. Current models suggest that pathogenic autoantibodies target epitopes crucial for Dsg3-mediated desmosomal adhesion and mainly act by steric hinderance (Di Zenzo et al., 2012; Amagai et al., 1992), though they may also engage signaling pathways (Mao et al., 2011) or trigger endocytosis and degradation of Dsg3 (Calkins et al., 2006; Saito et al., 2012; Stahley et al., 2014, 2016). While it remains unclear how Dsg3-specific mAbs develop, evidence from other autoimmune diseases such as lupus suggest that autoantibodies develop prior to disease onset, and accumulation of autoantibodies ultimately drive disease pathogenesis (Arbuckle et al., 2003). Thus, it is important to study mAbs in order to understand how they develop and cause autoimmunity, ultimately guiding efforts to develop effective therapies to treat PV.

Herein, we determine the dynamics of Dsg3-specific B cell responses in PV patients at the time of diagnosis or after treatment with B cell depletive therapy. We show that the detection of activated Dsg3-specific MBCs correlates with the presentation of disease symptoms, while circulating Dsg3-specific plasmablasts remained undetectable in all samples analyzed. We derived and characterized a large panel of mAbs from single Dsg3-specific MBCs sorted from two PV patients at the time of diagnosis. We also had access to a unique paired clinical sample collected 15 months prior to disease onset from one of the donors, which allowed us to show that autoreactive cells were present prior to disease onset. Our study suggests that MBCs play an important role in PV pathogenesis and that ongoing affinity maturation and clonal restriction drive the development of pathogenic mAbs that bind a limited number of epitopes on Dsg3. We also found that the onset of disease symptoms correlates with an increase in the frequency of antibodies with pathogenic activity, and multiple pathogenic antibodies targeting distinct epitopes act in synergy to amplify the overall pathogenic potency.

RESULTS

Activated Dsg3-Specific MBCs Are Found in PV Patients Presenting with Active Disease

In order to understand the dynamics of autoreactive B cell responses in PV patients, we performed a cross-sectional analysis of PV patients at the time of diagnosis, or at remission or relapse after treatment with the B cell-depletive therapy Rituximab (Rtx) (Joly et al., 2017). Overall, 32 patients were enrolled, with 9 patients followed longitudinally throughout treatment, resulting in a total of 54 samples analyzed. Because reconstitution of the B cell compartment typically begins 6–9 months post-therapy (Breedveld et al., 2007), we analyzed samples only from remission and relapse patients who were ≥6 months post-Rtx treatment (median 13 or 15 months, respectively) with comparable frequencies of B cells (median 8% of lymphocytes) to PV patients at diagnosis (median 8.5%) and healthy controls (median 9.5%) (Table S1).

Anti-Dsg3 serum antibodies were detected in 95% (16/17) of the PV patients at the time of diagnosis but were absent in all 11 healthy controls (Figure 1A). The one patient with low titers had previous clinical data reporting titers reaching 173 U/mL, suggesting that the low titer at this particular time point is likely driven by concomitant medications. Anti-Dsg3 titers dropped below the limit of detection after Rtx therapy in 80% (8/10) of the remission patients. In contrast to most patients who respond to Rtx with a decrease in anti-Dsg3 serum titers (Figure S1A), two remission patients maintained high titers against Dsg3 despite efficient depletion of the B cell compartment (Figure S1B). 88% (15/17) of relapsing patients had detectable serum anti-Dsg3 titers. Anti-Dsg1 serum titers were only detected in 41% (7/17) of PV patients at diagnosis and 29% (5/17) at relapse, but they were absent from all patients at remission (Figure S1C). The presence of anti-Dsg1 titers was limited to mucocutaneous PV patients, although 43% (6/14) of these patients had serum titers against only Dsg3 (Table S1), similar to previous reports (Culton et al., 2015; Ding et al., 1997; Harman et al., 2001). Unlike serum autoantibody titers, which decrease after Rtx treatment (Figure S1D), protective titers against tetanus remained unaffected (Figure S1E).

We also determined if Dsg3-specific MBCs could be detected in these patients using an enzyme-linked immune absorbent spot (ELISPOT)-based assay (Pinna et al., 2009) (Figure 1B). Similar to the serology data, we detected Dsg3-specific MBCs in 94% (16/17) of PV patients at the time of diagnosis and 53% (9/17) of the relapse patients, whereas these cells were absent in healthy controls and present in only 20% (2/10) of remission patients (Figure 1C). The frequency of Dsg3-specific MBCs correlates positively with levels of anti-Dsg3 serum titers (Figure S1F), suggesting that peripheral MBCs directly replenish short-lived antibody-secreting cells responsible for serum autoantibodies. It remains unclear if the presence of Dsg3-specific MBCs in relapsing patients are a result of newly generated MBCs, or of the expansion of MBCs that survived Rtx-mediated depletion (Cho et al., 2017; Hammers et al., 2015). Overall, the presence of Dsg3-specific MBCs in diagnosis patients and the re-emergence of these cells during disease relapse confirm the importance of these cells in PV pathogenesis.

By focusing on the two patients with the highest frequencies of Dsg3-specific MBCs (Figure 1C), we detected Dsg3-specific MBCs by flow cytometry at a 100-fold-higher frequency than background staining in healthy controls (Figure 1D). Dsg3-specific cells expressed the classical MBC marker CD27 (Figure 1E), excluding these cells from the atypical IgD-CD27− MBCs described in other autoimmune diseases (Claes et al., 2016; Jenks et al., 2018; Saadoun et al., 2013). Interestingly, Dsg3-specific MBCs expressed the activation marker CD71, which was recently shown to be transiently expressed on newly generated MBCs in healthy influenza vaccinees (Ellebedy et al., 2016). Similar to that study, we found that in healthy controls, recently activated CD71+ hemagglutinin (HA)-specific MBCs were detected on day 7 post-influenza vaccination, whereas resting-specific MBCs found on day 28 post-vaccination were CD71 negative (Figure 1F). CD71 expression on Dsg3-specific MBCs suggests that these cells were recently activated and are part of ongoing immune responses, affirming their relevance to PV pathogenesis.

We also enumerated Dsg3-specific plasmablasts in peripheral blood of symptomatic PV patients (Figures S2A and S2B). Despite the presence of activated Dsg3-specific MBCs in

OPEN
ACCESS
CellPress

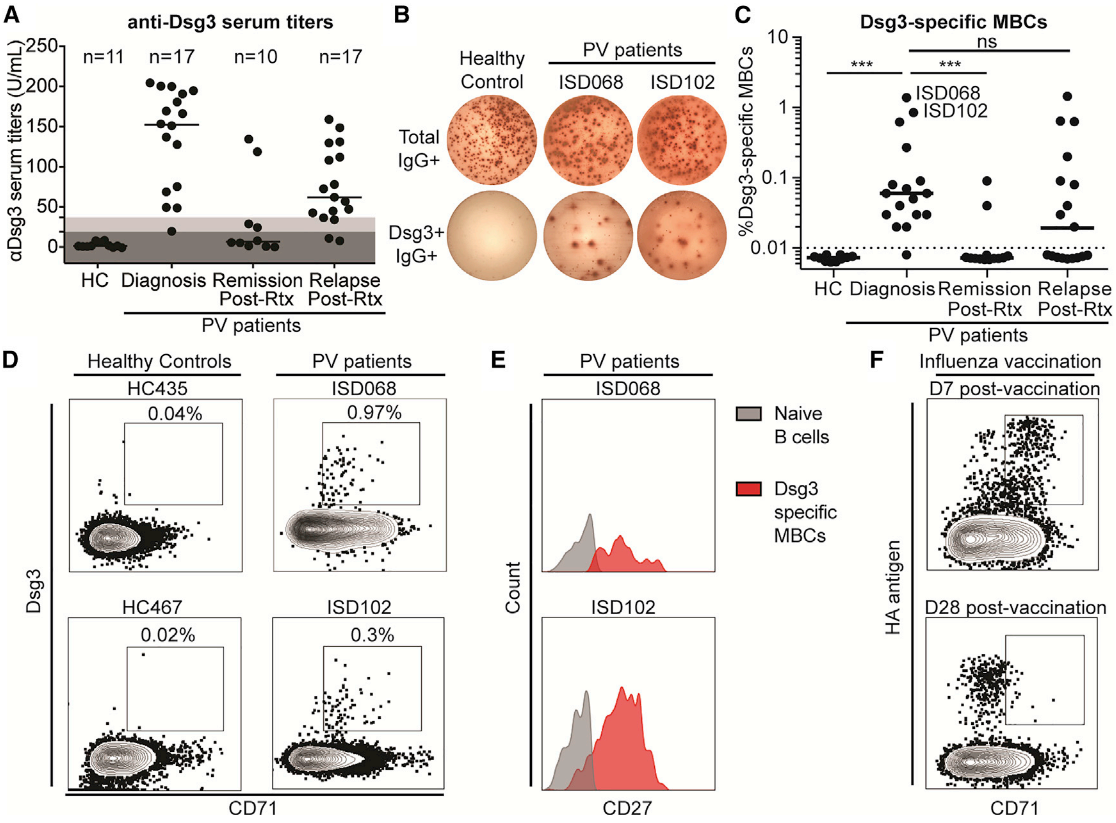

Figure 1. Activated Dsg3-Specific Memory B Cells Are Detected Exclusively in Patients with Active PV

(A) High levels of anti-Dsg3 serum antibody titers are present in PV patients at the time of diagnosis and during relapse following previous treatment with Rituximab (Rtx), but not in healthy controls and patients in remission. Cut-off values were determined based on manufacturer recommendations: dark gray = negative; light gray = indeterminate. Representative data of two individual experimental repeats are shown.

(B) Representative Dsg3-specific MBC assay.

(C) High frequencies of Dsg3+ MBCs were detected in PV patients at the time of diagnosis and after relapse but were largely undetectable in patients in remission and absent in healthy controls. Reported frequency of Dsg3-specific MBCs is the average of 3–5 replicates of a single experiment.

(D) Dsg3-specific MBCs (gated on CD3-CD19+IgD-CD20+ lymphocytes) are readily detected from two patients with high frequencies of Dsg3-specific MBCs (as shown in C).

(E) Dsg3-specific MBCs express the classic memory cell marker CD27.

(F) Dsg3-specific MBCs expressed the activation marker CD71, similar to activated HA-specific MBCs induced 7 days post-influenza vaccination. Steady-state MBCs found later after the vaccination are quiescent and do not express CD71. A one-way ANOVA was used to analyze these data. ***$p \leq 0.001$.

circulation, we were unable to detect any Dsg3-specific antibody-secreting plasmablasts (Figure S2C), similar to previous reports (Nishifuji et al., 2000). This finding contrasts extensive literature describing potent circulating plasmablast responses after vaccination or infection (Ellebedy et al., 2016; Kauffman et al., 2016; Tipton et al., 2015; Wrammert et al., 2012, 2008). This suggests that serum autoantibodies in PV patients are likely produced by locally differentiated plasmablasts found in tissues such as lymphoid organs or skin and/or mucosae (Yuan et al., 2017). Overall, the data confirm that Dsg3 is an important target in PV and that activated MBCs play an important role in disease pathogenesis.

Generation of High-Affinity Autoreactive MBCs via Extensive Affinity Maturation

We next performed an in-depth single-cell analysis of MBCs. Heavy and light immunoglobulin (Ig) rearrangements were PCR amplified from single Dsg3-specific MBCs sorted from patients ISD068 and ISD102 (Smith et al., 2009) and used for both repertoire analysis and generation of mAbs (Figure 2A). Overall, we analyzed 32 cells from each patient. The MBCs were largely class-switched, with the IgG1 isotype most prominently used (Figure 2B). We also detected several IgG4 sequences (Vidarsson et al., 2014), similar to previous reports that IgG1 and IgG4 isotypes are preferentially used by PV autoantibodies (Futei et al., 2001). A small number of Dsg3-specific MBCs used the IgA1 isotype, as previously described (Ellebrecht et al., 2018; Mentink et al., 2007; Spaeth et al., 2001). Similar isotype usage was observed in Dsg3-specific serum antibodies of these patients (Figure S3) as tested by ELISA using subclass-specific reagents confirmed to have no cross reactivity when tested against recombinant subclass-switched mAbs (data not shown).

We observed that about 50% of the analyzed sequences shared clonal origins with at least one other cell (Figure 2C).

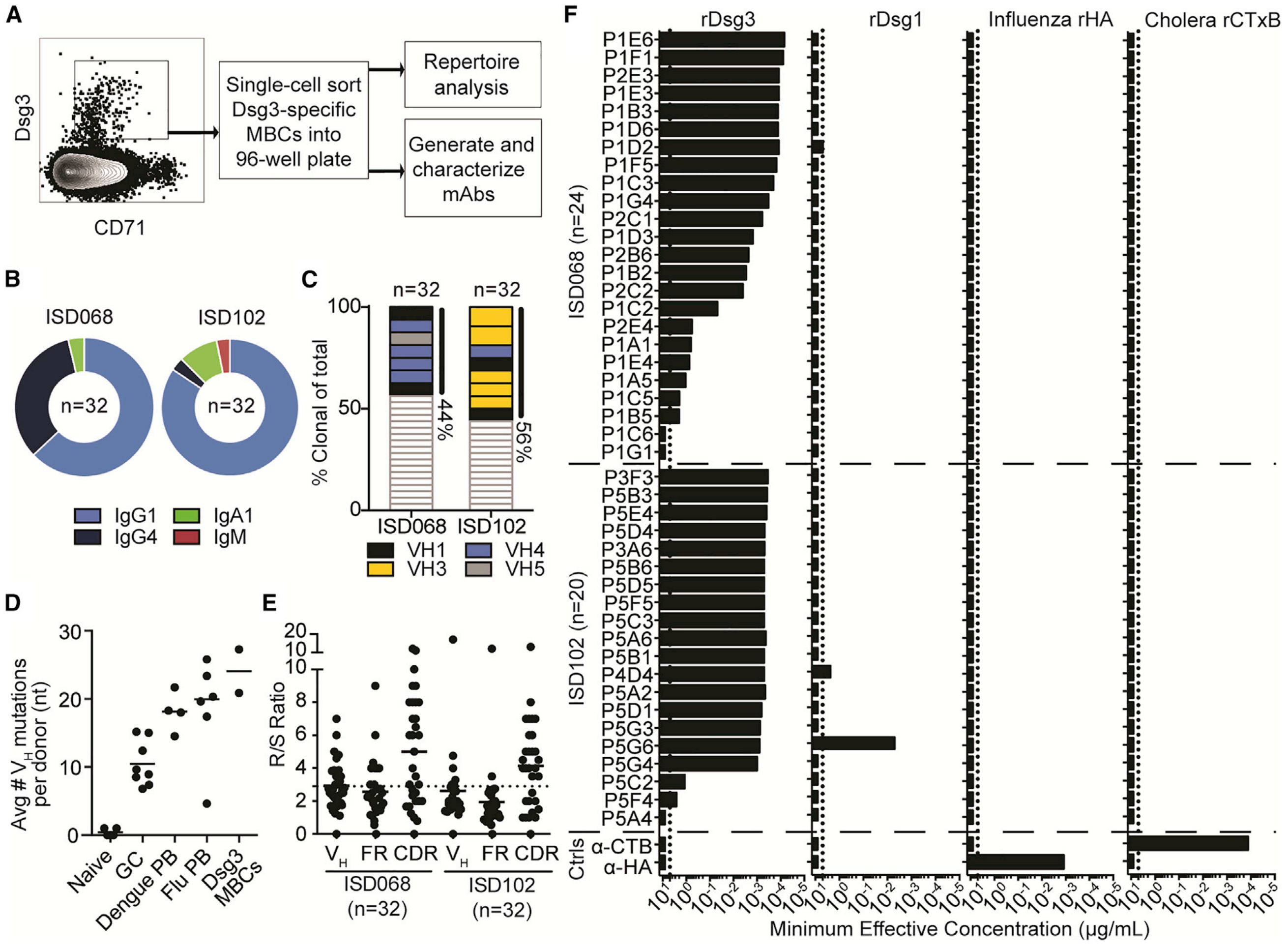

Figure 2. Dsg3-Specific MBCs Are Clonally Restricted, Show Signs of Extensive Antigenic Selection, and Are Highly Specific for Dsg3

(A) Dsg3-specific MBCs were single-cell sorted and used for repertoire analysis and mAb generation.

(B and C) Dsg3-specific MBCs were (B) largely class switched to IgG1 and (C) highly oligoclonal, with about 50% of sequences representing clonal expansions. Colors represent V_H gene usage of the clonal expansions.

(D) Dsg3+ MBCs display high frequencies of SHMs compared to previously analyzed B cell subsets (Pauli et al., 2014; Priyamvada et al., 2016; Wrammert et al., 2011). Each point represents the average number of mutations of all sequences analyzed from one individual donor.

(E) R/S ratio analysis of the entire V_H gene, FR, and CDR showed that Dsg3+ MBCs had an R/S ratio above 2.9 (dotted line) in the CDRs.

(F) Fourty-four mAbs were generated from the two patients. Most mAbs displayed specificity solely against Dsg3, with only two antibodies cross reactive with Dsg1. No mAbs bound irrelevant influenza HA (H1/California/2009) or cholera toxin B subunit antigens. The highest antibody concentration tested for this assay was 5 µg/mL (dotted line). Representative data of two individual experimental repeats are shown.

This is higher than the average frequency of clonal expansions detected in infection-induced plasmablasts, which is typically around 20% in influenza-, dengue-, and cholera-infected patients (Kauffman et al., 2016; Priyamvada et al., 2016; Wrammert et al., 2011). When looking at the V_H gene usage of clonal expansions specifically, we observed a preference for V_H gene usage within each patient, but not between the two patients. Analysis of somatic hypermutations (SHMs) in these rearrangements showed that Dsg3-specific MBCs are highly mutated, comparable to infection-induced plasmablasts (Figure 2D) (Pauli et al., 2014; Priyamvada et al., 2016; Wrammert et al., 2011). Additionally, the ratio of replacement (R) to silent (S) mutations showed a high R/S ratio within the complementarity-determining regions (CDRs), well above the random frequency of 2.9 (Figure 2E) (Dörner et al., 1997), implying that these MBCs have undergone affinity maturation in germinal center reactions. Overall, these data suggest that a small pool of clonally restricted and affinity-matured MBCs dominates the autoimmune response in these patients.

To further characterize the Dsg3-specific responses, we recovered 44 mAbs generated from rearrangements observed during repertoire analysis of MBCs (Table S2). Using an ELISA, we found that 93% (41/44) of the mAbs bound to rDsg3 with high to moderate affinity (Figure 2F). To determine if these autoantibodies were cross reactive (Tiller et al., 2007), we also assessed binding against irrelevant antigens such as Dsg1, influenza HA, and cholera toxin B subunits (Figure 2F). Although Dsg1 is 65% homologous to Dsg3 (Amagai et al., 1992), only two

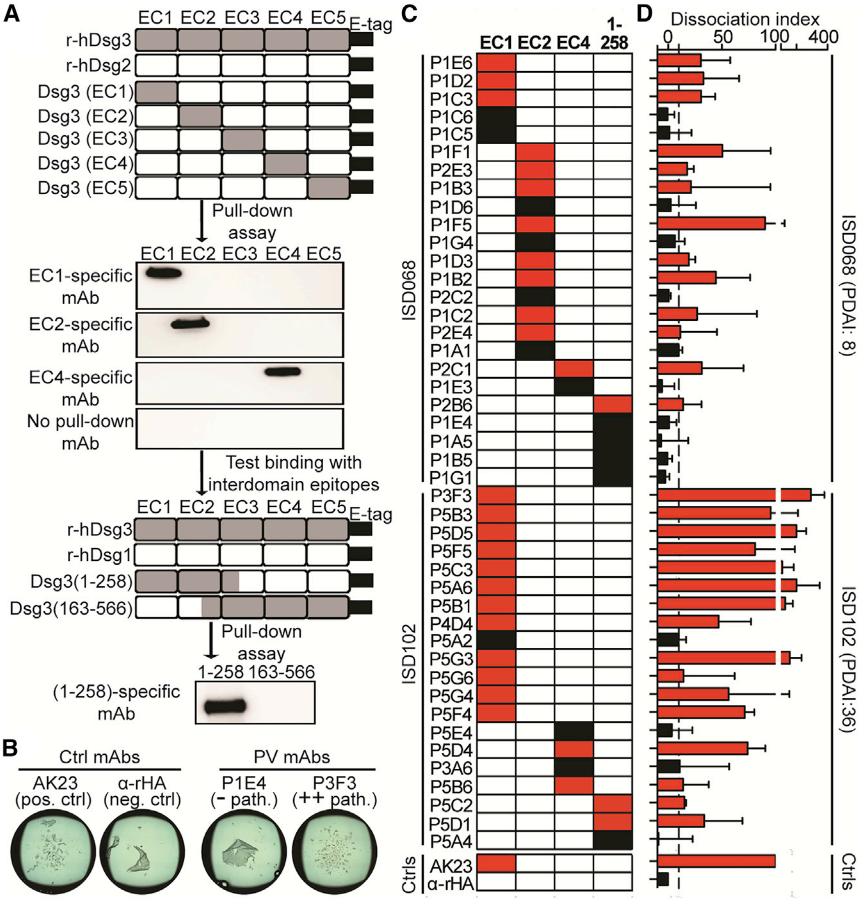

Figure 3. Pathogenic mAbs Bind the EC1, EC2, or EC4 Domains of Dsg3

(A) Chimeric proteins were generated by swapping EC domains from Dsg3 into the backbone of Dsg2 or sections of Dsg3 spanning the junction between domains into the backbone of Dsg1. Constructs were incubated with individual mAbs, followed by a pull-down assay to determine domain specificity. Representative results are shown.

(B) Representative keratinocyte dissociation assay.

(C) Most mAbs were specific for the EC1 and EC2 domains, with a subset targeting either the EC4 domain or interdomain epitopes.

(D) Pathogenic mAbs (shown in red) predominantly targeted the EC1 and EC2 domains, although several pathogenic EC4-specific mAbs were also identified. Dissociation index (DI) numbers are reported as median ± interquartile range (IQR) of four individual experimental repeats. Cut-off value (dotted line) was determined as the DI range of six irrelevant mAbs.

mAbs had binding activity toward Dsg1, while no mAbs cross reacted with influenza or cholera proteins. To assess if any of the mAbs bound to quaternary epitopes that may have been altered during the coating of rDsg3 to ELISA plates, we also tested their binding to native Dsg3 expressed on the surface of primary human keratinocytes (Figure S4A) (Saito et al., 2012; Stahley et al., 2014) and an immortalized cell line of human keratinocytes (HaCaTs) (Figure S4B) (Denning et al., 1998). These assays reaffirmed the binding of mAbs against Dsg3 (Figure S4C) and showed that the ELISA did not miss any Dsg3-specific mAbs. Thus, single-cell analysis of sorted Dsg3-specific MBCs provided a large set of antigen-specific mAbs for further functional analyses.

Human Pathogenic mAbs Bind to the EC1, EC2, or EC4 Domains of Dsg3

The extracellular portion of Dsg3 consists of five cadherin domain repeats (EC1−EC5) (Harrison et al., 2016). The EC1 domain lies at the N terminus of Dsg3, while the EC5 domain is closest to the cell surface (Amagai et al., 1991). To determine the domain specificity of the Dsg3-specific mAbs, we used chimeric proteins in which the extracellular domains from Dsg3 were swapped into the backbone of Dsg2, a homologous protein to which no cross reactivity has been described (Ohyama et al., 2012). These constructs were incubated with the individual mAbs, followed by a pull-down assay to determine specificity. For mAbs that did not bind these constructs, further testing was done using chimeric proteins in which sections of Dsg3 spanning the junction between several domains were swapped into a Dsg1 backbone (Futei et al., 2000) to determine if these mAbs bound to conformational interdomain epitopes (Figure 3A). In order to pair domain specificity of each mAb with functionality, we also tested their pathogenicities using an *in vitro* keratinocyte dissociation assay (Figure 3B) (Ishii et al., 2005). Pathogenicity measured by this *in vitro* assay often aligns with the *ex vivo* and *in vivo* pathogenic activity of mAbs (Di Zenzo et al., 2012; Ishii et al., 2005; Kawasaki et al., 2006; Payne et al., 2005). However, it is possible that the *in vitro* assay may not directly reflect the ability to cause suprabasal acantholysis of human skin tissue *in vivo*. Thus, mAbs described herein as having pathogenic activity will be in reference to their ability to cause *in vitro* keratinocyte dissociation.

We found that 68% (30/44) of the mAbs bound to either the EC1 or EC2 domains, while 14% (6/44) of mAbs bound to the EC4 domain, and 18% (8/44) bound to interdomain epitopes found within the first 258 amino acids at the N term of Dsg3 (Figure 3C). Additionally, we detected several mAbs with significantly higher pathogenic potency than the positive control AK23, a widely used mouse mAb with pathogenic activity both *in vitro* and *in vivo* (Ishii et al., 2005; Tsunoda et al., 2003). Interestingly, these highly pathogenic mAbs bound to the EC1 domain. It is thought that pathogenic antibodies primarily targeting EC1 and EC2 domains function by sterically hindering *trans* and *cis* adhesive interfaces found on Dsg3 (Amagai et al., 1992; Di Zenzo et al., 2012). In fact, most of the mAbs with pathogenic activity appear to target the EC1 or EC2 domains, although interdomain-binding mAbs that also bind the N terminus of Dsg3 have

A

Epitope	mAb	P1D2	P1E6	P1C3	P1C2	P1A1	P2E3	P1D6	P1D3	P2C2	P1G4	P1B3	P1F5	P1F1	P1B2	P2E4	P2B6	P2C1	P1E3	Heavy	Light	Pathogenicity
Domain		1	1	1	2	2	2	2	2	2	2	2	2	2	2	2		4	4			
ISD068 EC1A	P1D2	100	100	100	91	64	60	57	48	49	1	8	2	6	11	25	4	18	2	V_H1-58	V_K3-11	+
EC1A	P1E6	100	100	100	94	11	7	4	1	9	0	3	3	1	10	9	9	9	2	V_H5-10	V_K2D-29	+
EC1A	P1C3	79	73	88	69	1	12	7	13	20	2	14	3	3	17	19	4	17	8	V_H5-10	V_K2D-29	+
EC2A	P1C2	41	22	46	100	87	100	94	95	79	100	97	100	99	96	83	7	20	13	V_H4-39	V_K3-15	+
EC2A	P1A1	64	35	49	97	96	97	98	96	92	97	96	98	97	96	94	92	1	6	V_H4-34	V_K3-20	-
EC2A	P2E3	15	10	14	88	87	99	100	86	70	86	97	87	95	76	42	9	11	6	V_H1-2	V_K3-11	+
EC2A	P1D6	9	25	27	87	92	99	99	79	70	78	98	79	87	62	45	8	16	7	V_H1-2	V_K3-15	-
EC2A	P1D3	47	18	13	92	100	100	96	92	92	99	97	97	95	95	87	74	7	4	V_H1-2	V_K3-11	+
EC2A	P2C2	43	33	20	100	87	100	100	95	100	100	96	100	98	100	96	100	15	0	V_H1-2	V_K3-11	-
EC2A	P1G4	1	0	2	84	98	99	93	87	78	95	99	100	97	79	55	47	0	0	V_H4-39	V_K1-5	-
EC2A	P1B3	12	16	11	91	94	100	97	92	86	99	100	100	93	77	76	62	10	9	V_H4-39	V_K3-15	+
EC2A	P1F5	6	6	0	88	83	100	98	86	77	99	100	100	94	72	41	41	0	6	V_H4-39	V_K3-15	+
EC2A	P1F1	12	12	12	86	94	100	100	85	80	100	100	100	100	72	65	45	6	15	V_H4-39	V_K1-5	+
EC2A	P1B2	5	1	21	72	82	88	100	100	80	91	100	100	94	100	56	75	27	10	V_H4-39	V_K1-5	+
EC2A	P2E4	0	0	1	100	81	100	89	100	95	100	100	100	100	98	95	100	0	12	V_H4-39	V_K1-5	-
EC2A	P2B6	0	0	0	96	97	97	96	97	94	96	97	91	96	96	96	95	0	0	V_H4-39	V_K1-5	+
EC4A	P2C1	3	10	6	28	5	8	9	6	10	12	11	13	14	14	16	12	98	12	V_H4-39	V_K1-5	+
EC4B	P1E3	0	0	0	0	0	12	7	0	0	11	9	13	15	39	0	5	20	92	V_H3-33	V_K1-16	-

Percent Inhibition: 70-100%; 50-69%; 40-49%; <40%

B

Epitope	mAb	P5C3	P5B3	P5F5	P5F4	P5D5	P3F3	P5G3	P5A6	P5B1	P5A2	P3A6	P5D4	P5E4	P5B6	Heavy	Light	Pathogenicity
Domain		1	1	1	1	1	1	1	1	1	1	4	4	4	4			
ISD102 EC1B	P5C3	91	91	93	91	89	85	61	65	61	3	12	14	13	13	V_H3-15	V_L3-10	+
EC1B	P5B3	63	85	90	74	68	53	56	30	28	5	8	6	0	1	V_H3-15	V_L3-10	+
EC1B	P5F5	83	94	90	92	70	57	64	50	45	21	13	16	11	18	V_H3-15	V_L6-57	+
EC1B	P5F4	63	81	87	95	93	64	68	60	54	12	18	24	23	13	V_H3-15	V_L6-57	+
EC1A	P5D5	31	62	74	94	100	100	100	100	99	98	2	0	6	3	V_H1-46	V_K2-24	++
EC1A	P3F3	20	50	59	88	98	98	98	98	97	97	6	1	14	28	V_H1-46	V_K2-24	++
EC1A	P5G3	5	14	35	74	92	91	91	94	94	93	16	0	15	27	V_H1-46	V_K2-24	++
EC1A	P5A6	13	24	47	63	100	100	100	100	100	100	8	3	1	10	V_H1-46	V_K2-24	++
EC1A	P5B1	20	34	47	77	100	100	100	100	100	100	13	4	9	21	V_H1-46	V_K2-24	++
EC1A	P5A2	0	0	4	30	100	100	100	100	100	100	6	0	5	6	V_H3-53	V_L1-40	-
EC4B	P3A6	12	13	7	39	4	6	7	18	19	0	89	90	92	90	V_H1-69	V_K3-15	-
EC4B	P5D4	4	6	9	36	8	2	6	17	3	10	93	92	90	98	V_H3-66	V_K1-5	+
EC4B	P5E4	9	16	22	20	22	9	16	7	17	10	50	57	97	98	V_H4-61	V_L6-57	-
EC4B	P5B6	5	9	11	35	32	13	21	12	9	23	61	58	81	93	V_H4-61	V_L6-57	+

Figure 4. Dsg3-Specific mAbs Display a Restricted Repertoire and Bind to One or Two Sterically Distinct Epitopes in EC1, EC2, and EC4 Domains of Dsg3

A flow-based blocking assay was used to define the epitopes recognized by Dsg3-specific mAbs from (A) ISD068 and (B) ISD102. Shown is the average percent inhibition of binding of a biotinylated mAb when blocked with a 50-fold molar excess of an unlabeled mAb of two individual experimental repeats. Epitope name is located on the left of the chart. Colors at the top of the graph show domain specificity (orange = EC1; gray = EC2; gold = EC4; white = interdomain). Colors to the right of the graph represent mAbs in the same clonal expansion. V gene usage and pathogenicity are listed on the right of the panel. Pathogenicity is reported using the following cutoffs: – = DI ≤ 10; + = DI > 10; ++ = DI > 100.

low to no pathogenic activity. We also found several EC4-specific mAbs with pathogenic activity comparable to AK23. To our knowledge, this has not been previously described in the literature. Future research of how these mAbs cause pathogenicity will contribute to our understanding of PV pathogenesis (Figure 3D).

We noticed that mAbs derived from ISD102 appeared to show higher pathogenic potency than mAbs from ISD068. This observation correlates with the fact that ISD102 had more severe disease symptoms than ISD068, as measured by the Pemphigus Disease Area Index (PDAI) score, which is used clinically to assess the location, number, size, and damage of lesions (Daniel et al., 2012; Grover, 2011). ISD102 had a score of 36, while ISD068 scored 8 (Table S1). Because both patients have similar quantities of anti-Dsg3 serum antibodies (Table S1), it appears that the functional quality of individual mAbs correlates with disease severity. Overall, we found that while EC1 and EC2 are the primary target of mAbs with pathogenic activity, EC4-specific mAbs with pathogenic potential also exist.

Restricted V Gene Usage of mAbs Targeting a Limited Number of Individual Epitopes on Dsg3

To characterize epitope specificity of Dsg3-specific mAbs, we developed a flow cytometry-based competition assay by inhibiting the binding of a biotin-labeled mAb to HaCaTs using a 50-molar excess of unlabeled competitor antibodies. Only biotinylated-mAbs that had a mean fluorescence intensity (MFI) signal 1.5-fold greater than the negative control were used in this assay. Thus, we analyzed 18 mAbs from ISD068 (Figure 4A) and 14 mAbs from ISD102 (Figure 4B). Overall, we detected five non-overlapping epitopes: two epitopes within EC1 (named EC1A and EC1B), one epitope within EC2 (named EC2A), and two epitopes within EC4 (named EC4A and EC4B). As expected, mAbs derived from the same clonal expansion had similar epitope specificities. Additionally, no specific epitope was preferentially targeted by mAbs with pathogenic activity, though the highly pathogenic mAbs from ISD102 all bound the EC1A epitope. Similar epitopes were targeted in both of the donors analyzed, as mAbs that bound the EC1A and EC4B epitopes also competed between the two patients (Figures S5A and S5B). These five epitopes were also frequently targeted in other PV patients, especially the EC1A epitope, as determined by competition ELISAs where patient serum was used to block the binding of a representative biotinylated-mAb specific for one of the five major epitopes (Figure S5C). Interestingly, the EC1-specific mouse mAb AK23 did not appear to bind any of the five epitopes (Figure S5D). Future experiments comparing human- and mouse-derived Dsg3-specific mAbs will provide insight into whether there are species-dependent biases in immunodominant epitopes.

We observed restricted repertoires of mAbs that targeted specific epitopes. EC1A-binding mAbs primarily used V_H1-46/V_K2-24 genes (Figure 4B), while EC2A-binding mAbs used V_H1-2 and V_H4-39 heavy chain genes with a limited repertoire of V_K3-11, V_K3-15, and V_K1-5 light chain gene usage (Figure 4A). Interestingly, similar V gene usage has been described in previous publications. EC1-specific mAbs with V_H1-46 gene usage, some even using the V_K2-24 light chain (Cho et al., 2014; Yamagami et al., 2010), as well as EC2-specific mAbs with V_H1-2/V_K3-11 and V_H4-39/V_K3-15 gene usage (Di Zenzo et al., 2012), have been detected in other patients. This suggests that there may be genetic factors underlying autoantibody development in PV.

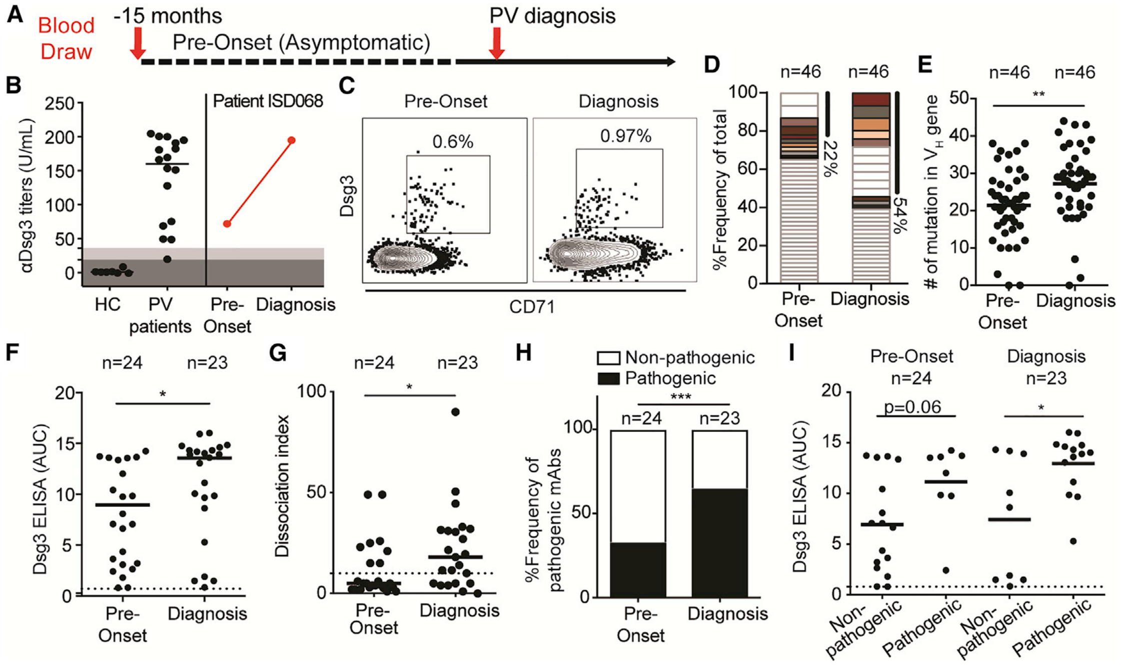

Figure 5. Dsg3-Specific MBCs Are Readily Detected in PV Patients Prior to Disease Onset and Undergo Extensive Affinity Maturation during Disease Development

(A) A unique sample from patient ISD068 was collected 15 months prior to diagnosis as part of a separate study.

(B) ISD068 had high Dsg3 titers prior to disease onset.

(C) Activated CD71+ Dsg3-specific MBCs were readily detected before disease onset.

(D) Single-cell analysis of Dsg3-specific MBCs (n = 46 per time point) shows that the frequency of clonally related cells increased 2-fold from pre-onset to diagnosis. Matching colors represent persisting clones detected at both time points.

(E) The number of SHMs found in the V_H gene increased significantly from pre-onset to diagnosis time point.

(F) Twenty-five mAbs were recovered from each time point. The relative affinity of the mAbs, reported as area under the curve (AUC), increased significantly over time. Three mAbs that did not bind Dsg3 were removed from subsequent analysis.

(G and H) Using a keratinocyte dissociation assay, we saw a significant increase in both (G) the pathogenic potency of the mAbs, as measured by DI number, and (H) the frequency of pathogenic mAbs detected in patient ISD068, when comparing pre-onset to diagnosis time point.

(I) Pathogenic mAbs had significantly higher affinities than non-pathogenic mAbs at diagnosis, but not pre-onset. A Mann-Whitney U test or a Fisher exact test was used where appropriate. *$p \leq 0.05$; **$p \leq 0.01$; ***$p \leq 0.001$.

Dsg3-Specific B Cell Responses Are Present Prior to Disease Onset, with Extensive Ongoing Affinity Maturation of MBCs Correlating with the Presentation of PV Symptoms

We had access to banked peripheral blood mononuclear cell (PBMC) and serum samples from donor ISD068 from a different study, collected 15 months prior to disease onset, during which the patient was asymptomatic (Figure 5A). This provided us with an exciting opportunity to investigate the development of Dsg3-specific B cells at a single-cell level. As previously described (Figure 1A), healthy controls have no anti-Dsg3 serum titers, whereas PV patients at diagnosis have high titers. However, even prior to disease onset, ISD068 displayed elevated titers against Dsg3 (72 U/mL), increasing 3-fold by the time the patient was diagnosed with PV (Figure 5B). Similarly, Dsg3-specific MBCs were also readily detected at pre-onset, albeit at a lower frequency than at diagnosis (Figure 5C). These cells displayed a CD71+ activated phenotype, suggesting that ongoing immune responses were occurring at least as early as 15 months prior to diagnosis.

We next performed single-cell analyses of Dsg3-specific MBCs to compare the responses at pre-onset to diagnosis, including 46 sequences per time point. The frequency of clonal expansions increased from 22% at pre-onset to 54% at the time of diagnosis. Additionally, we detected eight distinct clones that persisted from pre-onset to diagnosis (Figure 5D). While the limited number of cells analyzed does not allow for complete characterization, we noticed that five of the largest clonal expansions detected at diagnosis were derived from clones that were present pre-onset, suggesting that MBC responses at diagnosis largely derive from the maturation of existing clones generated prior to disease onset, and clonal focusing and antigenic selection drive disease pathogenesis. Additionally, there was a significant increase in the number of SHMs found in the V_H genes of Dsg3-specific MBCs at diagnosis compared to pre-onset (Figure 5E), confirming the continuous ongoing affinity maturation of MBCs over time. There was no significant difference in the R/S ratio in the CDRs of MBCs when comparing pre-onset to diagnosis (Figure S6A), suggesting that Dsg3-specific MBCs undergo affinity maturation at least as early as 15 months prior to disease onset.

Next, we generated mAbs from the pre-onset time point to compare their functionality to those at diagnosis (n = 25 per

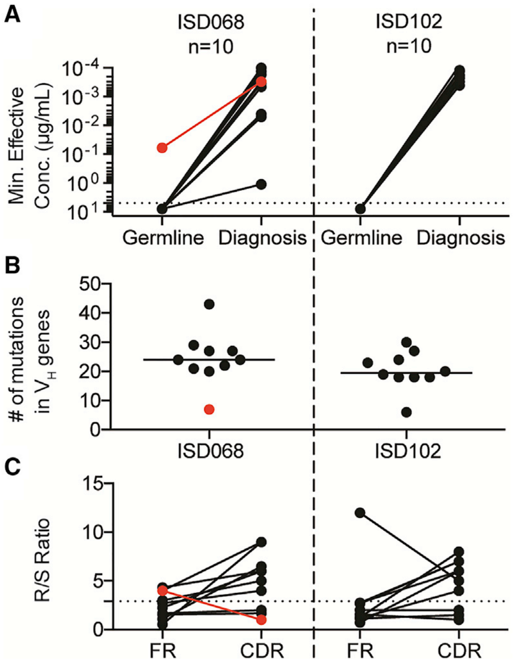

Figure 6. Somatic Hypermutation Is Necessary for Antibody Binding of Dsg3

(A) Ten mAbs from either patient ISD068 or ISD102 were reverted to their germline configuration. While the original, mutated mAbs showed high-affinity binding toward Dsg3, only one germline-reverted antibody retained the ability to bind Dsg3 (red line, mAb P1E3).

(B) Most mAbs had high levels of SHMs in the V_H gene, while P1E3 had very few mutations.

(C) P1E3 showed no enrichment of amino acid replacement mutations in the CDRs, compared to the other mAbs.

time point) (Table S2). Using an ELISA, we found that the relative affinity of mAbs against Dsg3 increased significantly from pre-onset to diagnosis (Figure 5F), confirming that affinity maturation occurred over time. Three mAbs with binding activity below the cut-off value were removed from subsequent analyses. We next assessed the *in vitro* pathogenicity of these mAbs and found that there was a significant increase in both the pathogenic potency (Figure 5G) and the frequency of mAbs with pathogenic activity found in the patient from pre-onset to diagnosis (Figure 5H). When comparing only mAbs derived from persisting clones found at both pre-onset and diagnosis, we found that they also showed a significant increase in affinity (Figure S6B), as well as a trend toward a significant increase in pathogenic activity (Figure S6C). Notably, pathogenic mAbs had significantly higher relative affinities than non-pathogenic mAbs at the diagnosis time point, but not at pre-onset (Figure 5I), suggesting that disease is driven by active selection for affinity and pathogenic activity. When using the flow-based competition assay, we found that the overall profile of overlapping epitopes at pre-onset (Figure S7A) was similar compared to at the diagnosis (Figure S7B). At both time points, about 70% of mAbs bound the EC2A epitope, while the rest of the mAbs largely targeted the EC1A epitope. No EC4A or EC4B epitope-binding mAbs were detected at the pre-onset time point. Thus, epitope spreading does not appear to contribute to PV pathogenesis. The repertoire of mAbs targeting the EC2A epitope at pre-onset was broader than the limited V_H4-39 and V_H1-2 gene usage that was detected at diagnosis, again suggesting that a small number of MBCs are being actively selected in response to Dsg3 and driving PV pathogenesis.

Dsg3-Specific mAbs Fail to Bind the Autoantigen in Their V Gene Germline Configurations

To define the origin of Dsg3-specific MBCs, we selected 10 mAbs each from ISD068 and ISD102 at the time of diagnosis and reverted them to their germline forms by removing all mutations detected in the V_H and V_L genes. These mAbs were selected to represent the epitope breadth described for each patient (Table S3). We found that almost all mAbs were unable to bind Dsg3 when reverted to its germline form (Figure 6A), suggesting that high-affinity Dsg3-specific MBCs originated either from non-autoreactive precursors or very low-affinity naive cells, and that SHMs are an essential component of developing PV autoantibodies. Despite previous publications that suggest that V_H1-46 gene usage allows mAbs to bind Dsg3, even in germline-reverted form (Cho et al., 2014), none of our V_H1-46 germline-reverted mAbs bound Dsg3 (Table S3). However, we detected a single mAb (ISD068 P1E3) that was able to bind Dsg3 when reverted to germline. This mAb uses a V_H3-33 rearrangement. Interestingly, the mutational load of P1E3 was notably lower compared to the other mAbs (Figure 6B) and did not show signs of having undergone antigenic selection, as evidenced by the low R/S ratio in the CDRs (Figure 6C). Overall, most Dsg3-specific B cells likely originate from non-autoreactive precursors through mechanisms such as molecular mimicry or by activation of very low-affinity naive cells, and SHMs are important to generate high-affinity Dsg3-specific antibody responses.

Pathogenic Potency Increases Synergistically through Targeting Multiple Domains in Dsg3

Based on our earlier observation that development of PV is mediated by the selection of pathogenic mAbs (Figure 5G) and an increase in the frequency of pathogenic mAbs (Figure 5H), we hypothesized that multiple mAbs with pathogenic activity will work synergistically to enhance the overall pathogenic potency. To address this issue, we selected three pathogenic mAbs that targeted distinct epitopes on Dsg3 and tested titrated amounts of antibodies to assess their individual *in vitro* pathogenicities compared to when the three mAbs were combined in equal ratios to each other (Figure 7A). We found that the combination of the three mAbs had a higher dissociation index (DI) compared to what we observed when measuring any single mAb alone. This synergy was especially clear when mAbs were tested at a concentration of 0.12 µg/mL. At this point, the EC1-specific mAb had robust pathogenic activity, while the EC2- and EC4-specific mAbs had activity barely above the cut-off value. However, combining the three mAbs increased the overall pathogenic signal, such that it was over 2-fold higher than the anticipated additive value of the DI numbers of the three mAbs at this concentration (Figure 7B). Overall, this suggests that multiple pathogenic antibodies can act synergistically to promote pathogenicity by targeting multiple Dsg3 epitopes.

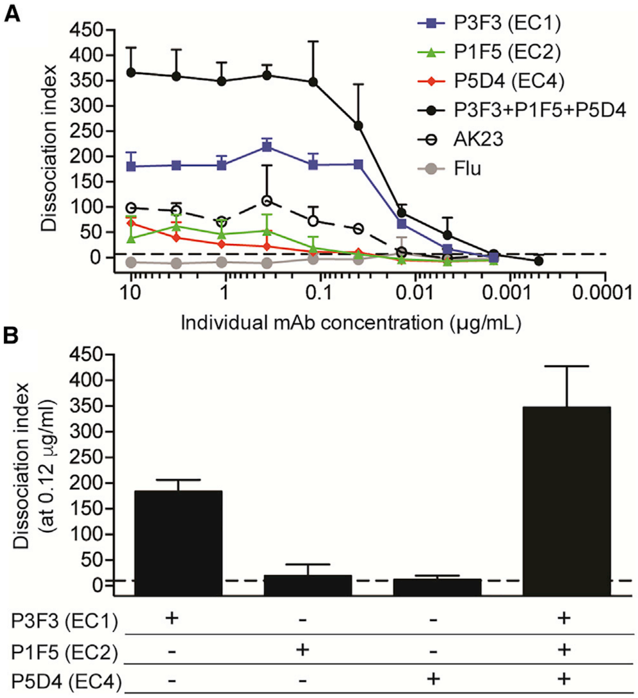

Figure 7. Synergistic Increase of Pathogenic Potency by Targeting Multiple Domains in Dsg3

(A) Pathogenic potency was measured by a keratinocyte dissociation assay with titration of individual, or a combination of, pathogenic mAbs targeting the EC1, EC2, or EC4 domains. The combined mAbs had a higher DI value than any single mAb alone.

(B) Combination of the three mAbs at 0.12 μg/mL showed synergy, with pathogenic signal 2-fold higher than the anticipated additive signal of the single mAbs at this concentration. DI numbers are reported as median ± IQR of four individual experimental repeats. The cut-off value (dotted line) was determined as the DI range of six irrelevant mAbs.

DISCUSSION

In the current study, we show that activated Dsg3-specific MBCs are readily detected in patients with active disease. The mAbs produced by these cells target a restricted number of distinct epitopes on Dsg3, have a restricted V gene repertoire with signs of extensive affinity maturation, and can work synergistically to enhance pathogenicity. A unique sample obtained over a year before the disease onset allowed us to show that in the absence of disease symptoms, activated Dsg3-specific MBCs were readily detected at pre-onset, and MBCs undergo extensive affinity maturation and clonal focusing over time during PV pathogenesis.

To understand the dynamics of B cell responses in PV patients, we analyzed serum antibody, MBC, and plasmablast responses at the time of diagnosis and at remission or relapse after Rtx therapy. All the PV patients responded to Rtx therapy with improved clinical symptoms, and most patients in remission lacked anti-Dsg3 titers. Our study, among others, has shown that while anti-Dsg3 titers drop with Rtx treatment, tetanus titers are unaffected (Mouquet et al., 2008). Thus, it is clear that disease is not mediated by bone marrow resident long-lived plasma cells, which do not express CD20 and are non-responsive to Rtx (Halliley et al., 2015). This suggests that while tolerance is disrupted in PV patients, allowing for the formation of autoreactive B cells, there are mechanisms in place that preclude their development into long-lived plasma cells. Based on our finding that the frequency of Dsg3-specific MBCs correlates positively with anti-Dsg3 serum titers detected in PV patients at the time of diagnosis, short-lived autoreactive plasmablasts are likely continuously replenished from the MBC pool. We found that these MBCs were activated and expressed the classic memory marker, CD27, excluding them from the IgD-CD27− atypical MBCs described in other autoimmune diseases (Claes et al., 2016; Jenks et al., 2018; Saadoun et al., 2013). Further phenotypic analyses of these cells will provide additional insight into their functional qualities. Interestingly, we and others were unable to detect Dsg3-specific plasmablasts in peripheral blood (Nishifuji et al., 2000). One interpretation of this is that activated MBCs can migrate to affected areas of skin and/or mucosae and differentiate locally into plasmablasts, which could be the source of the intercellular IgG deposits found in the epidermal lesions of PV patients (Witte et al., 2018). This hypothesis is supported by our observation that the frequency of Dsg3-specific MBCs correlates with serum autoantibody titers, as well as a recent study that showed that Dsg3-specific B cells are present in affected skin of PV patients (Yuan et al., 2017). It might also explain the two donors in clinical remission that still maintained high anti-Dsg3 serum titers despite Rtx-mediated depletion of peripheral B cells. It is possible that these two donors developed long-lived, Dsg3-specific plasma cells resistant to Rtx. However, the improved clinical responses in these patients may be a result of MBC depletion, resulting in a reduced differentiation of plasmablasts *in situ* and a decrease in pathogenic anti-Dsg3 antibodies in the skin. It is also possible that the large number of anti-Dsg3 serum antibodies from these remission patients was higher than the maximum signal detected by the clinical anti-Dsg3 ELISA, and decreases in anti-Dsg3 serum titers after Rtx treatment were undetectable due to limitations of the assay (Joly et al., 2017). While this issue remains highly speculative, future studies on skin-resident B cells will address these questions.

Single-cell repertoire analysis of the Dsg3-specific MBCs from ISD068 and ISD102 at the time of diagnosis showed that these cells were class switched, highly mutated, and clonally restricted. The majority of Dsg3-specific MBCs were IgG1, although IgG4 and IgA1 isotype usage was also observed, similar to the isotype usage described for serum autoantibodies in PV patients (Futei et al., 2001; Mentink et al., 2007; Spaeth et al., 2001). The preference for IgG1 and IgG4 isotype usage of Dsg3-specific antibodies indicates that isotypes impact antibody function. However, previous studies showed that F(ab)s (antibodies lacking the Fc domain) can cause PV-like symptoms in mouse models (Mascaró et al., 1997) and that changing the isotype of a mAb from IgG1 to IgG4 has no impact on its *in vitro* function (Lo et al., 2016). Thus, subclass bias may merely be a consequence of the cytokine milieu provided by Th1/Th2 Dsg3-specific T cell responses (Eming et al., 2014; Veldman et al., 2003). To our knowledge, no studies exist on the role of IgA isotype on the function of Dsg3-specific mAbs, although

this may be relevant considering that IgA1 has been associated with oral mucosal tissue (Korsrud and Brandtzaeg, 1980), which can be affected by PV. Future studies detailing how isotypes contribute to disease will be important in understanding the function of Dsg3-specific antibodies.

From the single-cell-derived sequences, we generated a panel of 44 mAbs from two patients. Of these, 41 mAbs were specific for Dsg3. Although autoreactive mAbs are often polyreactive (Tiller et al., 2007), these mAbs had no cross reactivity with irrelevant influenza HA and cholera toxin B subunit antigens. Only two mAbs had binding activity against Dsg1, which is 65% homologous to Dsg3 (Amagai et al., 1991). Domain-mapping experiments showed that most of the mAbs bound to the EC1 and EC2 domains of Dsg3, although several mAbs in both patients were found to bind the EC4 domain or interjunctional epitopes at the N terminus of Dsg3. Competition assays defined only one or two sterically distinct epitopes in each domain, with a total of five epitopes identified. Interestingly, several of the EC1-specific mAbs had pathogenic potency up to 3-fold higher than the archetypical pathogenic mouse mAb, AK23 (Tsunoda et al., 2003). These mAbs were derived from ISD102, who had worse disease symptoms than ISD068, suggesting that the pathogenic quality of individual mAbs contributes to disease severity. Competition assays showed that these mAbs bound the same EC1A epitope, which is distinct from the epitope targeted by the EC1-specific AK23. This suggests that epitopes relevant to human disease may not be recapitulated by mouse models, and further characterization of human- versus mouse-derived mAbs (Tsunoda et al., 2003) will provide a better understanding of PV pathogenesis.

The highly pathogenic mAbs targeting the EC1A epitope had a restricted repertoire usage of V_H1-46/V_K2-24. We also noticed a narrowing in repertoire usage of EC2-specific mAbs, which had a preference for V_H4-39 and V_H1-2 heavy chains, with V_K1-5 and V_K3-15 light chain usage. Although we did not detect the same rearrangements in both donors, despite similar epitopes being targeted, other laboratories have also described EC1-domain-specific mAbs using V_H1-46/V_K2-24 (Cho et al., 2014; Yeh et al., 2006) and EC2-domain-specific mAbs using V_H1-2/V_K1-5 and V_H4-39/V_K3-15 (Di Zenzo et al., 2012). Because restricted repertoires appear to be epitope dependent, repertoire analysis may provide information of which immune-dominant epitopes are targeted in patients, guiding future efforts to provide personalized treatment options for patients. Limited repertoire usage has also been described in other diseases, including influenza (Wrammert et al., 2011), HIV (Bonsignori et al., 2016), and systemic lupus erythematosus (SLE) (Pugh-Bernard et al., 2001; Tipton et al., 2015). Restricted V gene repertoires suggest that underlying genetic factors may drive antibody responses, such as certain germline V genes that may have inherent autoreactivity to Dsg3 (Cho et al., 2014), or the existence of a consensus sequence of pathogenic Dsg3-specific mAbs (Yamagami et al., 2010). Further studies on repertoire usage will contribute to our understanding of how autoantibodies break tolerance. A recent publication using proteomics approaches showed that only 20% of the anti-Dsg3 serum antibody repertoire overlapped with sequences obtained through phage display (APD) analyses of PBMCs (Chen et al., 2017), suggesting that serum autoantibodies likely derive from B cells not found in circulation. However, studies of influenza and tetanus responses have shown similar differences between single-cell data and proteomics data (Lavinder et al., 2014; Lee et al., 2016). In the case of tetanus, dominant clonal expansions found in single-cell analyses are commonly represented in proteomics analyses, while rare rearrangements are less so. While single-cell technology has been used extensively over the last decade to address repertoire questions and generate and characterize antigen-specific mAbs, these approaches all have inherent technical shortcomings. In the case of APD, this approach is a very powerful tool to assess a large number of PBMCs to find rare antibody specificities. However, repertoire analysis can be skewed by over-representation of plasmablast-derived mRNA in the PBMC libraries, extensive panning that can alter the repertoire breadth, and a lack of information on the cognate heavy and light chain pairing of individual cells. Overall, our repertoire data align with the proteomics-based repertoire breadth observed by Chen et al. (2017), but future studies to directly compare the relationship among autoreactive peripheral MBCs, tissue-resident B cells, and serum antibodies will further clarify the relationship among these compartments.

While most of the pathogenic mAbs bound the EC1 and EC2 domains, we also detected EC4-domain-specific mAbs with pathogenic activity comparable to AK23. The epitopes mediating *trans* and *cis* interactions lie in the EC1 and EC2 domains (Boggon et al., 2002; Harrison et al., 2016; Wu et al., 2010), and EC1- and EC2-specific antibodies are thought to cause pathogenicity by sterically hindering these adhesive interactions (Di Zenzo et al., 2012; Tsunoda et al., 2003). However, the detection of EC4-specific mAbs with pathogenic activity suggests that additional mechanisms contribute to pathogenicity. Distinct from other cadherins, Dsg3 has a prominent kink present between the EC3 and EC4 domains induced by calcium-binding sites (Harrison et al., 2016). It is possible that this allows for optimal presentation of epitopes within the EC3 or EC4 domains, and binding these epitopes may induce conformational changes to Dsg3 and impact its function. Additionally, EC4-specific mAbs may potently engage signaling pathways and induce the endocytosis and degradation of Dsg3 (Calkins et al., 2006; Mao et al., 2011; Saito et al., 2012; Stahley et al., 2014, 2016). Current efforts are being made to develop therapies targeting the depletion of pathogenic Dsg3-specific B cells using chimeric autoantibody receptor T cells, possibly in a domain-specific manner (Ellebrecht et al., 2016). While previous studies suggest that domains EC1 and EC2 are primarily targeted by pathogenic mAbs (Amagai et al., 1992; Di Zenzo et al., 2012), our data show EC4-specific pathogenic mAbs exist, and comprehensive therapies that deplete pathogenic Dsg3-specific B cells regardless of domain specificity may be necessary to treat disease. Ongoing efforts to determine how EC4-specific mAbs cause pathogenicity both *in vitro* and *in vivo* may elucidate mechanisms underlying disease pathogenesis and guide the discovery of long-lasting therapies for PV patients.

We had access to a unique paired sample collected from patient ISD068 15 months prior to PV onset, when the patient was asymptomatic. This gave us an opportunity to study the development of autoimmune responses at a single-cell level. We found

that both Dsg3-specific serum titers and activated MBCs could be readily detected 15 months prior to developing disease. Even at pre-onset, MBCs had high levels of clonality and SHMs, suggesting that these cells had already undergone antigen selection and affinity maturation. In contrast, germline-reverted mAbs were generally unable to bind Dsg3, indicating that disease-causing MBCs likely originate from non-autoreactive B cell precursors or naive cells of very low affinity. It remains unclear what initial trigger activates these precursors, although it is possible that molecular mimicry may drive initial responses against Dsg3 (Cho et al., 2016; Lin et al., 2019), which is also a mechanism proposed for the related endemic form of pemphigus, fogo selvagem (Qian et al., 2012). While previous publications have shown that some germline-reverted mAbs with V_H1-46 gene usage are able to bind to Dsg3 (Cho et al., 2014), none of our V_H1-46 mAbs retained binding activity in germline form. However, we did detect one mAb that bound Dsg3 in its germline-reverted form. This mAb was distinct in its use of the V_H3-33 gene and EC4-specificity but was similar to the previously described germline-reverted V_H1-46 antibodies in that our patient-derived mAb also had a low number of mutations, and R/S analysis showed no signs of affinity selection. Thus, it is also possible that rare low-affinity naive B cells can be directly recruited and activated in response to Dsg3. Identification of additional germline-reverted mAbs that can bind to Dsg3 or detection of low-affinity antigen-binding naive B cells (Taylor et al., 2015) will provide further insight into the origins of Dsg3-specific B cells.

By generating additional Dsg3-specific mAbs from the pre-onset time point, we could compare the functionality of these mAbs to those at diagnosis. There was a significant increase in both the frequency of clonal expansions as well as the number of SHMs, suggesting that continuous and ongoing affinity maturation are important for the development of PV. This was supported by our findings that both affinity and pathogenic potency of mAbs increased from pre-onset to diagnosis, and pathogenic mAbs derived from the diagnosis time point had higher affinity for Dsg3 than non-pathogenic mAbs. Additionally, there was a marked increase in the number of mAbs with pathogenic activity found in patient ISD068 over time. Thus, it appears that affinity maturation drives the selection of pathogenic antibodies, and accumulation of pathogenic mAbs causes onset of PV symptoms. We identified four persisting clones that were non-pathogenic at pre-onset but showed pathogenic activity at the time of diagnosis. This was is in addition to three clonal families detected in diagnosis patients, in which one member was non-pathogenic and the other was pathogenic. Because the CDRs of these mAbs were extensively mutated, we were unable to associate a single replacement mutation with a gain in pathogenic function. Future experiments will identify which mutations are relevant for the pathogenic function of mAbs. Previous studies of the autoimmune diseases fogo selvagem (Li et al., 2003) and SLE (Arbuckle et al., 2003) have also shown that serum autoantibodies can be detected in patients prior to the disease onset. Longitudinal characterization of these patients suggests that epitope spreading and the accumulation of autoantibodies drive disease pathogenesis. However, we did not detect epitope spreading of the mAbs derived from our PV patient, similar to previous studies that show a lack of epitope spreading in PV patients as they experience remission or relapse (Ohyama et al., 2012). We also found that pathogenic mAbs targeting different epitopes work synergistically to increase pathogenic potency, suggesting that disease can be initiated or enhanced by engaging multiple Dsg3 epitopes. Further experiments focused on which combinations of mAbs drive a maximal pathogenic response and the mechanism underlying synergy will be important in understanding how this process works *in vivo*.

Our data suggest that PV pathogenesis is mediated in part by a complex evolution of Dsg3-specific MBCs. While initial autoreactive responses appear to be triggered either by molecular mimicry or through very low-affinity precursors, antigen selection is necessary for the development of high-affinity pathogenic MBCs. Pathogenic mAbs produced by these cells can work synergistically to enhance the overall pathogenic potency and drive the presentation of PV symptoms. As disease progresses and MBCs continue to undergo antigen selection, individual mAbs show an increase in pathogenic potency, which contributes to an increase in disease severity. Overall, these findings provide a deeper understanding of the mechanism underlying how Dsg3-specific mAbs develop and cause PV pathogenesis, and they may have important implications for improving diagnostic tools and therapeutic approaches for PV patients.

STAR★METHODS

Detailed methods are provided in the online version of this paper and include the following:

- KEY RESOURCES TABLE
- LEAD CONTACT AND MATERIALS AVAILABILITY
- EXPERIMENTAL MODEL AND SUBJECT DETAILS
  - Patients, study design, and approvals
  - Cell culture
- METHOD DETAILS
  - PBMC and plasma isolation
  - ELISA
  - Memory B cell assay
  - Flow cytometry analysis and single-cell sorting
  - Plasmablast ELISPOT assay
  - Subclass-specific ELISA
  - Immunofluorescence staining
  - Generation of recombinant monoclonal antibodies
  - Repertoire analysis
  - Keratinocyte Dissociation Assay
  - Domain mapping
  - HaCaT binding and competition assay
  - Blocking ELISA
- QUANTIFICATION AND STATISTICAL ANALYSIS
  - Statistical analysis
- DATA AND CODE AVAILABILITY

SUPPLEMENTAL INFORMATION

Supplemental Information can be found online at https://doi.org/10.1016/j.celrep.2019.06.066.

ACKNOWLEDGMENTS

We thank R. Karaffa at the Emory School of Medicine flow cytometry core and A. Rae at Emory Pediatric flow cytometry core for their technical assistance with cell-sorting experiments. We thank Drs. R. Ahmed and M. Slifka for providing samples used in this paper. This study was supported by grants from the NIH (Human Immunology Pilot award under U19 AI057266 (J.W.); the International Pemphigus and Pemphigoid Foundation (J.W.); the Charles and Daneen Stiefel Scholar Award from the Dermatology Foundation (A.P.); the Deutsche Forschungsgemeinschaft (FOR 2497 PEGASUS, M.H. and T.S.); and the National Center for Advancing Translational Sciences of the NIH under award TL1TR001880 (N.R.)

AUTHOR CONTRIBUTIONS

A.C. carried out experiments, analyzed the data, and wrote the manuscript. A.L.C. contributed to pathogenicity and IF assays. N.A.R. carried out domain-mapping experiments. Z.M. and M.A. contributed to the competition assays. C.N. and A.L. contributed to ELISA assays. R.C.K., A.S., and G.M. contributed to the analysis of isotype usage. B.B. contributed to analysis of data. S.Z. contributed to pathogenicity assays. T.S. and M.H. provided essential reagents and feedback on the manuscript. A.S.P., R.F., and A.P.K. contributed to analysis, critical discussion, and editing of the manuscript. J.W. conceived the research, oversaw the experiments and data analysis, and wrote the manuscript.

DECLARATION OF INTERESTS

A.S.P. has served as a consultant for Syntimmune, holds equity in Cabaletta Bio, and is an inventor on patents licensed by Novartis and Cabaletta Bio focused on targeted cellular therapy of autoimmunity. M.H. has received unrestricted grants from Topas Therapeuticals, Fresenius and Biotest, and has served as a consultant for Argenx and Allmirall. R.F. has served as a consultant for Cabeletta, Bioverative, and Celtaxys. The other authors declare no competing interests.

Received: February 10, 2019
Revised: May 22, 2019
Accepted: June 18, 2019
Published: July 23, 2019

REFERENCES

Amagai, M., Klaus-Kovtun, V., and Stanley, J.R. (1991). Autoantibodies against a novel epithelial cadherin in pemphigus vulgaris, a disease of cell adhesion. Cell *67*, 869–877.

Amagai, M., Karpati, S., Prussick, R., Klaus-Kovtun, V., and Stanley, J.R. (1992). Autoantibodies against the amino-terminal cadherin-like binding domain of pemphigus vulgaris antigen are pathogenic. J. Clin. Invest. *90*, 919–926.

Amagai, M., Hashimoto, T., Shimizu, N., and Nishikawa, T. (1994). Absorption of pathogenic autoantibodies by the extracellular domain of pemphigus vulgaris antigen (Dsg3) produced by baculovirus. J. Clin. Invest. *94*, 59–67.

Amagai, M., Koch, P.J., Nishikawa, T., and Stanley, J.R. (1996). Pemphigus vulgaris antigen (desmoglein 3) is localized in the lower epidermis, the site of blister formation in patients. J. Invest. Dermatol. *106*, 351–355.

Amagai, M., Tsunoda, K., Suzuki, H., Nishifuji, K., Koyasu, S., and Nishikawa, T. (2000). Use of autoantigen-knockout mice in developing an active autoimmune disease model for pemphigus. J. Clin. Invest. *105*, 625–631.

Anhalt, G.J., Labib, R.S., Voorhees, J.J., Beals, T.F., and Diaz, L.A. (1982). Induction of pemphigus in neonatal mice by passive transfer of IgG from patients with the disease. N. Engl. J. Med. *306*, 1189–1196.

Arbuckle, M.R., McClain, M.T., Rubertone, M.V., Scofield, R.H., Dennis, G.J., James, J.A., and Harley, J.B. (2003). Development of autoantibodies before the clinical onset of systemic lupus erythematosus. N. Engl. J. Med. *349*, 1526–1533.

Beutner, E.H., and Jordon, R.E. (1964). Demonstration of Skin Antibodies in Sera of Pemphigus Vulgaris Patients by Indirect Immunofluorescent Staining. Proc. Soc. Exp. Biol. Med. *117*, 505–510.

Boggon, T.J., Murray, J., Chappuis-Flament, S., Wong, E., Gumbiner, B.M., and Shapiro, L. (2002). C-cadherin ectodomain structure and implications for cell adhesion mechanisms. Science *296*, 1308–1313.

Bonsignori, M., Zhou, T., Sheng, Z., Chen, L., Gao, F., Joyce, M.G., Ozorowski, G., Chuang, G.Y., Schramm, C.A., Wiehe, K., et al.; NISC Comparative Sequencing Program (2016). Maturation Pathway from Germline to Broad HIV-1 Neutralizer of a CD4-Mimic Antibody. Cell *165*, 449–463.

Breedveld, F., Agarwal, S., Yin, M., Ren, S., Li, N.F., Shaw, T.M., and Davies, B.E. (2007). Rituximab pharmacokinetics in patients with rheumatoid arthritis: B-cell levels do not correlate with clinical response. J. Clin. Pharmacol. *47*, 1119–1128.

Calkins, C.C., Setzer, S.V., Jennings, J.M., Summers, S., Tsunoda, K., Amagai, M., and Kowalczyk, A.P. (2006). Desmoglein endocytosis and desmosome disassembly are coordinated responses to pemphigus autoantibodies. J. Biol. Chem. *281*, 7623–7634.

Chen, J., Zheng, Q., Hammers, C.M., Ellebrecht, C.T., Mukherjee, E.M., Tang, H.Y., Lin, C., Yuan, H., Pan, M., Langenhan, J., et al. (2017). Proteomic Analysis of Pemphigus Autoantibodies Indicates a Larger, More Diverse, and More Dynamic Repertoire than Determined by B Cell Genetics. Cell Rep. *18*, 237–247.

Cho, M.J., Lo, A.S.Y., Mao, X., Nagler, A.R., Ellebrecht, C.T., Mukherjee, E.M., Hammers, C.M., Choi, E.J., Sharma, P.M., Uduman, M., et al. (2014). Shared VH1-46 gene usage by pemphigus vulgaris autoantibodies indicates common humoral immune responses among patients. Nat. Commun. *5*, 4167.

Cho, M.J., Ellebrecht, C.T., Hammers, C.M., Mukherjee, E.M., Sapparapu, G., Boudreaux, C.E., McDonald, S.M., Crowe, J.E., Jr., and Payne, A.S. (2016). Determinants of VH1-46 Cross-Reactivity to Pemphigus Vulgaris Autoantigen Desmoglein 3 and Rotavirus Antigen VP6. J. Immunol. *197*, 1065–1073.

Cho, A., Bradley, B., Kauffman, R., Priyamvada, L., Kovalenkov, Y., Feldman, R., and Wrammert, J. (2017). Robust memory responses against influenza vaccination in pemphigus patients previously treated with rituximab. JCI Insight *2*, 93222.

Claes, N., Fraussen, J., Vanheusden, M., Hellings, N., Stinissen, P., Van Wijmeersch, B., Hupperts, R., and Somers, V. (2016). Age-Associated B Cells with Proinflammatory Characteristics Are Expanded in a Proportion of Multiple Sclerosis Patients. J. Immunol. *197*, 4576–4583.

Colliou, N., Picard, D., Caillot, F., Calbo, S., Le Corre, S., Lim, A., Lemercier, B., Le Mauff, B., Maho-Vaillant, M., Jacquot, S., et al. (2013). Long-term remissions of severe pemphigus after rituximab therapy are associated with prolonged failure of desmoglein B cell response. Sci. Transl. Med. *5*, 175ra30.

Corti, D., and Lanzavecchia, A. (2014). Efficient Methods To Isolate Human Monoclonal Antibodies from Memory B Cells and Plasma Cells. Microbiol. Spectr. *2*.

Culton, D.A., McCray, S.K., Park, M., Roberts, J.C., Li, N., Zedek, D.C., Anhalt, G.J., Cowley, D.O., Liu, Z., and Diaz, L.A. (2015). Mucosal pemphigus vulgaris anti-Dsg3 IgG is pathogenic to the oral mucosa of humanized Dsg3 mice. J. Invest. Dermatol. *135*, 1590–1597.

Daniel, B.S., Hertl, M., Werth, V.P., Eming, R., and Murrell, D.F. (2012). Severity score indexes for blistering diseases. Clin. Dermatol. *30*, 108–113.

Delva, E., Tucker, D.K., and Kowalczyk, A.P. (2009). The desmosome. Cold Spring Harb. Perspect. Biol. *1*, a002543.

Denning, M.F., Guy, S.G., Ellerbroek, S.M., Norvell, S.M., Kowalczyk, A.P., and Green, K.J. (1998). The expression of desmoglein isoforms in cultured human keratinocytes is regulated by calcium, serum, and protein kinase C. Exp. Cell Res. *239*, 50–59.

Di Zenzo, G., Di Lullo, G., Corti, D., Calabresi, V., Sinistro, A., Vanzetta, F., Didona, B., Cianchini, G., Hertl, M., Eming, R., et al. (2012). Pemphigus

autoantibodies generated through somatic mutations target the desmoglein-3 cis-interface. J. Clin. Invest. *122*, 3781–3790.

Ding, X., Aoki, V., Mascaro, J.M., Jr., Lopez-Swiderski, A., Diaz, L.A., and Fairley, J.A. (1997). Mucosal and mucocutaneous (generalized) pemphigus vulgaris show distinct autoantibody profiles. J. Invest. Dermatol. *109*, 592–596.

Dörner, T., Brezinschek, H.P., Brezinschek, R.I., Foster, S.J., Domiati-Saad, R., and Lipsky, P.E. (1997). Analysis of the frequency and pattern of somatic mutations within nonproductively rearranged human variable heavy chain genes. J. Immunol. *158*, 2779–2789.

Ellebedy, A.H., Jackson, K.J., Kissick, H.T., Nakaya, H.I., Davis, C.W., Roskin, K.M., McElroy, A.K., Oshansky, C.M., Elbein, R., Thomas, S., et al. (2016). Defining antigen-specific plasmablast and memory B cell subsets in human blood after viral infection or vaccination. Nat. Immunol. *17*, 1226–1234.

Ellebrecht, C.T., Bhoj, V.G., Nace, A., Choi, E.J., Mao, X., Cho, M.J., Di Zenzo, G., Lanzavecchia, A., Seykora, J.T., Cotsarelis, G., et al. (2016). Reengineering chimeric antigen receptor T cells for targeted therapy of autoimmune disease. Science *353*, 179–184.

Ellebrecht, C.T., Mukherjee, E.M., Zheng, Q., Choi, E.J., Reddy, S.G., Mao, X., and Payne, A.S. (2018). Autoreactive IgG and IgA B Cells Evolve through Distinct Subclass Switch Pathways in the Autoimmune Disease Pemphigus Vulgaris. Cell Rep. *24*, 2370–2380.

Eming, R., Hennerici, T., Bäcklund, J., Feliciani, C., Visconti, K.C., Willenborg, S., Wohde, J., Holmdahl, R., Sønderstrup, G., and Hertl, M. (2014). Pathogenic IgG antibodies against desmoglein 3 in pemphigus vulgaris are regulated by HLA-DRB1*04:02-restricted T cells. J. Immunol. *193*, 4391–4399.

Futei, Y., Amagai, M., Sekiguchi, M., Nishifuji, K., Fujii, Y., and Nishikawa, T. (2000). Use of domain-swapped molecules for conformational epitope mapping of desmoglein 3 in pemphigus vulgaris. J. Invest. Dermatol. *115*, 829–834.

Futei, Y., Amagai, M., Ishii, K., Kuroda-Kinoshita, K., Ohya, K., and Nishikawa, T. (2001). Predominant IgG4 subclass in autoantibodies of pemphigus vulgaris and foliaceus. J. Dermatol. Sci. *26*, 55–61.

Grover, S. (2011). Scoring systems in pemphigus. Indian J. Dermatol. *56*, 145–149.

Halliley, J.L., Tipton, C.M., Liesveld, J., Rosenberg, A.F., Darce, J., Gregoretti, I.V., Popova, L., Kaminiski, D., Fucile, C.F., Albizua, I., et al. (2015). Long-Lived Plasma Cells Are Contained within the CD19(-)CD38(hi)CD138(+) Subset in Human Bone Marrow. Immunity *43*, 132–145.

Hammers, C.M., and Stanley, J.R. (2014). Antibody phage display: technique and applications. J. Invest. Dermatol. *134*, 1–5.

Hammers, C.M., Chen, J., Lin, C., Kacir, S., Siegel, D.L., Payne, A.S., and Stanley, J.R. (2015). Persistence of anti-desmoglein 3 IgG(+) B-cell clones in pemphigus patients over years. J. Invest. Dermatol. *135*, 742–749.

Harman, K.E., Seed, P.T., Gratian, M.J., Bhogal, B.S., Challacombe, S.J., and Black, M.M. (2001). The severity of cutaneous and oral pemphigus is related to desmoglein 1 and 3 antibody levels. Br. J. Dermatol. *144*, 775–780.

Harrison, O.J., Brasch, J., Lasso, G., Katsamba, P.S., Ahlsen, G., Honig, B., and Shapiro, L. (2016). Structural basis of adhesive binding by desmocollins and desmogleins. Proc. Natl. Acad. Sci. USA *113*, 7160–7165.

Ishii, K., Harada, R., Matsuo, I., Shirakata, Y., Hashimoto, K., and Amagai, M. (2005). In vitro keratinocyte dissociation assay for evaluation of the pathogenicity of anti-desmoglein 3 IgG autoantibodies in pemphigus vulgaris. J. Invest. Dermatol. *124*, 939–946.

Jenks, S.A., Cashman, K.S., Zumaquero, E., Marigorta, U.M., Patel, A.V., Wang, X., Tomar, D., Woodruff, M.C., Simon, Z., Bugrovsky, R., et al. (2018). Distinct Effector B Cells Induced by Unregulated Toll-like Receptor 7 Contribute to Pathogenic Responses in Systemic Lupus Erythematosus. Immunity *49*, 725–739. e6.

Joly, P., Maho-Vaillant, M., Prost-Squarcioni, C., Hebert, V., Houivet, E., Calbo, S., Caillot, F., Golinski, M.L., Labeille, B., Picard-Dahan, C., et al.; French study group on autoimmune bullous skin diseases (2017). First-line rituximab combined with short-term prednisone versus prednisone alone for the treatment of pemphigus (Ritux 3): a prospective, multicentre, parallel-group, open-label randomised trial. Lancet *389*, 2031–2040.

Kauffman, R.C., Bhuiyan, T.R., Nakajima, R., Mayo-Smith, L.M., Rashu, R., Hoq, M.R., Chowdhury, F., Khan, A.I., Rahman, A., Bhaumik, S.K., et al. (2016). Single-Cell Analysis of the Plasmablast Response to Vibrio cholerae Demonstrates Expansion of Cross-Reactive Memory B Cells. MBio *7*, e02021-16.

Kawasaki, H., Tsunoda, K., Hata, T., Ishii, K., Yamada, T., and Amagai, M. (2006). Synergistic pathogenic effects of combined mouse monoclonal anti-desmoglein 3 IgG antibodies on pemphigus vulgaris blister formation. J. Invest. Dermatol. *126*, 2621–2630.

Koch, P.J., Mahoney, M.G., Ishikawa, H., Pulkkinen, L., Uitto, J., Shultz, L., Murphy, G.F., Whitaker-Menezes, D., and Stanley, J.R. (1997). Targeted disruption of the pemphigus vulgaris antigen (desmoglein 3) gene in mice causes loss of keratinocyte cell adhesion with a phenotype similar to pemphigus vulgaris. J. Cell Biol. *137*, 1091–1102.

Korsrud, F.R., and Brandtzaeg, P. (1980). Quantitative immunohistochemistry of immunoglobulin- and J-chain-producing cells in human parotid and submandibular salivary glands. Immunology *39*, 129–140.

Kowalczyk, A.P., and Green, K.J. (2013). Structure, function, and regulation of desmosomes. Prog. Mol. Biol. Transl. Sci. *116*, 95–118.

Lavinder, J.J., Wine, Y., Giesecke, C., Ippolito, G.C., Horton, A.P., Lungu, O.I., Hoi, K.H., DeKosky, B.J., Murrin, E.M., Wirth, M.M., et al. (2014). Identification and characterization of the constituent human serum antibodies elicited by vaccination. Proc. Natl. Acad. Sci. USA *111*, 2259–2264.

Lee, J., Boutz, D.R., Chromikova, V., Joyce, M.G., Vollmers, C., Leung, K., Horton, A.P., DeKosky, B.J., Lee, C.H., Lavinder, J.J., et al. (2016). Molecular-level analysis of the serum antibody repertoire in young adults before and after seasonal influenza vaccination. Nat. Med. *22*, 1456–1464.

Lever, W.F. (1953). Pemphigus. Medicine (Baltimore) *32*, 1–123.

Li, N., Aoki, V., Hans-Filho, G., Rivitti, E.A., and Diaz, L.A. (2003). The role of intramolecular epitope spreading in the pathogenesis of endemic pemphigus foliaceus (fogo selvagem). J. Exp. Med. *197*, 1501–1510.

Lin, L., Moran, T.P., Peng, B., Yang, J., Culton, D.A., Che, H., Jiang, S., Liu, Z., Geng, S., Zhang, Y., et al. (2019). Walnut antigens can trigger autoantibody development in patients with pemphigus vulgaris through a "hit-and-run" mechanism. J. Allergy Clin. Immunol. Published online May 6, 2019. https://doi.org/10.1016/j.jaci.2019.04.020.

Lo, A.S., Mao, X., Mukherjee, E.M., Ellebrecht, C.T., Yu, X., Posner, M.R., Payne, A.S., and Cavacini, L.A. (2016). Pathogenicity and Epitope Characteristics Do Not Differ in IgG Subclass-Switched Anti-Desmoglein 3 IgG1 and IgG4 Autoantibodies in Pemphigus Vulgaris. PLoS ONE *11*, e0156800.

Mao, X., Sano, Y., Park, J.M., and Payne, A.S. (2011). p38 MAPK activation is downstream of the loss of intercellular adhesion in pemphigus vulgaris. J. Biol. Chem. *286*, 1283–1291.

Mascaró, J.M., Jr., España, A., Liu, Z., Ding, X., Swartz, S.J., Fairley, J.A., and Diaz, L.A. (1997). Mechanisms of acantholysis in pemphigus vulgaris: role of IgG valence. Clin. Immunol. Immunopathol. *85*, 90–96.

Maurer, M.A., Rakocevic, G., Leung, C.S., Quast, I., Lukačišin, M., Goebels, N., Münz, C., Wardemann, H., Dalakas, M., and Lünemann, J.D. (2012). Rituximab induces sustained reduction of pathogenic B cells in patients with peripheral nervous system autoimmunity. J. Clin. Invest. *122*, 1393–1402.

Mentink, L.F., de Jong, M.C., Kloosterhuis, G.J., Zuiderveen, J., Jonkman, M.F., and Pas, H.H. (2007). Coexistence of IgA antibodies to desmogleins 1 and 3 in pemphigus vulgaris, pemphigus foliaceus and paraneoplastic pemphigus. Br. J. Dermatol. *156*, 635–641.

Mouquet, H., Musette, P., Gougeon, M.L., Jacquot, S., Lemercier, B., Lim, A., Gilbert, D., Dutot, I., Roujeau, J.C., D'Incan, M., et al. (2008). B-cell depletion immunotherapy in pemphigus: effects on cellular and humoral immune responses. J. Invest. Dermatol. *128*, 2859–2869.

Muto, K., Matsui, N., Unai, Y., Sakai, W., Haji, S., Udaka, K., Miki, H., Furukawa, T., Abe, M., and Kaji, R. (2017). Memory B cell resurgence requires repeated rituximab in myasthenia gravis. Neuromuscul. Disord. *27*, 918–922.

Nishifuji, K., Amagai, M., Kuwana, M., Iwasaki, T., and Nishikawa, T. (2000). Detection of antigen-specific B cells in patients with pemphigus vulgaris by

enzyme-linked immunospot assay: requirement of T cell collaboration for autoantibody production. J. Invest. Dermatol. *114*, 88–94.

Ohyama, B., Nishifuji, K., Chan, P.T., Kawaguchi, A., Yamashita, T., Ishii, N., Hamada, T., Dainichi, T., Koga, H., Tsuruta, D., et al. (2012). Epitope spreading is rarely found in pemphigus vulgaris by large-scale longitudinal study using desmoglein 2-based swapped molecules. J. Invest. Dermatol. *132*, 1158–1168.

Pauli, N.T., Kim, H.K., Falugi, F., Huang, M., Dulac, J., Henry Dunand, C., Zheng, N.Y., Kaur, K., Andrews, S.F., Huang, Y., et al. (2014). Staphylococcus aureus infection induces protein A-mediated immune evasion in humans. J. Exp. Med. *211*, 2331–2339.

Payne, A.S., Ishii, K., Kacir, S., Lin, C., Li, H., Hanakawa, Y., Tsunoda, K., Amagai, M., Stanley, J.R., and Siegel, D.L. (2005). Genetic and functional characterization of human pemphigus vulgaris monoclonal autoantibodies isolated by phage display. J. Clin. Invest. *115*, 888–899.

Pinna, D., Corti, D., Jarrossay, D., Sallusto, F., and Lanzavecchia, A. (2009). Clonal dissection of the human memory B-cell repertoire following infection and vaccination. Eur. J. Immunol. *39*, 1260–1270.

Priyamvada, L., Cho, A., Onlamoon, N., Zheng, N.Y., Huang, M., Kovalenkov, Y., Chokephaibulkit, K., Angkasekwinai, N., Pattanapanyasat, K., Ahmed, R., et al. (2016). B Cell Responses during Secondary Dengue Virus Infection Are Dominated by Highly Cross-Reactive, Memory-Derived Plasmablasts. J. Virol. *90*, 5574–5585.

Pugh-Bernard, A.E., Silverman, G.J., Cappione, A.J., Villano, M.E., Ryan, D.H., Insel, R.A., and Sanz, I. (2001). Regulation of inherently autoreactive VH4-34 B cells in the maintenance of human B cell tolerance. J. Clin. Invest. *108*, 1061–1070.

Qian, Y., Diaz, L.A., Ye, J., and Clarke, S.H. (2007). Dissecting the anti-desmoglein autoreactive B cell repertoire in pemphigus vulgaris patients. J. Immunol. *178*, 5982–5990.

Qian, Y., Jeong, J.S., Maldonado, M., Valenzuela, J.G., Gomes, R., Teixeira, C., Evangelista, F., Qaqish, B., Aoki, V., Hans, G., Jr., et al. (2012). Cutting Edge: Brazilian pemphigus foliaceus anti-desmoglein 1 autoantibodies cross-react with sand fly salivary LJM11 antigen. J. Immunol. *189*, 1535–1539.

Robert-Pachot, M., Desbos, A., Moreira, A., Becchi, M., Tebib, J., Bonnin, M., Aitsiselmi, T., Bienvenu, J., and Fabien, N. (2007). A new target for autoantibodies in patients with rheumatoid arthritis. Ann. N Y Acad. Sci. *1108*, 382–391.

Saadoun, D., Terrier, B., Bannock, J., Vazquez, T., Massad, C., Kang, I., Joly, F., Rosenzwajg, M., Sene, D., Benech, P., et al. (2013). Expansion of autoreactive unresponsive CD21-/low B cells in Sjögren's syndrome-associated lymphoproliferation. Arthritis Rheum. *65*, 1085–1096.

Saito, M., Stahley, S.N., Caughman, C.Y., Mao, X., Tucker, D.K., Payne, A.S., Amagai, M., and Kowalczyk, A.P. (2012). Signaling dependent and independent mechanisms in pemphigus vulgaris blister formation. PLoS ONE *7*, e50696.

Sherer, Y., Gorstein, A., Fritzler, M.J., and Shoenfeld, Y. (2004). Autoantibody explosion in systemic lupus erythematosus: more than 100 different antibodies found in SLE patients. Semin. Arthritis Rheum. *34*, 501–537.

Smith, K., Garman, L., Wrammert, J., Zheng, N.Y., Capra, J.D., Ahmed, R., and Wilson, P.C. (2009). Rapid generation of fully human monoclonal antibodies specific to a vaccinating antigen. Nat. Protoc. *4*, 372–384.

Spaeth, S., Riechers, R., Borradori, L., Zillikens, D., Büdinger, L., and Hertl, M. (2001). IgG, IgA and IgE autoantibodies against the ectodomain of desmoglein 3 in active pemphigus vulgaris. Br. J. Dermatol. *144*, 1183–1188.

Stahley, S.N., Saito, M., Faundez, V., Koval, M., Mattheyses, A.L., and Kowalczyk, A.P. (2014). Desmosome assembly and disassembly are membrane raft-dependent. PLoS ONE *9*, e87809.

Stahley, S.N., Warren, M.F., Feldman, R.J., Swerlick, R.A., Mattheyses, A.L., and Kowalczyk, A.P. (2016). Super-Resolution Microscopy Reveals Altered Desmosomal Protein Organization in Tissue from Patients with Pemphigus Vulgaris. J. Invest. Dermatol. *136*, 59–66.

Taylor, J.J., Pape, K.A., Steach, H.R., and Jenkins, M.K. (2015). Humoral immunity. Apoptosis and antigen affinity limit effector cell differentiation of a single naïve B cell. Science *347*, 784–787.

Tiller, T., Tsuiji, M., Yurasov, S., Velinzon, K., Nussenzweig, M.C., and Wardemann, H. (2007). Autoreactivity in human IgG+ memory B cells. Immunity *26*, 205–213.

Tipton, C.M., Fucile, C.F., Darce, J., Chida, A., Ichikawa, T., Gregoretti, I., Schieferl, S., Hom, J., Jenks, S., Feldman, R.J., et al. (2015). Diversity, cellular origin and autoreactivity of antibody-secreting cell population expansions in acute systemic lupus erythematosus. Nat. Immunol. *16*, 755–765.

Tsunoda, K., Ota, T., Aoki, M., Yamada, T., Nagai, T., Nakagawa, T., Koyasu, S., Nishikawa, T., and Amagai, M. (2003). Induction of pemphigus phenotype by a mouse monoclonal antibody against the amino-terminal adhesive interface of desmoglein 3. J. Immunol. *170*, 2170–2178.

Veldman, C., Stauber, A., Wassmuth, R., Uter, W., Schuler, G., and Hertl, M. (2003). Dichotomy of autoreactive Th1 and Th2 cell responses to desmoglein 3 in patients with pemphigus vulgaris (PV) and healthy carriers of PV-associated HLA class II alleles. J. Immunol. *170*, 635–642.

Vidarsson, G., Dekkers, G., and Rispens, T. (2014). IgG subclasses and allotypes: from structure to effector functions. Front. Immunol. *5*, 520.

Witte, M., Zillikens, D., and Schmidt, E. (2018). Diagnosis of Autoimmune Blistering Diseases. Front. Med. (Lausanne) *5*, 296.

Wrammert, J., Smith, K., Miller, J., Langley, W.A., Kokko, K., Larsen, C., Zheng, N.Y., Mays, I., Garman, L., Helms, C., et al. (2008). Rapid cloning of high-affinity human monoclonal antibodies against influenza virus. Nature *453*, 667–671.

Wrammert, J., Koutsonanos, D., Li, G.M., Edupuganti, S., Sui, J., Morrissey, M., McCausland, M., Skountzou, I., Hornig, M., Lipkin, W.I., et al. (2011). Broadly cross-reactive antibodies dominate the human B cell response against 2009 pandemic H1N1 influenza virus infection. J. Exp. Med. *208*, 181–193.

Wrammert, J., Onlamoon, N., Akondy, R.S., Perng, G.C., Polsrila, K., Chandele, A., Kwissa, M., Pulendran, B., Wilson, P.C., Wittawatmongkol, O., et al. (2012). Rapid and massive virus-specific plasmablast responses during acute dengue virus infection in humans. J. Virol. *86*, 2911–2918.

Wu, Y., Jin, X., Harrison, O., Shapiro, L., Honig, B.H., and Ben-Shaul, A. (2010). Cooperativity between trans and cis interactions in cadherin-mediated junction formation. Proc. Natl. Acad. Sci. USA *107*, 17592–17597.

Yamagami, J., Payne, A.S., Kacir, S., Ishii, K., Siegel, D.L., and Stanley, J.R. (2010). Homologous regions of autoantibody heavy chain complementarity-determining region 3 (H-CDR3) in patients with pemphigus cause pathogenicity. J. Clin. Invest. *120*, 4111–4117.

Yeh, S.W., Cavacini, L.A., Bhol, K.C., Lin, M.S., Kumar, M., Duval, M., Posner, M.R., and Ahmed, A.R. (2006). Pathogenic human monoclonal antibody against desmoglein 3. Clin. Immunol. *120*, 68–75.

Yuan, H., Zhou, S., Liu, Z., Cong, W., Fei, X., Zeng, W., Zhu, H., Xu, R., Wang, Y., Zheng, J., and Pan, M. (2017). Pivotal Role of Lesional and Perilesional T/B Lymphocytes in Pemphigus Pathogenesis. J. Invest. Dermatol. *137*, 2362–2370.

STAR★METHODS

KEY RESOURCES TABLE

REAGENT or RESOURCE	SOURCE	IDENTIFIER
Antibodies		
Peroxidase-conjugated goat anti-human IgG	Jackson ImmunoResearch	Cat# 109-036-098; RRID: AB_2337596
Polyvalent goat anti-human Ig	Rockland	Cat# 609-101-130; RRID: AB_219572
Biotinylated donkey anti-human IgG	Jackson ImmunoResearch	Cat# 709-066-098; RRID: AB_2340509
CD3-AF700 (HIT3a)	Biolegend	Cat# 300324; RRID: AB_493739
CD14-AF700 (61D3)	eBioscience	Cat# 56-0149-42; RRID: AB_2574497
CD16-AF700 (CB16)	eBioscience	Cat# 56-0168-42; RRID: AB_2574499
CD20-V450 (L27)	BD	Cat# 561163; RRID: AB_10563614
CD71-FITC (CY1G4)	Biolegend	Cat# 334104; RRID: AB_2201482
IgD-PECy7 (IA6-2)	Biolegend	Cat# 348210; RRID: AB_10680462
CD19-PE (HIB19)	BD	Cat# 302208; RRID: AB_314238
CD27-PerCPCy5.5 (O323)	Biolegend	Cat# 302820; RRID: AB_2073318
Anti-Histag APC	R&D Systems	Cat# IC050A; RRID: AB_10718109
CD19-FITC (HIB19)	BD	Cat# 555412; RRID: AB_395812
CD24-PerCPCy5.5 (ML5)	Biolegend	Cat# 311116; RRID: AB_10960741
CD3-PacBlue (SP34-2)	BD	Cat# 558124; RRID: AB_397044
CD38-PE (HIT2)	BD	Cat# 555460; RRID: AB_395853
CD20-PECY7 (L27)	BD	Cat# 335793; RRID: AB_399971
IgD-PECy7 (IA6-2)	Biolegend	Cat# 348210; RRID: AB_10680462
CD27-APC (O323)	eBioscience	Cat# 17-0279-42; RRID: AB_10671130
AK23	Tsunoda et. al., 2003	Kowalczyk lab
EM4C04	Wrammert et. al., 2011	Wrammert lab
HRP-conjugated goat anti-Etag antibody	Abcam	Cat# ab19400; RRID: AB_732045
Peroxidase-conjugated Mouse anti-human IgG1	Molecular Probe	Cat# MH1715; RRID: AB_10374315
Peroxidase-conjugated Mouse anti-human IgG2	Molecular Probe	Cat# MH1722; RRID: AB_10376169
Peroxidase-conjugated Mouse anti-human IgG3	Molecular Probe	Cat# MH1732; RRID: AB_2539713
Peroxidase-conjugated Mouse anti-human IgG4	Molecular Probe	Cat# MH1742; RRID: AB_2539714
Peroxidase-conjugated goat anti-human IgA	Jackson ImmunoResearch	Cat# 109-036-011; RRID: AB_2337592
Peroxidase conjugated goat anti-mouse IgG	Kirkegaard & Perry Lab, Inc.	Cat# 074-1806
Goat anti-human IgG, cross-adsorbed	Molecular Probes	Cat# A-21433; RRID: AB_2535854
Goat anti-human IgG-AF488, cross-adsorbed	LifeTechnologies	Cat# A-11013; RRID: AB_141360
Bacterial and Virus Strains		
XL10-Gold Ultracompetent E. Coli	Agilent Technologies	Cat# 200314
Chemicals, Peptides, and Recombinant Proteins		
Live/Dead Aqua	ThermoScientific	Cat# L34957
Streptavidin-APC	LifeTechnologies	Cat# SA1005
rDsg3	Eming et. al., 2014	Hertl lab, or Euroimmun
rDsg1	Eming et. al., 2014	Hertl lab, or Euroimmun
rCTxB	List Labs	Cat# 103B
rHA-antigen(A/California/7/2009)	eEnzyme	Cat# IA-SW-12P
R848	Invivogen	Cat# TLR1-R848
Rhu-IL2	Biolegend	Cat# 589102
Avidin-D horseradish peroxidase conjugate	Vector Laboratories	Cat# A-2004
RNase Inhibitor	Promega	Cat# N2111

(*Continued on next page*)

Continued

REAGENT or RESOURCE	SOURCE	IDENTIFIER
Protein A beads	Pierce	Cat# 20334
Dispase	VWR	Cat# 76176-668
Domain-swapped Dsg3/Dsg2, E-tagged	Ohyama et. al., 2012	Payne lab
Domain-swapped Dsg3/Dsg1, E-tagged	Futei et. al., 2000	Payne lab
Tetanus toxoid	List Biological Laboratories	Cat# 191A
Phusion HF HotStart Polymerase	NEB	Cat# M0535L
Prolong gold with DAPI	Life Technologies	Cat# P36931
Critical Commercial Assays		
Dsg1 & 3 ELISA Test System	MBL International Corporation	Cat# RG-M7593-D
EZ-linked Sulfo-NHS-Biotin	ThermoScientific	Cat# 21425
Sensiscript RT Kit	QIAGEN	Cat# 79236
HotStart Taq Plus MasterMix	QIAGEN	Cat# 203645
DNA Purification Kit	QIAGEN	Cat# 28181
ExpiFectamine 293 Transfection Kit	LifeTechnologies	Cat# A14524
Critical Commercial Assays		
Dsg1 & 3 ELISA Test System	MBL International Corporation	Cat# RG-M7593-D
EZ-linked Sulfo-NHS-Biotin	ThermoScientific	Cat# 21425
Sensiscript RT Kit	QIAGEN	Cat# 79236
HotStart Taq Plus MasterMix	QIAGEN	Cat# 203645
DNA Purification Kit	QIAGEN	Cat# 28181
ExpiFectamine 293 Transfection Kit	LifeTechnologies	Cat# A14524
Deposited Data		
mAb sequences	GenBank, NCBI	Accession numbers: GenBank: MN037529 – GenBank: MN037708
Experimental Models: Cell Lines		
Expi293F cells	LifeTechnologies	Cat# A14635
Primary HK cells (neonatal foreskin)	Stahley et al., 2016	Kowalczyk lab
HaCaT cells	Denning et al., 1998	Kowalczyk lab
Oligonucleotides		
R1 PCR Primers	Smith et al., 2009; Wrammert et al., 2008	N/A
R2 PCR Primers	Smith et al., 2009; Wrammert et al., 2008	N/A
R2 PCR primers forward primers, modified with M13R sequence added on 5′ end	Smith et al., 2009; Kauffman et al., 2016	N/A
Cloning PCR primers	Smith et al., 2009; Wrammert et al., 2008	N/A
Nested Primer for IgG4 isotype sequencing – 5′-GGGCTTGTGATCTACGTTGCAG-3′	This paper	N/A
Recombinant DNA		
IgG1 Heavy Chain Expression Vector	Wrammert Lab	GenBank: FJ475055
Kappa Light Chain Expression Vector	Wrammert Lab	GenBank: FJ475056
Lambda Chain Expression Vector	Wrammert Lab	GenBank: FJ517647

LEAD CONTACT AND MATERIALS AVAILABILITY

Further information and requests for resources should be directed to and will be fulfilled by the Lead Contact, Jens Wrammert (jwramme@emory.edu).

EXPERIMENTAL MODEL AND SUBJECT DETAILS

Patients, study design, and approvals

PV patients with active disease were recruited to this study at the time of diagnosis, or were ≥ 6 months post-Rituximab therapy and were currently in either remission or relapse of disease (Table S1). Written informed consent was obtained from all participants. This

study was approved by the Emory Institutional Review Board (#IRB00054860). Demographically matched healthy volunteers were recruited from staff and students at Emory University. Additional information regarding gender and age of enrollees are reported in Table S1. Exclusion criteria included any condition that, in the opinion of the investigator, would place the subject at risk of injury from participation. An additional blood sample had been collected for patient ISD068, 15-months prior to disease diagnosis. The patient recalled participation in another research study prior to development of PV. Having had signed a consent form for this previous study (#IRB00009560), allowing for the use of banked samples for future testing, the patient agreed to pursue additional studies using the pre-clinical sample to determine if markers of autoimmune disease were present in this earlier blood draw.

Cell culture

Primary cell lines of human keratinocytes (HKs) and immortalized cell line of human keratinocytes (Denning et al., 1998) were both generously provided by Dr. Andrew Kowalczyk (Emory University). HKs were isolated from neonatal foreskin, as previously described (Calkins et al., 2006). and grown in keratinocyte growth media (Lonza) supplemented with Singlequots supplements (Lonza) at 37°C/5% CO_2 with no passaging of cells. HaCaTs (ATCC) were maintained in DMEM supplemented with penicillin/streptomycin, L-glutamine, and 10% FBS at 37°C/5% CO_2.

METHOD DETAILS

PBMC and plasma isolation

Blood samples were collected in sodium citrate CPT tubes (BD Vacutainer) from PV patients or healthy controls. Plasma samples were collected and stored at −80°C. PBMCs were isolated and washed with PBS/2% FBS. Cells were used fresh or were frozen in FBS/10% DMSO. Frozen PBMCs were stored in liquid nitrogen until assayed

ELISA

Anti-Dsg3 and anti-Dsg1 serum autoantibody titers were determined with an ELISA test (MBL International Corporation). Patient plasma diluted 1:100 were tested, and results reported as Units/mL of sera, as determined by negative and positive calibrators according to manufacturer’s instructions.

To determine specificity of patient-derived mAbs, ELISA plates (Thermo Scientific) were coated with 2 μg/mL of rDsg3 or rDsg1 (EuroImmun) diluted in PBS with 1 mM $CaCl_2$ and incubated overnight at 4°C. Plates were washed with wash buffer (PBS/0.5% Tween20/1 mM $CaCl_2$) and blocked with ELISA buffer (PBS/1% BSA/0.2% Tween20/1 mM $CaCl_2$). mAbs were added to the plates in a serial dilution series starting at 5 μg/mL. Plates were washed with wash buffer and incubated with peroxidase-conjugated goat anti-human IgG (Jackson ImmunoResearch) at room temperature for 90 min. Plates were washed with wash buffer, followed by a wash with PBS, and then developed using 1 mg/mL o-phenylenediamine (OPD) diluted in 0.05 M phosphate-citrate buffer, supplemented with 0.012% H_2O_2. Reactions were stopped with 1 M HCl, and absorbance was immediately measured at 490 nM. Additional ELISA assays were performed by coating plates with 1 μg/mL recombinant protein antigens CtxB (Kauffman et al., 2016) and influenza HA-antigen (A/California/7/2009, eEnzyme). Minimum effective concentration was determined as the minimal concentration of mAb at which an OD signal was detectable 3-fold above the background signal measured by a negative control.

To determine serum titers against tetanus-toxoid protein, ELISA plates (ThermoScientific) were coated with 0.5 μg/mL of recombinant Tetanus toxoid (List Biological Laboratories) diluted in PBS and incubated overnight at 4°C. Plates were washed with wash buffer (PBS/0.5% Tween20) and blocked with ELISA buffer (PBS/1% BSA/0.2% Tween20). Plasma was added to the plates at a single dilution of 1:200. Plates were washed with wash buffer and incubated with peroxidase-conjugated goat anti-human IgG (Jackson ImmunoResearch) at room temperature for 90 min. Plates were washed with wash buffer, followed by a wash with PBS, and then developed using 1 mg/mL o-phenylenediamine (OPD) diluted in 0.05 M phosphate-citrate buffer, supplemented with 0.012% hydrogen peroxide. Plates were immediately read at an absorbance of 450 nM, with read settings set to record in 15 s intervals over the course of 300 s.

Memory B cell assay

Dsg-specific memory B cells were measured using a polyclonal stimulation assay, essentially as previously described (Pinna et al., 2009). Briefly, PBMCs were cultured at $1X10^6$ cells/mL in RPMI supplemented with penicillin/streptomycin, L-glutamine, 10% FBS, a 50 μM of beta-mercapto-ethanol (Sigma) (R10) containing R848 (1 μg/mL, Invivogen) and recombinant human-IL2 (10 ng/mL, Biolegend) for 3 days at 37°C. Total and Dsg3-specific IgG-secreting cells were quantified by ELISPOT assay, in which a 96-well ELISPOT filter plates (Millipore) were coated overnight with rDsg3 antigen (3 μg/mL, provided by the Hertl Lab), rDsg1 antigen (3 μg/mL, EuroImmun), or polyvalent goat anti-human Ig (25 μg/mL, Rockland) in PBS/1 mM $CaCl_{2.}$ Plates were washed and blocked by incubation with R10 at 37°C for 2 h. PBMCs were added to the plates in a dilution series and incubated for 5 hs at 37°C. Plates were washed with PBS, followed by PBS/0.05% Tween, and incubated with biotinylated anti-human IgG antibody (Invitrogen) at room temperature for 90 min. After washing, plates were incubated with an avidin-D horseradish peroxidase conjugate (Vector laboratories) and developed using 3-amino-9-ethyl-carbazole substrate (Sigma). Plates were scanned and analyzed using an automated ELISPOT counter (CTL, Cellular Technologies).

Flow cytometry analysis and single-cell sorting

Immunophenotyping of circulating B cell subpopulations was performed on previously frozen PBMCs stained with the following, appropriately titrated mAbs: Live/Dead Aqua (ThermoScientific), CD3-AF700 (Biolegend, clone HIT3a), CD14-AF700 (eBioscience, clone 61D3), CD16-AF700 (eBioscience, clone CB16), CD20-V450 (BD, clone L27), CD71-FITC (Biolegend, CY1G4), IgD-PECy7 (Biolegend, clone IA6-2), CD19-PE (BD, clone HIB19), CD27-PerCPCy5.5 (Biolegend, clone O323). Antigen-specific cells were detected using His-tagged rDsg3 (5 μg/mL, provided by the Hertl lab) and a secondary anti-HisTag-APC (R&D system). Influenza HA-specific cells were detected by staining with HA antigen (A/California/7/2009, eEnzyme) which was biotinylated (EZ-Link Sulfo-NHS-Biotin, ThermoFisher) and detected with SA-APC (Life Technologies). A minimum of 1,000,000 lymphocytes were acquired on a BD LSRII flow cytometer, and analyzed using FlowJo software. Dsg3-specific MBCs were single-cell sorted into 96-well PCR plates containing hypotonic catch buffer with RNase inhibitor (Promega) supplemented with 0.2% Triton x-100 (Sigma) using a FACSAriaII, and were frozen immediately on dry ice and stored at −80°C, as previously described (Wrammert et al., 2008).

Additional immunophenotyping of circulating plasmablasts B cells was performed on fresh whole blood stained with the following mAbs, appropriately titrated: CD19-FITC (BD, clone HIB19), CD24-PerCPCy5.5 (Biolegend, clone ML5), CD3-PacificBlue (BD, clone SP34-2), CD38-PE (BD, clone HIT2), CD20-PECy7 (BD, clone L27), IgD-PECy7 (Biolegend, clone IA6-2), and CD27-APC (eBiosciences, clone O323), followed by lysis of erythrocytes (BD FACS lysis solution). 100,000 events were acquired on a BD LSRII flow cytometer, and analyzed using FlowJo software.

Plasmablast ELISPOT assay

ELISPOT was performed to enumerate Dsg3-specific plasmablasts present in PBMC samples. 96-well ELISPOT assay filter plates (Millipore) were coated overnight with recombinant Dsg3 antigen (3 μg/mL, provided by Hertl Lab) or polyvalent goat anti-human Ig (50 μg/mL, Rockland) in PBS/ 1mM $CaCl_2$. Plates were washed and blocked by incubation with R10 at 37°C for 2 h. Freshly isolated PBMCs were added to the plates in a dilution series starting at $5x10^5$ cells and incubated overnight at 37°C. Plates were washed with PBS, followed by PBS/0.05% Tween, and then incubated with biotinylated anti-human IgG, IgA, or IgM antibody (Invitrogen) at room temperature for 90 min. After washing, plates were incubated with avidin D-horseradish peroxidase conjugate (Vector laboratories) and developed using 3-amino-9-ethyl-carbazole substrate (Sigma). Plates were scanned and analyzed using an automated ELISPOT counter (CTL, Cellular Technologies).

Subclass-specific ELISA

To confirm isotype specificity of serum Dsg3-specific antibody responses, ELISA plates were coated with 2 μg/mL of rDsg3. Plates were washed and blocked as previously described. Plasma samples from ISD068 and ISD102 were added to the plates in a serial dilution starting at 1:100 dilutions. After a 90-minute incubation at room temperature, plates were washed and incubated with peroxidase-conjugated mouse anti-human IgG1, IgG2, IgG3, or IgG4 subclass specific reagent (Molecular probe) or a peroxidase-conjugated goat anti-human IgG or IgA reagent (Jackson ImmunoResearch) and incubated for an additional 90 min at room temperature. Plates were washed and IgG subclass signal was amplified by an additional incubation with peroxidase conjugated goat-anti mouse IgG (Kirkegaard & Perry Lab, Inc.) for 90 min at room temperature. Plates were then developed and analyzed as described above.

Immunofluorescence staining

For immunofluorescence experiments, HKs were cultured directly on glass slides (Ibidi) until confluent, as described above. Live cells were labeled with monoclonal antibodies at a concentration of 10 μg/ml for 30 min at 4°C. Cells were then fixed on ice using 3.7% paraformaldehyde for 10 min followed by permeabilization in 0.1% triton for 10 min at room temperature. Goat anti-human IgG cross-adsorbed secondary (Life Technologies) was then used prior to mounting with prolong gold containing Dapi (Life Technologies). Widefield images were acquired using a DMRXA2 microscope (Leica, Wetzler, Germany) equipped with a 63X/1.40 NA oil immersion objective. Images were acquired with an ORCA digital camera (Hamamatsu Photonics, Bridgewater, NJ). Analysis was preformed using Fiji ImageJ.

Generation of recombinant monoclonal antibodies

mAbs were generated from single-cell sorted MBCs by single-cell expression cloning, essentially as previously described (Smith et al., 2009; Wrammert et al., 2008). In brief, single-cell cDNA was synthesized from sorted MBCs using gene-specific primers (IDT). Ig heavy chain and light chain (kappa/lambda) rearrangements were amplified by nested PCR (HotStarTaq Plus Master Mix, QIAGEN) using primer cocktails specific for all V gene families and constant domains at a concentration of 200 nM per primer. Sense primers used in the second round of nested PCR were modified by fusing the 5′ end of each primer to the M13R sequence (5′-A ACAGCTATGACCATG-3′) to facilitate subsequent sequencing. Next, another PCR was performed using a high-fidelity DNA polymerase (Phusion Hot Start II, NEB) and V and J gene family specific primers that incorporated Ig chain-specific restriction sites. Variable domains were directionally cloned into human mAb heavy chain (IgG1) and light chain (kappa or lambda) expression vectors (GenBank accession numbers GenBank: FJ475055, GenBank: FJ475056, and Genbank: FJ517647). Variable domains containing internal restriction sites were amplified by a modified PCR containing 50 μM 5-methyl-dCTP (NEB) and 150 μM dCTP. This modification did not affect PCR fidelity and abrogated the truncation of variable domains after restriction digestion. Following vector construction and sequence confirmation, heavy and light chain vectors were transiently co-transfected into Expi293F cells according

manufacturer's instructions (Life Technologies). Antibodies were purified from cell culture supernatants using protein-A conjugated agarose beads (Pierce).

Repertoire analysis

Identification of antibody variable region genes were done as previously described (Cho et al., 2017; Kauffman et al., 2016). V, D, and J genes were identified and analyzed using the Immunogenetics (IMGT) database as a reference. Isotype was determined by comparing the nucleotide sequence of the Fc-domain retained by nested PCR described above to the GenBank database as a reference for subclass specificity (GenBank accession numbers: IgG1 - AH007035.2; IgG2 - AJ250170.1; IgG3 - X03604.1; IgG4 - AF237586.1; IgA1 – AH007035.2; IgA2 – AH005273.2; IgM – X14940.1). To confirm IgG subclasses, a subset of MBCs was re-amplified using the first round of PCR amplification described above using a nested primer (5′-GGGCTTGTGATCTACGTTGCAG-3′) that amplified nucleotide sequences to incorporate additional regions of sufficient subclass dissimilarity and compared to previously reported subclass-specific sequences (Vidarsson et al., 2014). The number of somatic hypermutations in the V_H genes represent the total number of both replacement and silent mutations in the amplicon (FR1 through CDR3) relative to the closest germline sequence matched in the IMGT database. Members of clonal expansions were identified by sequence alignments of rearrangements with matching V and J gene usage, the same CDR3 length, and $\geq$ 80% similar junctional diversity. Germline-reverted antibodies were also generated by comparison to the IMGT database. Fragments of the germline sequence with the appropriate restriction sites were synthesized (Eurofins) and used to generate mAbs as described above.

Keratinocyte Dissociation Assay

HK cultures were seeded into 24-well dishes in keratinocyte growth media (Lonza) as described above. 24-hours after reaching confluency, cultures were switched to low calcium media (KGM containing 50 μM $CaCl_2$) overnight to internalize all desmosomal cadherins. Cultures were switched to high calcium (550 μM $CaCl_2$) media for 3 h, and treated with 10 μg/mL of mAbs for 6 h. The cells were then incubated with dispase (VWR) at 37°C until monolayers were released from plate. Monolayers were transferred to Eppendorf tubes and subjected to mechanical stress by shaking on a cell shaker for one min, and then transferred back into a 24-well plate and fixed with formaldehyde and stained with methyl blue. Fragments were imaged using an imager (CTL, Cellular Technologies), and the number of fragments was determined as the average count determined from three separate images of the same well. Dissociation index (DI) was calculated using the following equation: (1 - [(No. $\text{fragments}_{\text{mAb}}$ – No. $\text{fragments}_{\text{negative control}}$)/(No. $\text{fragments}_{\text{positive control}}$ – No. $\text{fragments}_{\text{negative control}}$)]) x 100, in which the negative control was the influenza-specific mAb EM4C04 (Wrammert et al., 2011) and the positive control was the mouse mAb AK23 (Tsunoda et al., 2003).

Synergy of pathogenic responses detected using the above assay by treating HK cells with titrated amount of antibodies in a 3-fold dilution series starting at a concentration of 10 μg/mL. For synergy panel, 10 μg/mL of three individual antibodies were combined (final concentration 30 μg/mL) and titrated out at 3-fold dilutions.

Domain mapping

Domain mapping experiments were done using domain-swapped Dsg3-Dsg2 and Dsg3-Dsg1 molecules as previously described (Ohyama et al., 2012). Dsg3-Dsg2 extracellular cadherin (EC) domain-swapped constructs (generously provided by Amagai lab, Keio University, Japan). For each construct, one extracellular domain of Dsg2 was substituted for corresponding domain in Dsg3, followed by an E-tag at the C terminus. Plasmid constructs were transfected into 293T cells with jetPRIME (Polypus). The domain-swapped molecules were secreted into the cell culture supernatant, which was centrifuged to remove cell debris and frozen at −80°C. For antibodies not scoring in this assay, Dsg3-Dsg1 EC domain-swapped constructs (provided by Masayuki Amagai) were used for epitope mapping (Futei et al., 2000). These constructs were generated by amplifying a section of Dsg3 that span multiple junctions between several extracellular domains. These sections ($Dsg3^{1-161}$, $Dsg3^{1-258}$, and $Dsg3^{163-566}$) were swapped with the corresponding sections of Dsg1. The constructs were produced by baculovirus expression, as previously described (Ohyama et al., 2012), and stored at −80°C.

1 μg of mAbs was incubated overnight at 4°C with these domain-swapped constructs, followed by incubation with Protein A Agarose (Invitrogen) for 1 h at room temperature. Pull down samples were washed with TBS/1 mM $CaCl_2$, boiled for 5 min at 100°C in Laemmi Sample Buffer (BioRad) and centrifuged. The immune-precipitated proteins were separated by SDS-PAGE, transferred to nitrocellulose membranes (BioRad) and visualized through incubation with HRP-conjugated goat anti-Etag antibody (Abcam) followed by exposure to Digital-ECL substrate solution (Kindle Biosciences). The chemo-luminescence signal was quantified using the *Kwik*Quant Imager (Kindle Biosciences).

HaCaT binding and competition assay

To collect single-cell suspensions, HaCaTs were maintained as described above, and grown to 70%–80% confluency. Cells were washed with PBS, and treated with 0.25% trypsin (Lonza) before quenching reaction with culture media. Cells were washed twice and rested in 4 mL of culture media for 90 min at 37°C. Cells were then washed with PBS, stained with Live/Dead Aqua (ThermoScientific) and then washed with staining buffer (PBS/2% FBS/0.05% sodium azide/1 μM CaCl2) and aliquoted into a

96-well plate. Cells were incubated with 1 μg of mAb for 30 min at 4°C, and then washed and incubated with mouse anti-human IgG-AF488 (Life Technologies). A minimum of 10,000 cells were acquired on a BD LSRII flow cytometer, and analyzed using FlowJo software.

For competition assays, mAbs were biotinylated (EZ-Link Sulfo-NHS-Biotin, ThermoFisher). Only biotinylated-mAbs that provided a signal 1.5-times brighter than the negative control, biotinylated-EM4C04, were used for this assay. Single-cell suspension of HaCaTs were rested as described above. Cells were then washed with PBS, stained with Live/Dead Aqua (ThermoScientific), washed with staining buffer (PBS/2% FBS/0.05% sodium azide/1 μM CaCl2) and aliquoted into a 96-well plate. Cells were incubated with unlabeled competing antibody at 100 μg/mL for one h at 4°C. An equal volume of buffer containing 2 μg/mL biotinylated-mAb was added, and cells were incubated for an additional 30 min at 4°C. Cells were then washed and incubated with appropriately diluted streptavidin-APC reagent (Invitrogen) for 30 min at 4°C. A minimum of 10,000 cells were acquired on a BD LSRII flow cytometer, and analyzed using FlowJo software. Percent inhibition was calculated as: $(1 - [(MFI_{sample} - MFI_{negative\ control})/(MFI_{positive\ control} - MFI_{negative\ control})]) \times 100$, in which the negative control was influenza-specific biotinylated-EM4C04 and the positive control was 1 μg/mL of biotinylated antibody without competitor.

Blocking ELISA

To determine epitope specificity of patient sera, a blocking ELISA was performed by inhibiting the binding of a biotinylated mAb (EZ-Link Sulfo-NHS-Biotin, ThermoFisher) targeting 1 of 5 described immunodominant epitopes (from the competition assay described in Figure 4) at the half-maximal binding concentration with 1:20 diluted sera from PV patients or healthy controls to Dsg3-coated ELISA plates. To determine if the immunodominant epitopes bound to the same epitope as mouse AK23 antibody, we also used a blocking ELISA to inhibit the binding of biotinylated mAbs with a 10-fold molar excess of unlabeled AK23 to Dsg3-coated ELISA plates. Detection was done using avidin-HRP as described for the ELISA assay. The cut-off value was determined by the mean of %inhibition calculated for 9 healthy control sera plus 2 SD. Percent inhibition was calculated as follows: $(1 - [(OD_{samples} - OD_{negative\ control})/(OD_{positive\ control} - OD_{negative\ control})]) \times 100$.

QUANTIFICATION AND STATISTICAL ANALYSIS

Statistical analysis

Data was collected and graphed using GraphPad Prism software. A 1-way ANOVA Kruskal-Wallis test, Mann Whitney U-test, Wilcoxon matched-pairs signed-rank test, or a Spearman correlation was used to determine statistical significance where appropriate. *P values* less than 0.05 (two-sided) were considered statistically significant. Information about the statistics used for each experiment, including sample size, experimental method, and specific statistic test employed, can be found in the relevant figure or figure legends.

DATA AND CODE AVAILABILITY

The accession numbers for the antibodies cloned for this paper is available at GenBank with the accession number: GenBank: MN037529 – GenBank: MN037708.

Cell Reports, Volume *28*

Supplemental Information

Single-Cell Analysis Suggests that Ongoing Affinity Maturation Drives the Emergence of Pemphigus Vulgaris Autoimmune Disease

Alice Cho, Amber L. Caldara, Nina A. Ran, Zach Menne, Robert C. Kauffman, Maurizio Affer, Alexandra Llovet, Carson Norwood, Aaron Scanlan, Grace Mantus, Bridget Bradley, Stephanie Zimmer, Thomas Schmidt, Michael Hertl, Aimee S. Payne, Ron Feldman, Andrew P. Kowalczyk, and Jens Wrammert

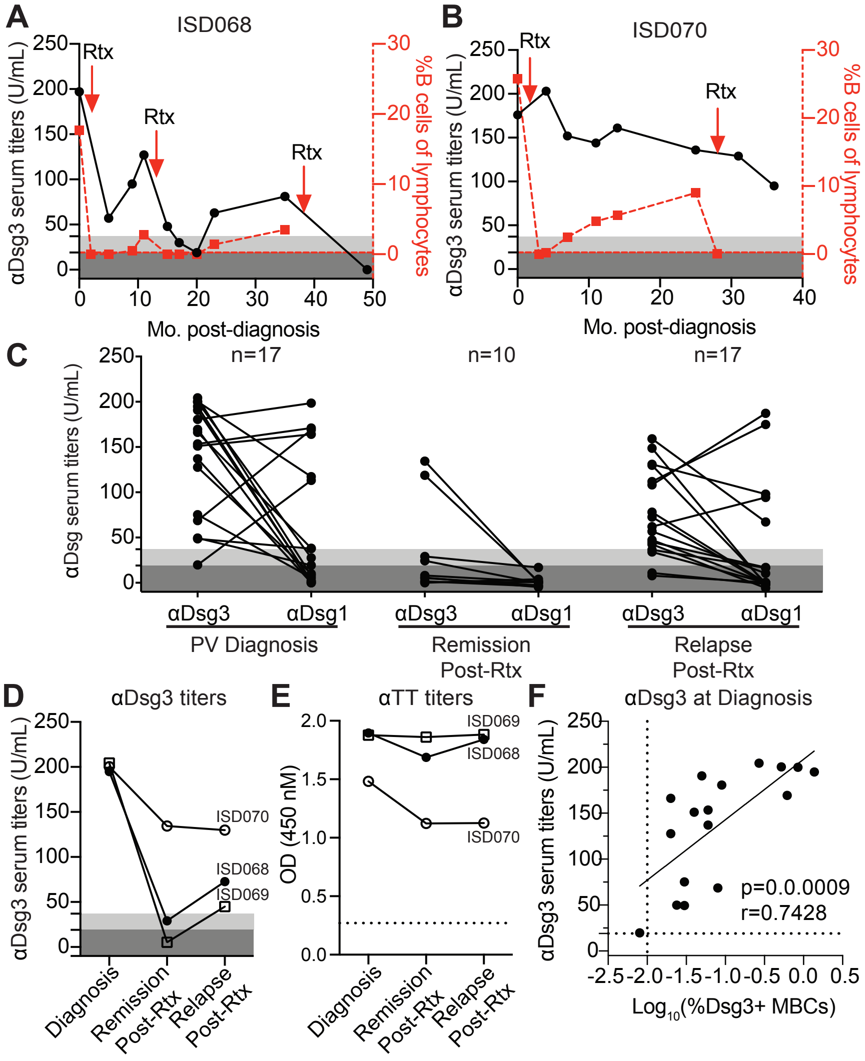

Figure S1. Related to Figure 1. Kinetics of serum antibody titers before and after treatment with B cell depletive therapy. (A) The average kinetics of anti-Dsg3 serum titers (left Y-axis, black line) and peripheral blood CD19+ B cells (right Y-axis, red line) for most PV patients after treatment with Rituximab (Rtx) therapy. Cut-off values for ELISA were determined by manufacturer's suggestion (dark grey = negative, light grey = indeterminate). Red dotted line represents limit of detection for CD19+ B cells in circulation. (B) A smaller number of patients who are in clinical remission do not have a complete decrease of anti-Dsg3 serum titers in response to Rituximab-mediated therapy, despite showing complete depletion of B cells in the periphery. (C) Anti-Dsg1 serum titers were found only in a subset of PV patients, specifically at the time of diagnosis and relapse post-Rtx, but undetectable in patients in remission. When tracking serum titers specifically in PV patients sampled longitudinally at multiple time points (D) anti-Dsg3 serum titers decreased dramatically after Rituximab-treatment while (E) anti-tetanus toxoid (TT) titers did not significantly change over time. Anti-TT titers are shown as the final OD reading of serum diluted at 1:200. Dotted-line represents the cut-off value determined by the background signal in a negative HC serum. Data is representative of two individual experimental repeats. (F) There was a significant positive correlation between frequency of Dsg3-specific MBCs and anti-Dsg3 serum titers. Dotted lines represent cut-off values for either the ELISA or MBC assay. A spearman correlation was used to analyze this data.

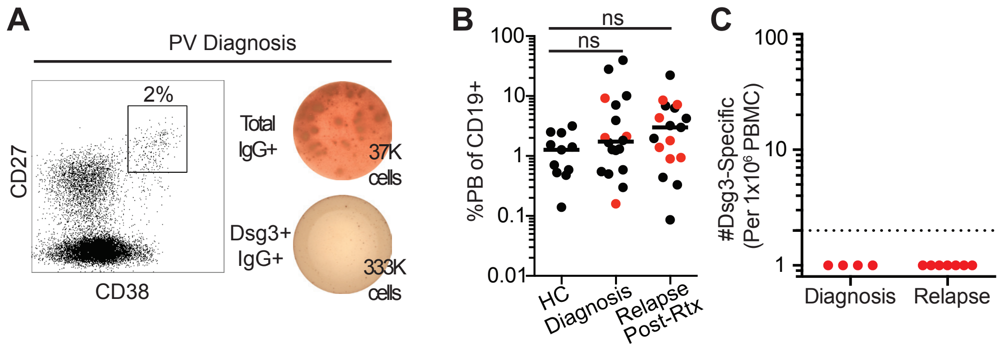

Figure S2. Related to Figure 1. No Dsg3-specific circulating plasmablasts are detected in symptomatic PV patients. (A) A representative flow plot of plasmablasts detected in peripheral blood (gated on CD3-CD19+ lymphocytes) and an ELISPOT assay, showing a single dilution of titrated PBMCs probed for total IgG and Dsg3-specific IgG antibody-secreting cells from a PV patient at the time of diagnosis. (B) Frequency of plasmablasts does not differ between HC and patients at diagnosis or relapse. Red dots represent patients tested by ELISPOT for Dsg3-specific plasmablasts. (C) Absence of detectable Dsg3-specific circulating plasmablasts in patients presenting with active PV disease. Dotted line represents limit of detection of the ELISPOT assay at 3 antibody secreting cells per $1x10^6$ PBMCs. A one-way ANOVA was used to analyze this data.

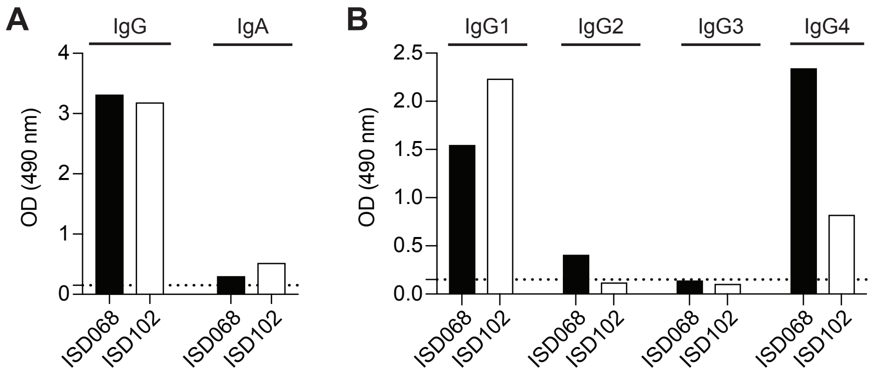

Figure S3. Related to Figure 2. Dsg3-specific serum antibodies were primarily IgG1 and IgG4 isotype. An ELISA was used to determine isotype usage of Dsg3-specific serum antibodies from patients ISD068 and ISD102. Shown is the OD reading of serum diluted at 1:100. (A) Dsg3-specific serum responses are predominantly IgG, although some IgA was detected in both patients. (B) ELISA using IgG subclass reagents show that the Dsg3-specific responses are dominated by IgG1 and IgG4 subclass usage. Little to no IgG2 or IgG3 was detected in either of the patients. Dotted line represents cut-off value determined by the background signal detected in HC serum. Data is representative of two individual experimental repeats.

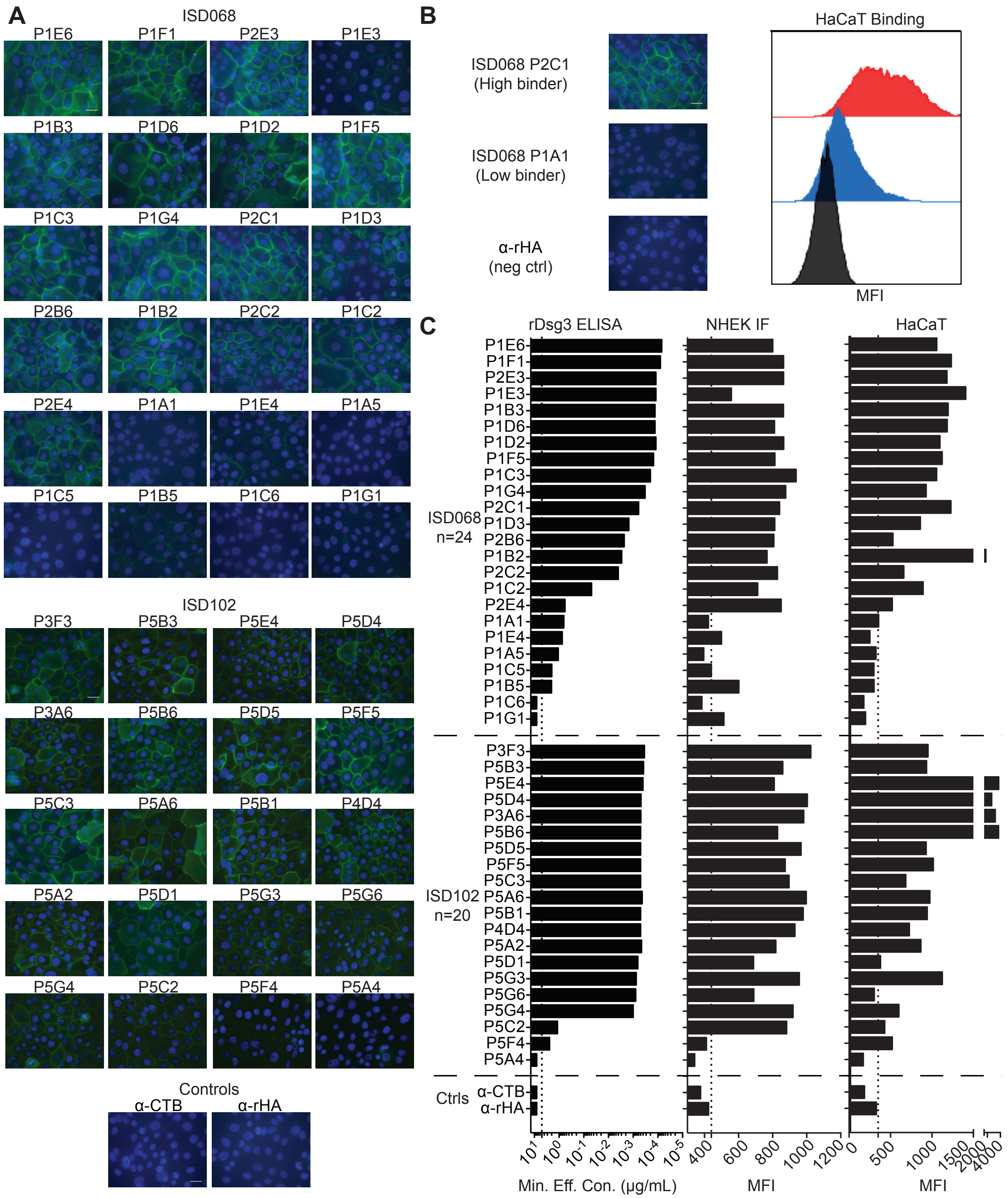

Figure S4. Related to Figure 2. Assessment of mAbs binding to Dsg3 expressed on cell surface. Binding of mAbs to Dsg3 was determined using (A) immunofluorescent staining of HK cells (primary cells line of human keratinocytes) and (B) flow cytometry-based assay of staining HaCaT cells (immortalized cell line of human keratinocytes), shown as histograms. Representative images from panel S4A are used in panel S4B to provide a direct, side-by-side comparison between the two different methods. (C) Summary data comparing binding measured via ELISA, IF, and flow cytometry shows that there is a range of binding activity of mAbs towards Dsg3. Representative data of two individual experimental repeats is shown. White scale bar in A, 25 μm. Dotted line for MFI represents cut-off value for a negative signal.

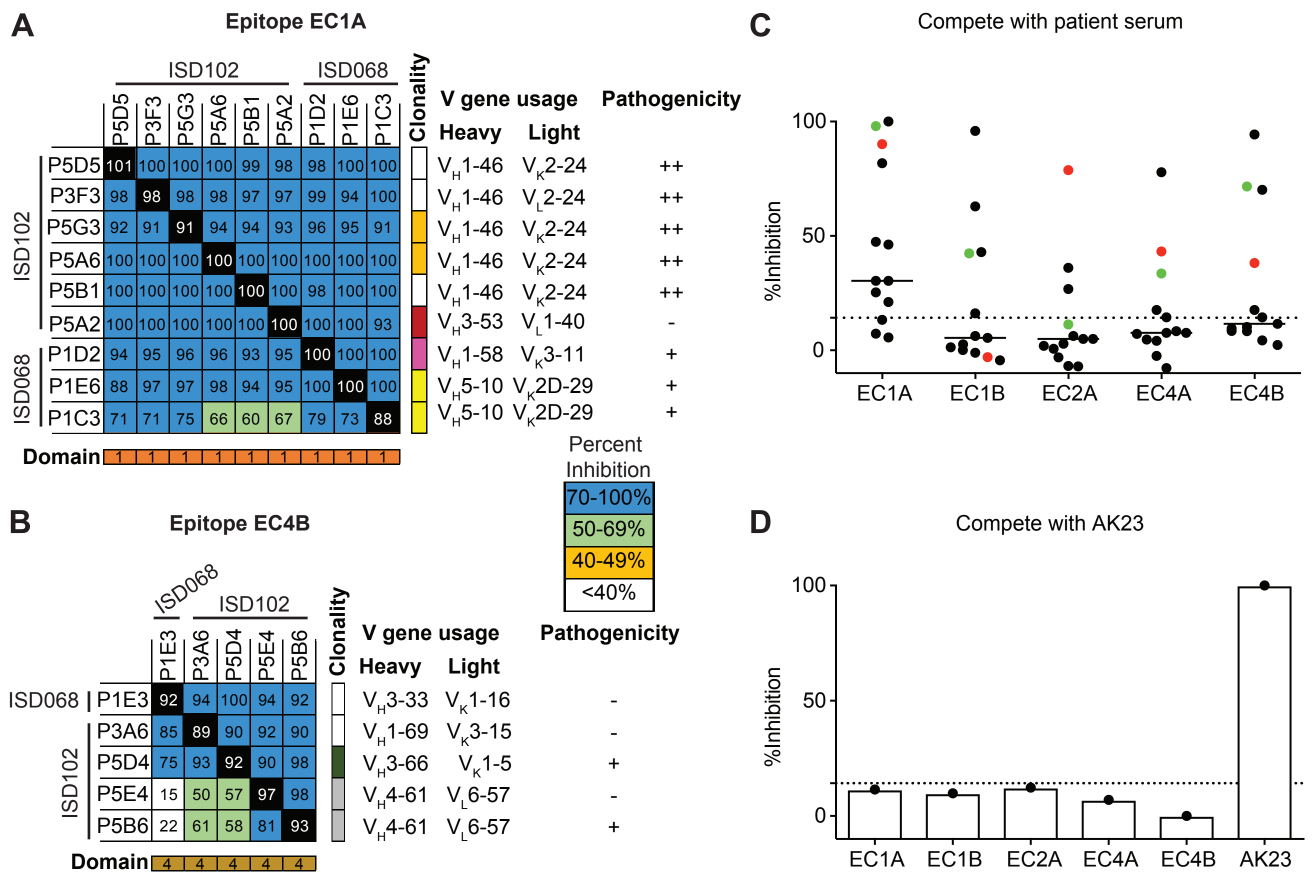

Figure S5. Related to Figure 4. Similar sterically-distinct epitopes detected by flow cytometry-based blocking assay were representative of other PV patients at diagnosis. The flow-based blocking assay was also used to define overlapping epitopes between patients ISD068 and ISD102. Antibodies targeting (A) epitope EC1A and (B) epitope EC4B could be detected in both patients. (C) A blocking ELISA was performed by using 13 PV serum from time of diagnosis to block binding of 5 different biotinylated mAbs to Dsg3, each mAb representative of the 5 detected epitopes described in Figure 4. While EC1A epitope was most commonly detected in all patients, the other 4 epitopes were targeted as well. Red dot: patient ISD068; Green dot: patient ISD102. (D) A blocking ELISA was also used to determine if the 5 detected epitopes bound to the same epitope as AK23, an EC1-specific pathogenic mouse-derived mAb (Tsunoda et al., 2003). Interestingly, none of the 5 described epitopes targeted by the human MBC-derived mAbs bound to the same epitopes as the EC1-specific AK23, suggesting that preferred immunodominant epitopes are different for humans mAbs versus mouse mAbs. Representative data of two individual experimental repeats is shown. Dotted line represents cut-off value for positive inhibition, as determined by the mean of inhibition of 9 HC sera plus 2 SD.

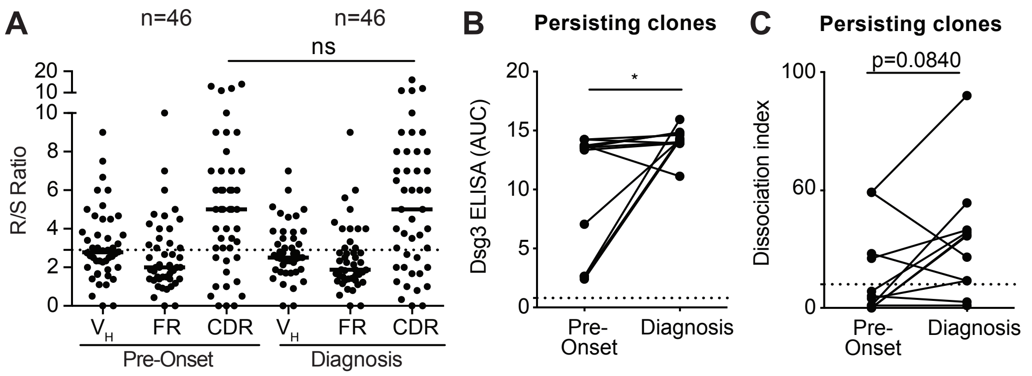

Figure S6. Related to Figure 5. Antigen selection and ongoing affinity maturation of Dsg3-specific memory B cells. (A) R/S ratios were above 2.9 in the CDR at both pre-onset and diagnosis, indicating that antigenic selection is an ongoing process occurring continuously during disease development. When comparing only mAbs derived from persisting clones (MBCs from the same clonal family found at both pre-onset and diagnosis time points), (B) there was a significant increase in relative affinity of mAbs for Dsg3 and (C) a trend towards an increase in pathogenicity from pre-onset to diagnosis. A Mann-Whitney U test or Wilcoxon paired T-test was used where appropriate. * = P≤0.05

A ISD068 Pre-onset

Epitope	mAb	P1C5	P1B2	P2D6	P1F4	P1D5	P1D4	P1D1	P2F2	P1F3	P2G2	P2D3	P2G1	P1G2	P2D2	P1G1	P2D1	P1E3	P2C4	P1B6	P1G3	Clonality	Heavy	Light	Pathogenicity
EC1A	P1C5	90	84	96	94	95	11	84	87	53	0	0	0	0	2	0	0	0	0	5	0		V_H5-10	V_κ2D-29	-
EC1A	P1B2	85	92	85	94	88	75	82	58	44	13	0	0	0	8	0	0	0	0	9	0		V_H1-2	V_L1-47	-
EC1A	P2D6	66	90	96	93	91	92	81	83	49	0	6	21	16	60	41	10	29	13	38	18		V_H1-58	V_κ3-11	+
EC1A	P1F4	24	62	79	96	96	60	62	53	43	3	6	4	10	29	9	13	12	5	21	0		V_H5-51	V_L3-9	+
EC1A	P1D5	30	86	85	95	96	77	72	78	63	14	19	22	18	47	20	25	31	7	61	6		V_H1-2	V_L1-51	+
EC2A	P1D4	17	93	101	96	97	98	114	93	47	15	21	9	62	94	96	95	92	95	95	13		V_H1-18	V_κ1-9	-
EC2A	P1D1	11	54	55	52	87	91	97	96	92	78	65	88	70	86	81	86	84	82	86	0		V_H1-69	V_κ3-11	+
EC2A	P2F2	19	22	12	24	39	84	88	96	57	57	26	78	51	95	84	94	77	97	95	4		V_H1-2	V_κ3-15	-
EC2A	P1F3	0	0	0	0	54	0	89	94	82	87	84	91	88	89	88	95	85	88	92	0		V_H4-39	V_κ1-5	-
EC2A	P2G2	0	0	3	13	50	0	92	90	85	96	92	97	86	97	96	89	93	88	91	5		V_H4-39	V_κ1-5	+
EC2A	P2D3	0	0	14	12	56	13	98	95	95	99	97	98	87	87	95	89	97	93	91	13		V_H4-39	V_κ1-5	-
EC2A	P2G1	12	30	1	9	30	14	88	96	75	83	48	98	62	90	90	86	86	93	95	8		V_H4-39	V_κ3-15	+
EC2A	P1G2	0	0	8	69	88	93	107	96	94	82	90	90	86	85	93	95	85	93	93	3		V_H4-4	V_κ3-20	-
EC2A	P2D2	12	52	22	36	62	87	99	94	87	83	83	95	88	91	93	93	91	95	96	10		V_H4-4	V_κ3-20	-
EC2A	P1G1	15	17	4	47	75	90	99	95	87	91	74	96	83	95	94	96	96	95	95	0		V_H4-4	V_κ3-20	-
EC2A	P2D1	23	21	20	31	68	91	95	94	89	83	82	95	75	93	94	94	86	94	95	18		V_H1-2	V_κ3-15	-
EC2A	P1E3	0	5	3	35	67	95	107	95	92	93	78	96	85	95	95	97	95	98	96	12		V_H1-2	V_κ3-15	-
EC2A	P2C4	15	23	15	24	49	86	92	93	63	59	10	92	22	96	86	90	79	95	92	4		V_H4-59	V_κ3-15	-
EC2A	P1B6	15	20	13	21	49	81	86	96	24	8	6	93	17	89	82	89	74	93	96	2		V_H1-2	V_κ3-20	+
Unknown	P1G3	0	11	10	0	0	19	7	4	3	11	9	11	0	0	8	0	0	0	0	100		V_H5-10	V_κ1-33	+

B ISD068 Diagnosis

Epitope	mAb	P1D2	P1E6	P1C3	P1C2	P1A1	P2E3	P1D6	P1D3	P2C2	P1A2	P1G4	P1B3	P1F5	P1F1	P1B2	P2E4	P2B6	P2C1	P1E3	Clonality	Heavy	Light	Pathogenicity
EC1A	P1D2	100	100	100	91	64	60	57	48	49	61	1	8	2	6	11	25	4	18	2		V_H1-58	V_κ3-11	+
EC1A	P1E6	100	100	100	94	11	7	4	1	9	17	0	3	3	1	10	9	9	9	2		V_H5-10	V_κ2D-29	+
EC1A	P1C3	79	73	88	69	1	12	7	13	20	7	2	14	3	3	17	19	4	17	8		V_H5-10	V_κ2D-29	+
EC2A	P1C2	41	22	46	100	87	100	94	95	79	102	100	97	100	99	96	83	7	20	13		V_H4-39	V_κ3-15	+
EC2A	P1A1	64	35	49	97	96	97	98	96	92	98	97	96	98	97	96	94	92	1	6		V_H4-34	V_κ3-20	-
EC2A	P2E3	15	10	14	88	87	99	100	86	70	96	86	97	87	95	76	42	9	11	6		V_H1-2	V_κ3-11	+
EC2A	P1D6	9	25	27	87	92	99	99	79	70	97	78	98	79	87	62	45	8	16	7		V_H1-2	V_κ3-15	-
EC2A	P1D3	47	18	13	92	100	100	96	92	92	84	99	97	97	95	95	87	74	7	4		V_H1-2	V_κ3-11	+
EC2A	P2C2	43	33	20	100	87	100	100	95	100	99	100	96	100	98	100	96	100	15	0		V_H1-2	V_κ3-11	-
EC2A	P1A2	26	23	28	99	97	98	98	90	63	99	98	98	98	95	85	50	36	13	3		V_H4-59	V_κ3-11	+
EC2A	P1G4	1	0	2	84	98	99	93	87	78	87	95	99	100	97	79	55	47	0	0		V_H4-39	V_κ1-5	-
EC2A	P1B3	12	16	11	91	94	100	97	92	86	81	99	100	100	93	77	76	62	10	9		V_H4-39	V_κ3-15	+
EC2A	P1F5	6	6	0	88	83	100	98	86	77	92	99	100	100	94	72	41	41	0	6		V_H4-39	V_κ3-15	+
EC2A	P1F1	12	12	12	86	94	100	100	85	80	96	100	100	100	100	72	65	45	6	15		V_H4-39	V_κ1-5	+
EC2A	P1B2	5	1	21	72	82	88	100	100	80	96	91	100	100	94	100	56	75	27	10		V_H4-39	V_κ1-5	+
EC2A	P2E4	0	0	1	100	81	100	89	100	95	101	100	100	100	100	98	95	100	0	12		V_H4-39	V_κ1-5	-
EC2A	P2B6	0	0	0	96	97	97	96	97	94	97	96	97	91	96	96	96	95	0	0		V_H4-39	V_κ1-5	+
EC4A	P2C1	3	10	6	28	5	8	9	6	10	8	12	11	13	14	14	16	12	98	98		V_H4-39	V_κ1-5	+
EC4B	P1E3	0	0	0	0	0	12	7	0	0	2	11	9	13	15	39	0	5	20	92		V_H3-33	V_κ1-16	-
	Domain	1	1	1	2	2	2	2	2	2	2	2	2	2	2	2	2		4	4				

Percent Inhibition
70-100%
50-69%
40-49%
<40%

Figure S7. Related to Figure 5. Similar sterically-distinct epitopes are detected at pre-onset and diagnosis time points. A flow-based blocking assay was used to detect sterically-distinct epitopes of antibodies from patient ISD068 derived from (A) pre-onset and (B) diagnosis time points. Similar frequencies of EC1A and EC2A epitopes could be detected at pre-diagnosis, as well as diagnosis time point, suggesting that no epitope spreading has occurred between the two time points. Designated name of the epitope bound by antibodies is specified on the left of the chart where appropriate. Domain-specificity of mAbs derived from the diagnosis time point is illustrated at the bottom of the chart (Orange: EC1; Grey: EC2; Gold: EC4; White: Interdomain). mAbs in the same clonal family are represented on the right of the chart. VH and VL usage and pathogenicity are listed on the right of the panel. Pathogenicity was determined by using the following cut-off values of the dissociation index number: - = DI≤10; + = 10<DI≤100.

TABLE S1. Related to Figure 1. Characteristics of subjects at time of enrollment

GROUP	Patient ID	Gender	Age	Disease	% B cells of lymphocytes	Medications (mg/d)	Mo. Post recent Rtx	No. of previous Rtx	PDAI[a]	Dsg3 Titer[c]	Dsg1 titer[d]
PV patients DIAGNOSIS (n=17)	ISD001	M	48	mucocutaneous	10.9	Prednisone (30)			12	20	133
	ISD003	M	26	mucosal	8.2	Prednisone (20)			11	137	neg
	ISD020	M	48	mucocutaneous	6.3	Prednisone (60)			15	69	169
	ISD058	F	43	mucosal	15.8	Prednisone (30)			15	50	neg
	ISD061	F	54	mucosal	8.8	Prednisone (60)			6	191	neg
	ISD066	M	52	mucocutaneous	1.8	Prednisone (30)			36	181	199
	ISD068[f]	***M***	***60***	***mucocutaneous***	***4.3***	***Prednisone (40)***			***8***	***195***	***neg***
	ISD069	M	38	mucocutaneous	13.7	Prednisone (30)			55	204	neg
	ISD070	M	48	mucocutaneous	11.8	None			0	200	117
	ISD082	M	54	mucocutaneous	9.4	Prednisone (80)			5	49	38
	ISD086	F	31	mucocutaneous	10.0	Prednisone (60)			4	128	neg
	ISD091	F	45	mucocutaneous	5.7	Prednisone (60)			64	153	171
	ISD100	M	60	mucocutaneous	10.1	Prednisone (20)			20	169	neg
	ISD102[f]	***F***	***45***	***mucocutaneous***	***6.5***	***Prednisone (10)***			***36***	***200***	***neg***
	ISD106	F	51	mucocutaneous	11.5	Prednisone (20)			4	75	neg
	ISD109	F	48	mucocutaneous	6.8	Prednisone (80)			20	166	38
	ISD112	M	42	mucocutaneous	7.5	Data unavailable			30[b]	151	164
PV patients REMISSION[e] (post-Rtx) (n=10)	R15	F	45	mucosal	10.8	None	20	0	1	neg	neg
	R15-06	M	41	mucosal	8.8	Cellcept (500)	19	2	0	120	neg
	ISD002	F	48	mucosal	2.3	None	6	1	0	neg	neg
	ISD025	M	58	mucosal	7.8	Prednisone (2.5)	22	0	0	neg	neg
	ISD060	F	47	mucosal	13.2	None	17	0	2	24	neg
	ISD068	M	63	mucocutaneous	3.2	None	11	2	0	29	neg
	ISD069	M	39	mucocutaneous	7.6	None	13	0	3	neg	neg
	ISD070	M	51	mucocutaneous	8.2	None	29	0	1	135	neg
	ISD072	F	51	mucocutaneous	8.9	None	12	0	0	neg	neg
	ISD082	M	55	mucocutaneous	5.5	None	13	0	0	neg	neg
PV patients RELAPSE[e]	ISD005	M	64	mucocutaneous	3.2	Prednisone (40)	77	0	12	131	98
	ISD031	M	77	mucocutaneous	5.4	None	27	1	1	neg	neg
	ISD038	M	66	mucocutaneous	5.6	None	13	0	15	112	175

PV patients RELAPSE[e] (cont'd) (post-Rtx) (n=17)	ISD060	F	48	mucosal	11.9	None	22	0	5	43	neg
	ISD061	F	55	mucosal	5.5	None	11	0	0	34	neg
	ISD063	F	61	mucocutaneous	10.6	None	8	0	12	149	neg
	ISD068	M	62	mucocutaneous	3.1	None	13	1	2	73	neg
	ISD069	M	41	mucocutaneous	16	None	20	1	2	45	neg
	ISD070	M	51	mucocutaneous	7.1	None	36	0	7	130	neg
	ISD072	F	52	mucocutaneous	13.1	None	21	1	0	108	187
	ISD074	M	49	mucocutaneous	9.6	None	12	3	15	62	94
	ISD084	F	50	mucocutaneous	4.3	None	9	0	3	neg	neg
	ISD086	F	32	mucocutaneous	9.3	None	17	0	3	159	67
	ISD104	F	50	mucosal	37.2	None	10	0	22	57	neg
	ISD106	F	51	mucocutaneous	7.33	None	8	0	4	47	neg
	ISD110	M	37	mucocutaneous	14.4	None	20	0	2	37	neg
	ISD122	M	63	mucocutaneous	3.7	None	45	4	25	78	neg
Healthy controls (n=11)	HC01	F	48		11.3						
	HC02	F	53		12.3						
	HC08	F	57		8.7						
	HC13	M	40		4.1						
	HC15	F	31		11.5						
	H15-08	F	45		4.9						
	H15-11	F	45		6.6						
	H15-13	F	46		14.9						
	HC421	F	25		5.6						
	HC435	F	27		9.5						
	HC467	M	37		10.0						

[a]Pemphigus Disease Activity Index (According to: Rosenbach M, et al. Reliability and convergent validity of two outcome instruments for pemphigus. *The Journal of investigative dermatology.* 2009;129(10):2404-10.

[b]PDAI score calculated from photos

[c]Dsg3 titers reported in U/mL. Cut-off values determined by manufacturer recommendation: neg (negative) < 19

[d]Dsg1 titers reported in U/mL. Cut-off values determined by manufacturer recommendation: neg (negative) < 18

[e]According to Rosenbach M, et al. Reliability and convergent validity of two outcome instruments for pemphigus. *The Journal of investigative dermatology.* 2009;129(10):2404-10.

[f]Patient ISD068 and ISD102 are highlighted as patients of interest; Dsg3-specific mAbs are derived and characterized from these two patients.

TABLE S2. Related to Figure 2. Repertoire analysis of Dsg3-specific mAbs isolated from two PV patients.

Patient	mAb[a]	Isotype	Heavy Chain		CDR3 length (nt)	# Mutations (R/S ratio)	Light Chain			CDR3 length (nt)	# Mutations (R/S ratio)
			V gene	J gene			K/L	V gene	J gene		
ISD102 Diagnosis (n=20)	P3F3	IgG1	V_H1-46	J_H3	54	23 (19/4)	Kappa	V_κ2-24	J_κ5	27	8 (7/1)
	P5D5	IgG1	V_H1-46	J_H2	27	25 (20/5)	Kappa	V_κ2-24	J_κ2	27	14 (10/4)
	P5F5[+]	IgA1	V_H3-15	J_H4	54	18 (11/7)	Lambda	V_L6-57	J_L3	30	18 (15/3)
	P5F4[+]	IgA1	V_H3-15	J_H4	54	22 (14/8)	Lambda	V_L6-57	J_L3	30	19 (16/3)
	P5B3[~]	IgG1	V_H3-15	J_H4	54	20 (13/7)	Lambda	V_L3-10	J_L3	33	17 (12/5)
	P5C3[~]	IgG1	V_H3-15	J_H4	54	26 (15/11)	Lambda	V_L3-10	J_L3	33	20 (15/5)
	P5A6[δ]	IgG1	V_H1-46	J_H3	45	14 (9/5)	Kappa	V_κ2-24	J_κ5	27	13 (8/5)
	P5G3[δ]	IgG1	V_H1-46	J_H3	45	18 (13/5)	Kappa	V_κ2-24	J_κ5	27	12 (9/3)
	P5B1	IgG1	V_H1-46	J_H6	54	24 (14/10)	Kappa	V_κ2-24	J_κ1	27	7 (4/3)
	P4D4[β]	IgG1	V_H3-23	J_H1	57	19 (14/5)	Lambda	V_L3-21	J_L3	33	21 (15/7)
	P5G4[β]	IgG1	V_H3-23	J_H1	57	15 (10/5)	Lambda	V_L3-21	J_L3	33	16 (15/1)
	P5D1[β]	IgG1	V_H3-23	J_H1	57	17 (11/6)	Lambda	V_L3-21	J_L3	33	21 (17/4)
	P5A2	IgG1	V_H3-53	J_H4	45	6 (4/2)	Lambda	V_L1-40	J_L3	33	12 (10/2)
	P5G6	IgG1	V_H1-2	J_H4	42	27 (20/7)	Lambda	V_L3-21	J_L2	36	12 (11/1)
	P5E4[Ω]	IgG1	V_H4-61	J_H4	36	19 (11/8)	Lambda	V_L6-57	J_L3	30	14 (11/3)
	P5B6[Ω]	IgG1	V_H4-61	J_H4	36	15 (9/6)	Lambda	V_L6-57	J_L3	30	12 (9/3)
	P5D4	IgG1	V_H3-66	J_H4	33	18 (17/1)	Kappa	V_κ1-5	J_κ2	27	5 (4/1)
	P3A6	IgG1	V_H1-69	J_H3	54	30 (23/7)	Kappa	V_κ3-15	J_κ2	30	11 (9/2)
	P5C2	IgG1	V_H4-4	J_H4	42	17 (11/6)	Kappa	V_κ3-11	J_κ4	27	2 (2/0)
	P5A4	IgM	V_H1-2	J_H4	57	0	Kappa	V_κ3-20	J_κ1	27	0
ISD068 Diagnosis (n=25)	P1E6[*]	IgG4	V_H5-10	J_H4	48	18 (14/4)	Kappa	V_κ2D-29	J_κ4	27	14 (9/5)
	P1C3[*]	IgG4	V_H5-10	J_H4	48	24 (21/3)	Kappa	V_κ2D-29	J_κ4	27	19 (9/10)
	P1D2[±]	IgG1	V_H1-58	J_H3	33	24 (20/4)	Kappa	V_κ3-11	J_κ2	30	12 (9/3)
	P1A1	IgG4	V_H4-34	J_H4	69	27 (25/12)	Kappa	V_κ3-20	J_κ2	27	13 (11/2)
	P1C5	IgG1	V_H4-59	J_H4	60	39 (31/8)	Kappa	V_κ3-15	J_κ1	30	11 (8/3)
	P1C6	IgG1	V_H3-23	J_H3	63	26 (19/7)	Kappa	V_κ4-1	J_κ5	27	16 (10/6)
	P1F1	IgG1	V_H4-39	J_H2	42	29 (21/8)	Kappa	V_κ1-5	J_κ1	27	26 (19/7)
	P1B3[#]	IgG4	V_H4-39	J_H2	84	43 (28/15)	Kappa	V_κ3-15	J_κ2	33	18 (10/8)
	P1F5[#]	IgG1	V_H4-39	J_H2	84	29 (17/12)	Kappa	V_κ3-15	J_κ2	33	20 (16/4)
	P1D6[†]	IgG4	V_H1-2	J_H1	30	22 (14/8)	Kappa	V_κ3-15	J_κ1	33	16 (12/4)
	P2E3	IgG4	V_H1-2	J_H4	30	20 (15/5)	Kappa	V_κ3-11	J_κ2	33	15 (11/4)
	P1G4	IgG1	V_H4-39	J_H2	42	30 (21/9)	Kappa	V_κ1-5	J_κ1	27	20 (14/6)
	P1D3[^]	IgG1	V_H1-2	J_H4	72	29 (24/5)	Kappa	V_κ3-11	J_κ2	33	16 (11/5)
	P2C2[^]	IgG1	V_H1-2	J_H4	72	28 (24/4)	Kappa	V_κ3-11	J_κ2	33	21 (17/4)
	P1B2[%]	IgG1	V_H4-39	J_H2	63	21 (16/5)	Kappa	V_κ1-5	J_κ1	27	20 (14/6)

	P2E4[%]	IgG1	V_H4-39	J_H2	63	21 (15/6)	Kappa	V_K1-5	J_K1	27	20 (12/8)
	P1C2	IgG4	V_H4-39	J_H5	39	30 (19/11)	Kappa	V_K3-15	J_K2	33	15 (13/2)
	P1A2[‡]	IgG1	V_H4-59	J_H2	48	29 (20/9)	Kappa	V_K3-11	J_K2	18	15 (11/4)
	P2C1	IgG1	V_H4-39	J_H4	48	27 (17/10)	Kappa	V_K1-5	J_K1	21	13 (9/4)
	P2B6	IgG1	V_H4-39	J_H4	42	27 (19/8)	Kappa	V_K1-5	J_K1	27	20 (14/6)
	P1E4[&]	IgG1	V_H4-4	J_H5	45	28 (23/5)	Kappa	V_K3-15	J_K3	30	15 (9/6)
	P1B5[&]	IgG1	V_H4-4	J_H5	45	36 (28/8)	Kappa	V_K3-15	J_K3	30	12 (7/5)
	P1A5	IgG1	V_H3-23	J_H4	39	0	Lambda	V_L1-47	J_L2	33	0
	P1G1	IgA1	V_H3-74	J_H3	51	19 (14/5)	Kappa	V_K3-15	J_K3	27	11 (10/1)
	P1E3	IgG4	V_H3-33	J_H3	60	7 (5/2)	Kappa	V_K1-16	J_K4	27	6 (3/3)
ISD068 Pre-Diagnosis (n=25)	P1D4	IgG4	V_H1-18	J_H4	42	16 (11/5)	Kappa	V_K1-9	J_K4	27	8 (7/1)
	P2F2[†]	IgG4	V_H1-2	J_H1	30	25 (18/7)	Kappa	V_K3-15	J_K1	33	17 (13/4)
	P1D5	IgG4	V_H1-2	J_H3	48	23 (14/9)	Lambda	V_L1-51	J_L3	36	8 (5/3)
	P1D6	IgG1	V_H1-2	J_H3	27	31 (18/13)	Kappa	V_K3-11	J_K5	33	13 (8/5)
	P1B2	IgG1	V_H1-2	J_H4	42	36 (25/11)	Lambda	V_L1-47	J_L1	33	21 (10/11)
	P1B6	IgG4	V_H1-2	J_H4	24	16 (10/6)	Kappa	V_K3-20	J_K4	27	12 (10/2)
	P2D1[^]	IgG1	V_H1-2	J_H4	39	22 (17/5)	Kappa	V_K3-15	J_K4	33	15 (12/3)
	P1E3[^]	IgG4	V_H1-2	J_H4	39	17 (15/2)	Kappa	V_K3-15	J_K4	33	12 (10/2)
	P1G5[^]	IgG4	V_H1-2	J_H4	39	24 (14/10)	Kappa	V_K3-15	J_K4	33	12 (10/2)
	P2D6[±]	IgG4	V_H1-58	J_H3	33	10 (9/1)	Kappa	V_K3-11	J_K2	30	8 (6/2)
	P1D1	IgG1	V_H1-69	J_H5	54	28 (22/6)	Kappa	V_K3-11	J_K4	30	5 (4/1)
	P1C4	IgM	V_H3-23	J_H1	54	3 (1/2)	Lambda	V_L3-25	J_L2	33	10 (7/3)
	P2F4	IgG4	V_H3-53	J_H6	42	31 (26/5)	Kappa	V_K1-5	J_K1	27	15 (10/5)
	P1G1[§]	IgG1	V_H4-4	J_H2	72	35 (26/9)	Kappa	V_K3-20	J_K4	30	18 (14/4)
	P1G2[§]	IgG1	V_H4-4	J_H2	72	36 (27/9)	Kappa	V_K3-20	J_K4	30	14 (12/2)
	P2D2[§]	IgG1	V_H4-4	J_H2	72	38 (28/10)	Kappa	V_K3-20	J_K4	30	16 (13/3)
	P2D3[%]	IgA1	V_H4-39	J_H2	63	15 (11/4)	Kappa	V_K1-5	J_K1	27	11 (10/1)
	P2G2[α]	IgG1	V_H4-39	J_H2	42	21 (11/10)	Kappa	V_K1-5	J_K1	27	17 (11/6)
	P1F3[α]	IgG1	V_H4-39	J_H2	42	20 (14/6)	Kappa	V_K1-5	J_K1	27	15 (10/5)
	P2G1[#]	IgG1	V_H4-39	J_H2	84	23 (15/8)	Kappa	V_K3-15	J_K2	33	14 (12/2)
	P2C4	IgG1	V_H4-59	J_H4	60	34 (28/6)	Kappa	V_K3-15	J_K1	30	9 (6/3)
	P1D2[‡]	IgG1	V_H4-59	J_H2	48	27 (20/7)	Kappa	V_K3-11	J_K2	18	16 (11/5)
	P1C5*	IgG4	V_H5-10	J_H4	48	14 (10/4)	Kappa	V_K2D-29	J_K4	27	11 (5/6)
	P1G3	IgG1	V_H5-10	J_H3	51	21 (18/3)	Kappa	V_K1-33	J_K2	27	19 (15/4)
	P1F4	IgG4	V_H5-51	J_H5	75	23 (20/3)	Lambda	V_L3-9	J_L3	30	18 (9/9)

[a] Matching symbols indicate mAbs that are part of the same clonal expansion.

TABLE S3. Related to Figure 6. Characteristics of antibodies selected for germline reversion.

Patient	mAb	Isotype	Heavy Chain		CDR3 length (nt)	R/S Ratio			# unique clones	Epitope	Pathogenicity[a]
			V gene	J gene		VH	FR	CDR			
ISD068 (n=10)	P1C3	IgG4	VH5-10	JH4	48	7	4	9	2	EC1A	+
		Kappa	VK2D-29	JK4	27						
	P1D2	IgG1	VH1-58	JH3	33	5	4.33	6	2	EC1A	+
		Kappa	VK3-11	JK2	30						
	P1A1	IgG4	VH4-34	JH4	69	1.25	1.56	1.67	1	EC2A	-
		Kappa	VK3-20	JK2	27						
	P1B2	IgG1	VH4-39	JH2	42	3.2	3	5	3	EC2A	+
		Kappa	VK1-5	JK1	27						
	P1B3	IgG4	VH4-39	JH2	84	1.87	1.1	6.5	2	EC2A	+
		Kappa	VK3-14	JK2	33						
	P1D6	IgG4	VH1-2	JH1	30	1.75	1.67	2	1	EC2A	-
		Kappa	VK3-15	JK1	33						
	P1F1	IgG1	VH4-39	JH2	42	2.63	2.71	4	3	EC2A	+
		Kappa	VK1-5	JK1	27						
	P2E3	IgG4	VH1-2	JH4	30	3	2.25	6	1	EC2A	+
		Kappa	VK3-11	JK2	33						
	P2C1	IgG1	VH4-39	JH4	48	1.7	0.55	9	1	EC4A	+
		Kappa	VK1-5	JK1	21						
	P1E3	IgG4	VH3-33	JH3	60	2.5	4	1	1	EC4B	-
		Kappa	VK1-16	JK4	27						
ISD102 (n=10)	P3F3	IgG1	VH1-46	JH3	54	4.75	2.75	7	1	EC1A	++
		Kappa	VK2-24	JK5	27						
	P5A2	IgG1	VH3-53	JH4	45	2	2	2	2	EC1A	-
		Lambda	VL1-40	JL3	33						
	P5B1	IgG1	VH1-46	JH6	54	1.86	1.57	1	1	EC1A	++
		Kappa	VK2-24	JK4	27						
	P5G3	IgG1	VH1-46	JH3	45	2.60	2.75	6	2	EC1A	++
		Kappa	VK2-24	JK5	27						
	P5G6	IgG1	VH1-2	JH4	42	2.86	1.25	8	1	EC1 domain	-
		Lambda	VL3-21	JL2	36						
	P5B3	IgG1	VH3-15	JH4	54	1.86	0.71	6	3	EC1B	+
		Lambda	VL3-10	JL3	33						
	P5F5	IgA1	VH3-15	JH4	54	1.57	1.17	4	3	EC1B	+
		Lambda	VL6-57	JL3	30						
	P3A6	IgG1	VH1-69	JH3	54	3.29	2.67	7	1	EC4B	-
		Kappa	VK3-15	JK2	30						
	P5D4	IgG1	VH3-66	JH4	33	17	12	5	2	EC4B	+
		Kappa	VK1-5	JK2	27						
	P5E4	IgG1	VH4-61	JH4	36	1.38	1.14	1.5	2	EC4B	-
		Lambda	VL6-57	JL3	30						

[a] Pathogenicity was determined using the following cut-off values for the dissociation index number: - = DI≤10; + = DI>10; ++ = DI>100.
